# Integration of miRNA and mRNA expression profiles reveals microRNA-regulated networks during muscle wasting in cardiac cachexia

**DOI:** 10.1038/s41598-017-07236-2

**Published:** 2017-08-01

**Authors:** Leonardo N. Moraes, Geysson J. Fernandez, Ivan J. Vechetti-Júnior, Paula P. Freire, Rodrigo W. A. Souza, Rolando A. R. Villacis, Silvia R. Rogatto, Patricia P. Reis, Maeli Dal-Pai-Silva, Robson F. Carvalho

**Affiliations:** 1 Department of Morphology, Institute of Biosciences, São Paulo State University (UNESP), Botucatu, SP Brazil; 2 International Center for Research (CIPE), AC Camargo Cancer Center, São Paulo, SP Brazil; 3 Department of Genetics and Morphology, Institute of Biological Sciences, University of Brasília (UnB), Brasília, DF Brazil; 4 Department of Clinical Genetics, Vejle Hospital and Institute of Regional Health Research, University of Southern Denmark, Vejle, Denmark; 5 Faculty of Medicine, São Paulo State University (UNESP), Brazil, Botucatu, SP Brazil

## Abstract

Cardiac cachexia (CC) is a common complication of heart failure (HF) associated with muscle wasting and poor patient prognosis. Although different mechanisms have been proposed to explain muscle wasting during CC, its pathogenesis is still not understood. Here, we described an integrative analysis between miRNA and mRNA expression profiles of muscle wasting during CC. Global gene expression profiling identified 1,281 genes and 19 miRNAs differentially expressed in muscle wasting during CC. Several of these deregulated genes are known or putative targets of the altered miRNAs, including miR-29a-3p, miR-29b-3p, miR-210-5p, miR-214, and miR-489. Gene ontology analysis on integrative mRNA/miRNA expression profiling data revealed miRNA interactions affecting genes that regulate extra-cellular matrix (ECM) organization, proteasome protein degradation, citric acid cycle and respiratory electron transport. We further identified 11 miRNAs, including miR-29a-3p and miR-29b-3p, which target 21 transcripts encoding the collagen proteins related to ECM organization. Integrative miRNA and mRNA global expression data allowed us to identify miRNA target genes involved in skeletal muscle wasting in CC. Our functional experiments in C2C12 cells confirmed that miR-29b down-regulates collagen genes and contributes to muscle cell atrophy. Collectively, our results suggest that key ECM-associated miRNAs and their target genes may contribute to CC in HF.

## Introduction

Heart failure (HF) is a major public health problem affecting millions of patients worldwide. As the most growing cardiovascular problem, HF affects about 2% of the Western population, with the prevalence increasing sharply from 1% in 40-year-old individuals to 10% above the age of 70 years^1^. It is the most common cause of hospitalization with a poor prognosis similar to diseases such as cancer^1–3^. Cachexia associated with HF, or cardiac cachexia (CC), is a complex metabolic syndrome defined by a weight loss of > 6% over 6 months that accompanies HF in up to 50% of severe cases, being an independent factor of poor prognosis^4, 5^.

The awareness of CC has increased over the last two decades^6^, and several skeletal muscle alterations have been described in patients and animals with this condition; these include atrophy (wasting), shift from slow to fast fibers, decreased oxidative capacity, and increased fatigability^7–11^. Considering the overall complexity of skeletal muscle function regulatory mechanisms, processes that lead to progressive muscle wasting, ultimately resulting in CC, are likely due to deregulation of signaling networks. Previous examinations of large scale mRNA expression in human and rodent skeletal muscle atrophy have generated insights on the molecular changes underlying the loss of skeletal muscle mass in different conditions such as unloading, immobilization, glucocorticoid treatment, diabetes, sarcopenia, starvation, and denervation^12–17^ and revealed new biomarkers of cancer cachexia^18, 19^.

Despite the pathophysiological stimuli for muscle wasting, different types of muscle atrophy may also share common transcriptional programs activated in systemic diseases^20^. Such complexity illustrates the need to apply a global approach to analyzing the molecular changes that occur during wasting in CC. Although global gene expression alterations are informative, the identification of microRNAs (miRNAs) has opened up a new field of investigation to understand molecular regulatory gene expression mechanisms in skeletal muscle diseases^21–24^. Comprehensive miRNA expression profiling has revealed that miRNA expression changes are associated with wasting conditions such as primary muscular disorders, dexamethasone-induced atrophy, fasting, denervation, diabetes, and cancer cachexia^23–26^. However, to our knowledge, no other study has examined the role of miRNAs in skeletal muscle wasting during HF and CC. Therefore, our goal was to perform an integrative, global miRNA and mRNA expression profiling analysis in soleus muscle of rats with CC to unravel novel regulatory networks and molecular pathways involved in muscle wasting. Studies such as this will provide the basis to understand the molecular regulatory mechanisms modulated by miRNAs in CC. Our data may be useful for future development of novel therapeutic approaches for preventing and treating muscle wasting in CC.

## Results

### Monocrotaline (MCT) treatment induces cardiac hypertrophy and failure

As expected, all rats that received intraperitoneal MCT injection developed right ventricular hypertrophy and failure (CC group) compared to control rats injected with saline (CT group) (Table 1). After 30 days of MCT injection, CC group exhibited signs of HF that included strong tachypnea, lack of spontaneous activity, piloerection and cold extremities before sacrifice. These animals also showed HF at post-mortem, confirmed by atrium and right ventricular hypertrophies, pleural and pericardial effusions, and presence of lung and liver congestion. No alterations were found in the control rats. Heart weight was increased in CC compared to CT, as demonstrated by right ventricle weight (RVW), atrium weight (ATW), and by the indexes of cardiac hypertrophy [left ventricle weight (LVW)/body weight (BW), RVW/BW, and ATW/BW]. LVW was decreased in CC compared to CT; however, the LVW/BW index was increased in CC.

**Table 1:** Anatomic data of CT and CC groups.

	CT	CC	P value
BW (g)	344.6 ± 17.5	255 ± 13.7	0.0022
LVW (g)	0.69 ± 0.03	0.60 ± 0.06	0.0152
LVW/BW (mg/g)	1.99 ± 0.17	2.35 ± 0.21	0.0260
RVW (g)	0.20 ± 0.03	0.49 ± 0.04	0.0022
RVW/BW (mg/g)	0.58 ± 0.06	1.94 ± 0.23	0.0022
ATW (g)	0.10 ± 0.01	0.19 ± 0.03	0.0022
ATW/BW (mg/g)	0.28 ± 0.03	0.76 ± 0.11	0.0022
Liver W/D	3.09 ± 0.04	3.53 ± 0.13	0.0002
Lung W/D	4.61 ± 0.19	4.98 ± 0.22	0.0219

Values are mean ± SD; n: number of animals; CT: control group (n = 6); CC: cardiac cachexia group (n = 6); BW: body weight; LVW: left ventricle weight; RVW: right ventricle weight; ATW: atrium weight; W/D, wet-to-dry weight. P value of the statistical significance difference between the groups.

### MCT-treated rats develop CC associated with muscle wasting and fiber-type changes

**Figure 1:**
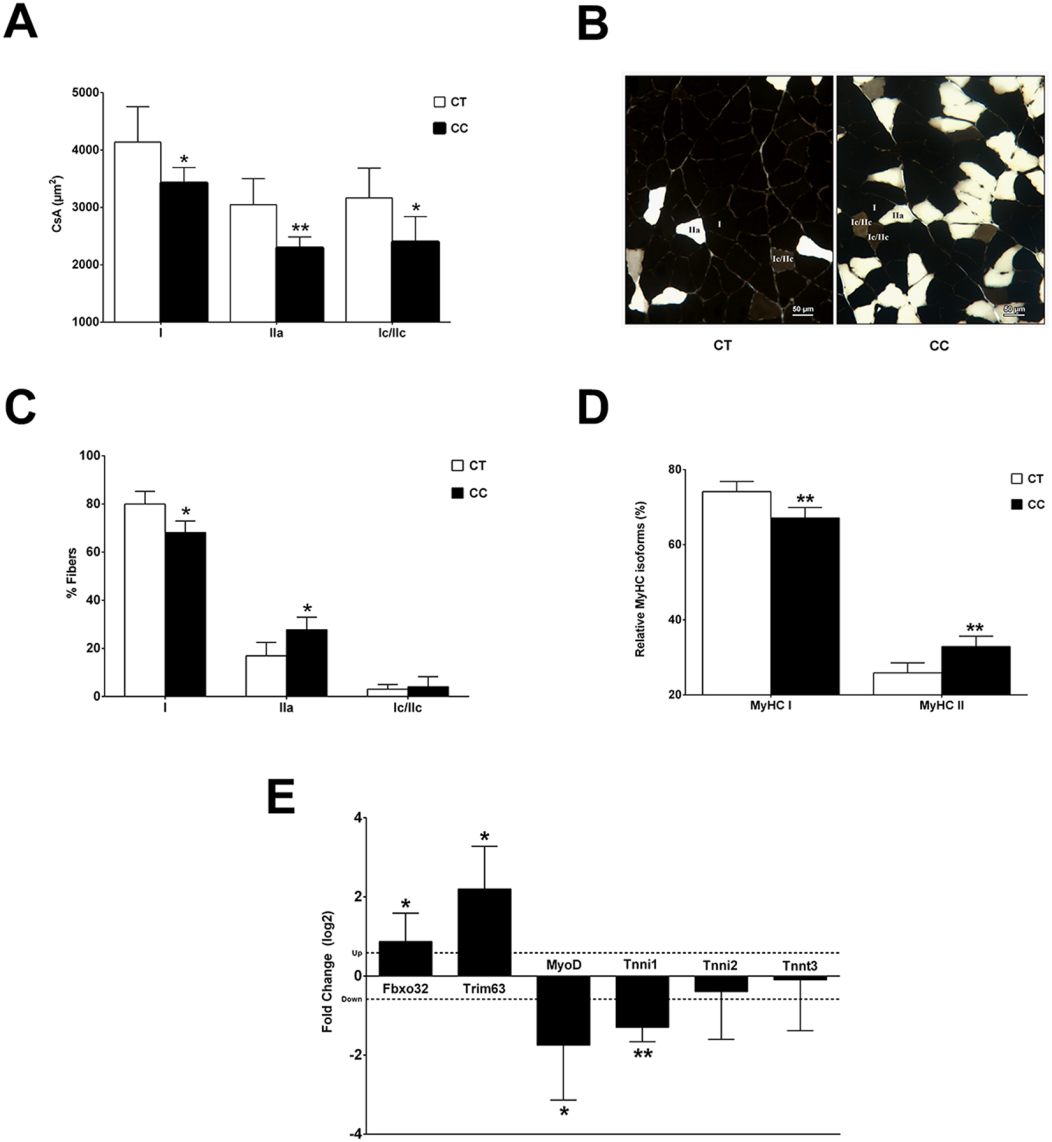
Monocrotaline-treated rats develop right heart failure and cardiac cachexia associated with muscle wasting and fiber-type changes. Representative cross-sections of soleus muscles showing fiber-type distribution using myofibrillar adenosine triphosphatase (m-ATPase) reaction after pre-incubation at pH 4.3, from control (CT) and cardiac cachexia (CC) groups (**A**). CC decreases cross-sectional area (CSA; µm^2^) of fiber-types I, Ic/IIc, and IIa (**B**), induces a slow-to-fast switch in fiber types composition (**C**), and changes the relative myosin heavy chain (MyHC) isoform percentage (%) in soleus muscle (**D**). Expression of atrogenes (*Fbxo32* and *Trim63*), *Myod*, and slow and fast (*Tnni1*, *Tnni2*, and *Tnnt3*) myofiber genes in soleus muscle of CC compared to their levels of expression in CT (set at a value of 0), as detected by real-time PCR. Y-axis represents log2-fold change. The expression of each transcript was normalized by the expression of *Ppib*, *B2m*, and *Ppia*. (**E**) Data are expressed as mean ± SD; n = 6 per group. *p < 0.05; **p < 0.001: statistical significance versus CT group.

Our findings are consistent with previous reports showing skeletal myopathy in HF (reviewed elsewhere^27^). MCT-treated rats develop CC associated with a significant reduction in whole BW (Table 1) and muscle fiber cross-sectional area (CSA; taken as an index of muscle atrophy) (Fig. 1A), confirming that these animals were also cachectic. The histochemical reaction of myofibrillar ATPase (m-ATPase) revealed a significant reduction in fiber CSA of all muscle-fiber types (I, II and Ic/IIc) in CC rats (Fig. 1A,B). These structural abnormality characteristics of skeletal myopathy related to CC also included qualitative changes in the content of muscular fibers secondary with a slow to fast transition in myofibrillar properties (Fig. 1C,D). Expression of the atrogenes *Fbxo32* and *Trim63* was increased in CC compared to CT; however, the expression of the myogenic regulatory factor *Myod1* was decreased (Fig. 1E). Additionally, gene expression analysis demonstrated reduced slow *TnnI1* transcript levels despite no activation in the expression of fast *TnnI2* or *TnnT3* mRNAs in CC group (Fig. 1E).

### Skeletal myopathy in cardiac cachexia is associated with transcriptome changes in key regulatory pathways

In order to understand the transcriptomic changes associated with skeletal myopathy in CC, we performed a global mRNAs expression profiling analysis that identified 1,281 deregulated genes (p ≤ 0.01 and fold change ≥ 1.5), of which 538 and 743 were up- or down-regulated, respectively (Supplementary Table S1). Microarray data are available in Gene Expression Omnibus GSE72701, and the mRNA differentially expressed in CC are provided in the supplementary material. In most cases, a functional class was assigned to these 1,281 regulated genes using the Gene Set Enrichment Analysis (Supplementary Table S2). The top over- and under-expressed genes were selected and ranked by a combination of p-value < 0.05 and fold change ≥ 5, and are listed in Supplementary Tables S3 and S4, respectively.

**Figure 2:**
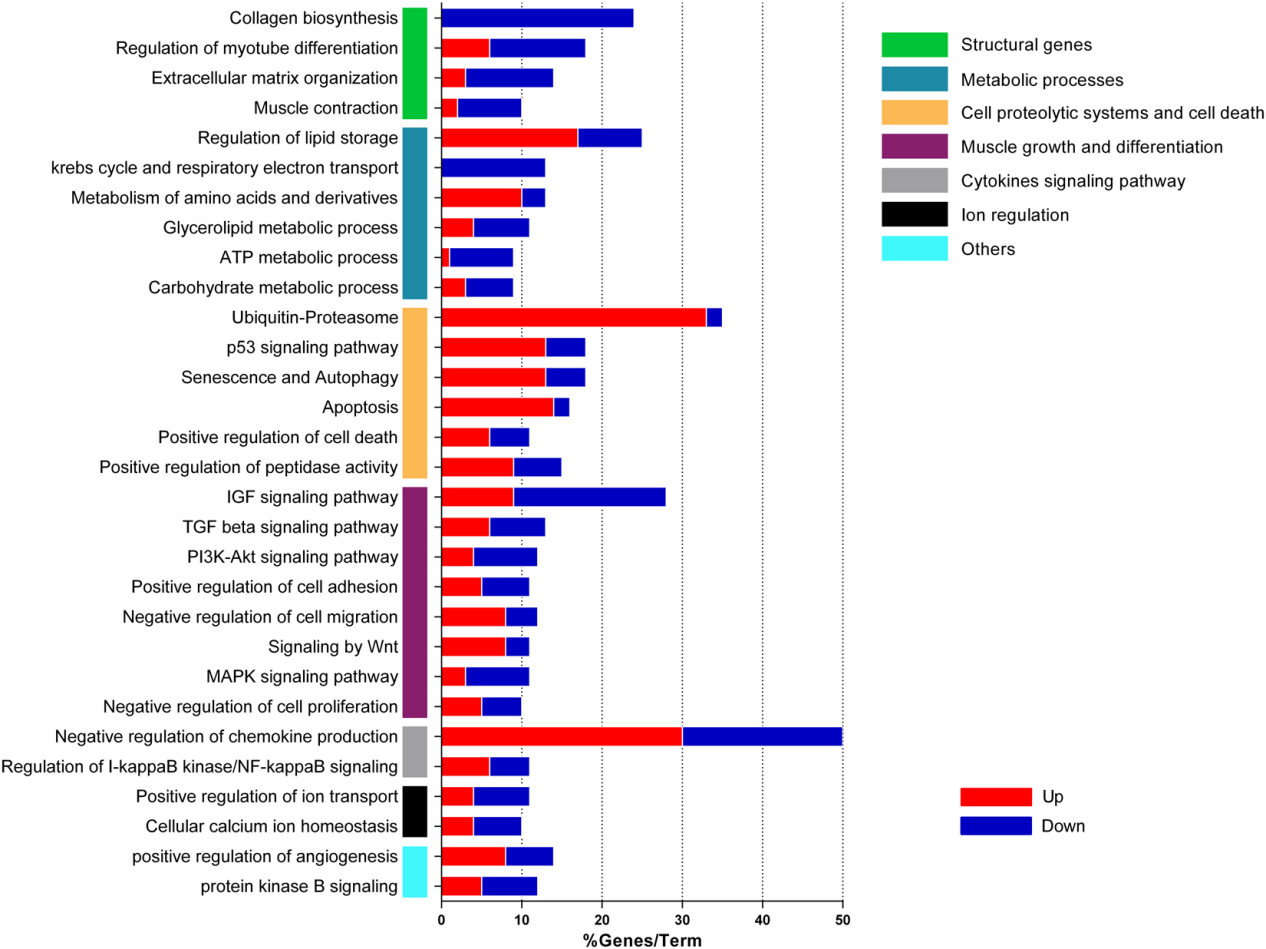
Gene-term enrichment analysis of differentially expressed genes in soleus muscle of cardiac cachexia rats to identify top canonical pathways. Each vertical colored bars (y-axis) represent a major module; horizontal bars represent the percentage of genes presented in the data set compared to the total number of genes in each pathway. Fraction of DE genes in each pathway (up/down, red/blue; respectively) are shown in x-axis. Additional information in Supplementary Table S2.

To determine the biological and functional implications of gene expression changes in muscle wasting during CC, we performed functional enrichment of the differentially expressed genes. This analysis showed that 30 pathways were clustered in biological processes important to skeletal myopathy in HF, which included structural genes (e.g., collagen biosynthesis, regulation of myotubes differentiation, extra-cellular matrix organization, and muscle contraction), metabolic processes, cell death and proteolysis, and muscle growth and differentiation (Fig. 2). Other relevant processes enriched in our dataset included cytokine signaling, ion regulation and angiogenesis (Fig. 2).

To gain further insight into individual pathways, we also analyzed the over- and under-expressed genes in each pathway (Fig. 2). Notably, this analysis showed that all deregulated genes related to collagen biosynthesis were down regulated. The large majority of the deregulated genes related to cell proteolytic systems and cell death pathways were up regulated (Fig. 2).

### miRNAs associated with skeletal myopathy in cardiac cachexia

**Figure 3:**
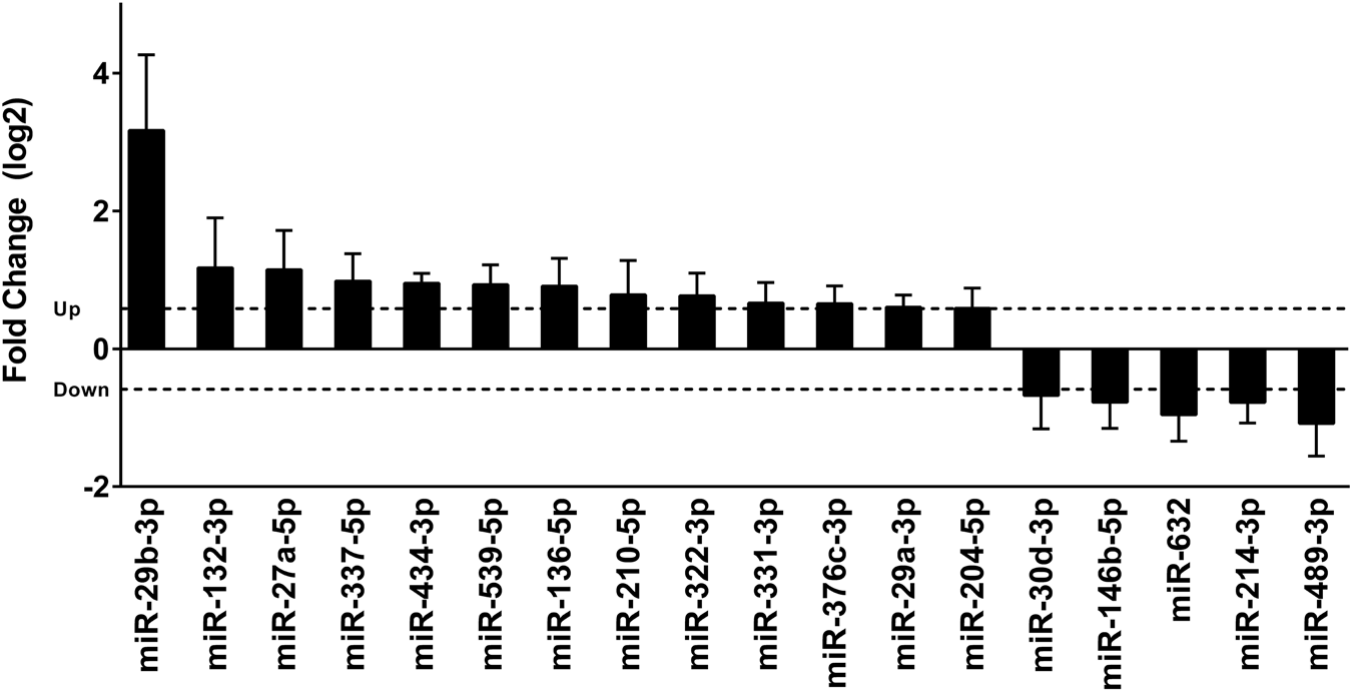
Identification of differentially expressed miRNAs in soleus muscle of cardiac cachexia group (CC) compared to their level of expression in control group (CT; set at a value of 0), as detected by low-density miRNA arrays. Y-axis represents log2-fold change. The dashed line indicates fold change value of 1.5. The expression of each miRNA was normalized by the expression of the small RNAs snoRNA135, Y1, U87, and MammU6. Data are expressed as mean ± SD; n = 6 per group. *p < 0.05: statistical significance versus CT group.

To identify miRNAs as gene expression regulators in skeletal myopathy during CC, we performed a comparative miRNA expression profiling analysis. Eighteen of 373 miRNAs were differentially expressed in muscle wasting during CC compared to controls (13 upregulated and 5 downregulated) (Fig. 3 and Supplementary Table S5).

### Integrative analyses of miRNA and mRNA expression profiles identified signaling pathways enriched with predicted miRNA targets

**Figure 4:**
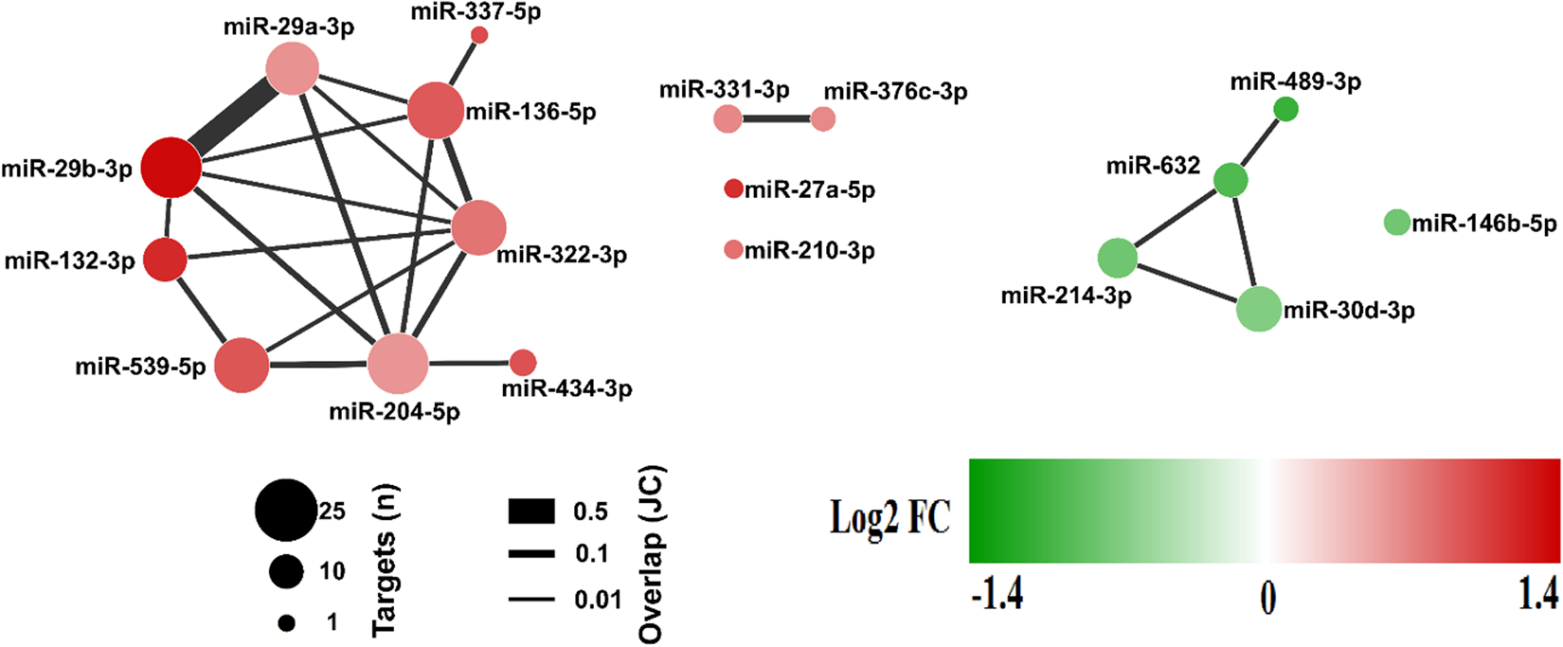
Two distinct sub-networks miRNA-mRNA deregulated in cardiac cachexia. We identified 222 deregulations between 18 miRNAs and 177 target genes. The solid lines connecting molecules represent miRNA-mRNA interaction. Node size represents number of targets for each miRNA, edge width denotes overlap between miRNAs measured by the Jaccard coefficient (JC), and nodes are colored based on the log2 fold change of the differential expression of the miRNA. The interaction network showed two smaller deregulated subnetworks that are clearly separate: one with 8 up-regulated miRNAs (in red) and the other with 4 down-regulated miRNAs (in green) and each miRNA has multiple targets.

Since miRNAs regulate gene expression by both mRNA degradation and translational repression mechanisms, and miRNA-mRNA regulatory networks are complex, we used a parallel miRNA-mRNA expression profile approach as previously described^28–30^ to increase the accuracy of our *in silico* mRNA target prediction used to identify potential mRNA targets of the differentially expressed miRNAs. A dataset of 1,281 genes generated from our mRNA microarray data with predicted and experimentally validated targets were paired to 18 differentially expressed miRNAs in CC. These miRNA–target relationships were predicted by at least four target prediction algorithms. To avoid target multiplicity, we constructed the miRNA target–gene network using differentially expressed genes identified by cDNA microarrays, considering that mRNA and miRNA expression levels should be inversely correlated if one regulates the other. We detected the next topology: 222 deregulations between 18 miRNAs and 177 target genes; the number and the overlap among these predicted targets for each miRNA are represented in Fig. 4.

Interestingly, the miRNAs miR-29a-3p and miR-29b-3p showed the higher number of overlapping targets mRNAs, including many transcripts that encode proteins related to extracellular matrix (ECM) (Supplementary Table S6). Moreover, we found that 95% of the deregulated miRNAs has at least 2 targets genes and that 10 deregulated miRNAs have at least 10 targets genes (Supplementary Table S7). Indeed, we have found that the transcripts *Fbxw7*, *Dnmt3a*, and *Ppic* are co-deregulated by 3 or more miRNAs; including miR-29a-3p and miR-29b-3p (Supplementary Table S8). As also shown in Fig. 4, most miRNA/mRNAs deregulations are connected, and generate a large connecting network. These results indicate an implicated combination of target multiplicity and miRNA cooperativeness during muscle wasting in CC.

**Figure 5:**
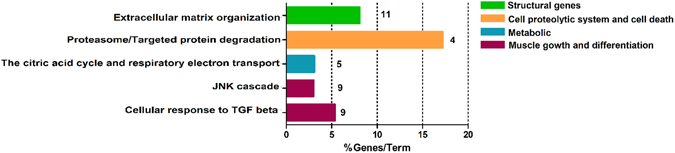
Gene-term enrichment analysis of mRNAs regulated by miRNAs in cardiac cachexia. Top canonical pathways affected in cardiac cachexia. Bars represent percentage of genes altered by miRNAs present in the data set compared to the total number of genes present in each selected pathway. In front of each bar is indicated the number of altered miRNAs in the pathway. Each color represents a major module.

Based on miRNA-target deregulated network analysis described above, we identified enriched pathways for target genes deregulated by differentially expressed miRNAs. As shown in the Fig. 5 and Supplementary Tables S4, 5 pathways were enriched. Statistical comparisons revealed miRNA interactions affecting genes regulating ECM organization (*P* = 9.2E-5), proteasome protein degradation (*P* = 3.2E-5), citric acid cycle and respiratory electron transport (*P* = 5.6E-4), JNK cascade (*P* = 3.4E-4) and cellular response to TGF-β (*P = *7.8E-3). Interestingly, this analysis also revealed 11 miRNAs, including miR-29a-3p and miR-29b-3p, which target 21 transcripts encoding proteins related to ECM, comprising the collagens *Col1a1*, *Col1a2*, *Col22a1*, *Col3a1*, *Col5a1*, and *Col6a2* (Supplementary Table S4).

**Figure 6:**
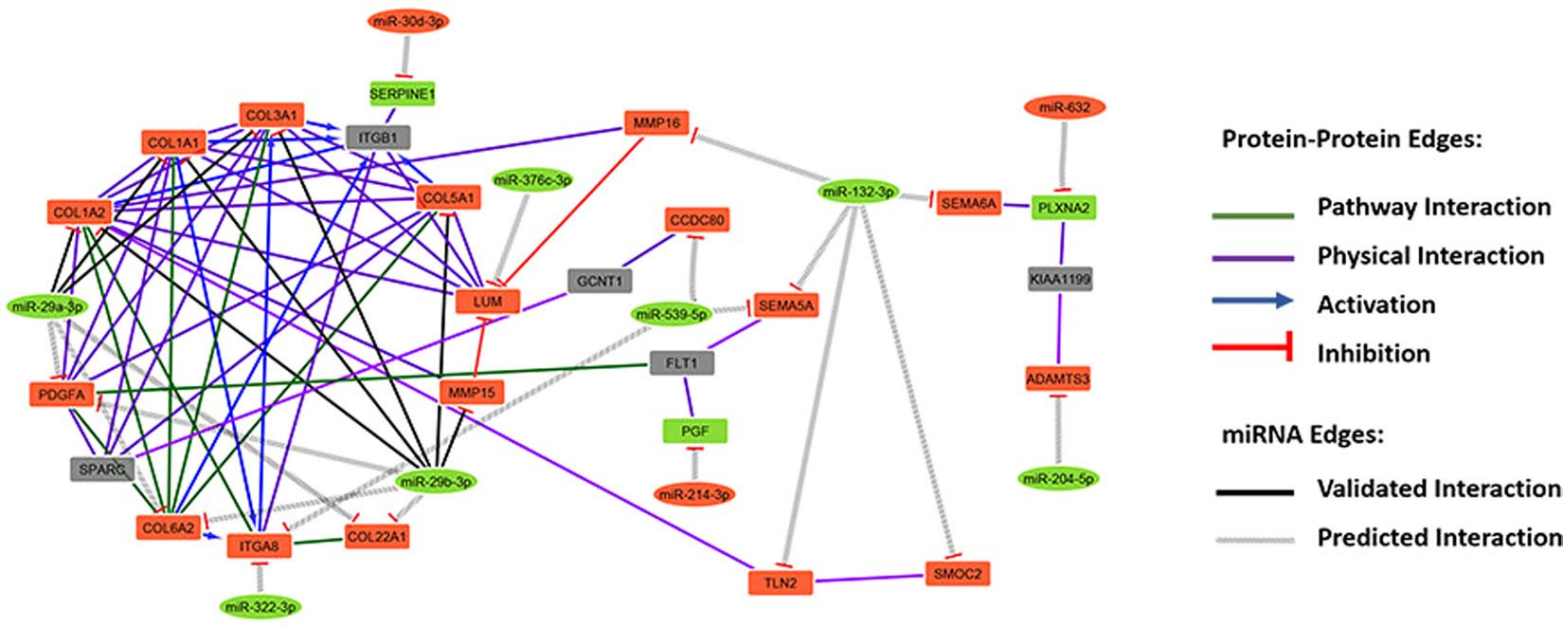
Complexity of the miRNA-mRNA interactome network in rat soleus muscles with cardiac cachexia. The regulatory network displays predicted and validated interactions between miRNAs (rectangle) and mRNAs (elliptic) deregulated from TLDA and microarray experiments generated by using a fold-change cutoff <1.5, P < 0.05 and enrichment with physical and pathway protein-protein interaction by Cytoscape database.

To elucidate the functions of these complex interactions between mRNAs and miRNAs in the ECM network, we further examined probable miRNA-mRNA interactions. The complexity of the miRNA-mRNA interactome of muscle wasting in CC is demonstrated by a regulatory network displaying predicted and validated interactions between deregulated miRNAs and target mRNAs that are enriched, considering physical and pathway protein-protein interactions using Cytoscape database (Fig. 6). This analysis further confirmed the importance of miR-29 family members and their interaction with ECM protein coding transcripts. Our data highlight, for the first time, a set of miRNAs that targets transcripts that encode ECM organization proteins in muscle wasting during CC.

### Extracellular matrix remodeling in cardiac cachexia

**Figure 7:**
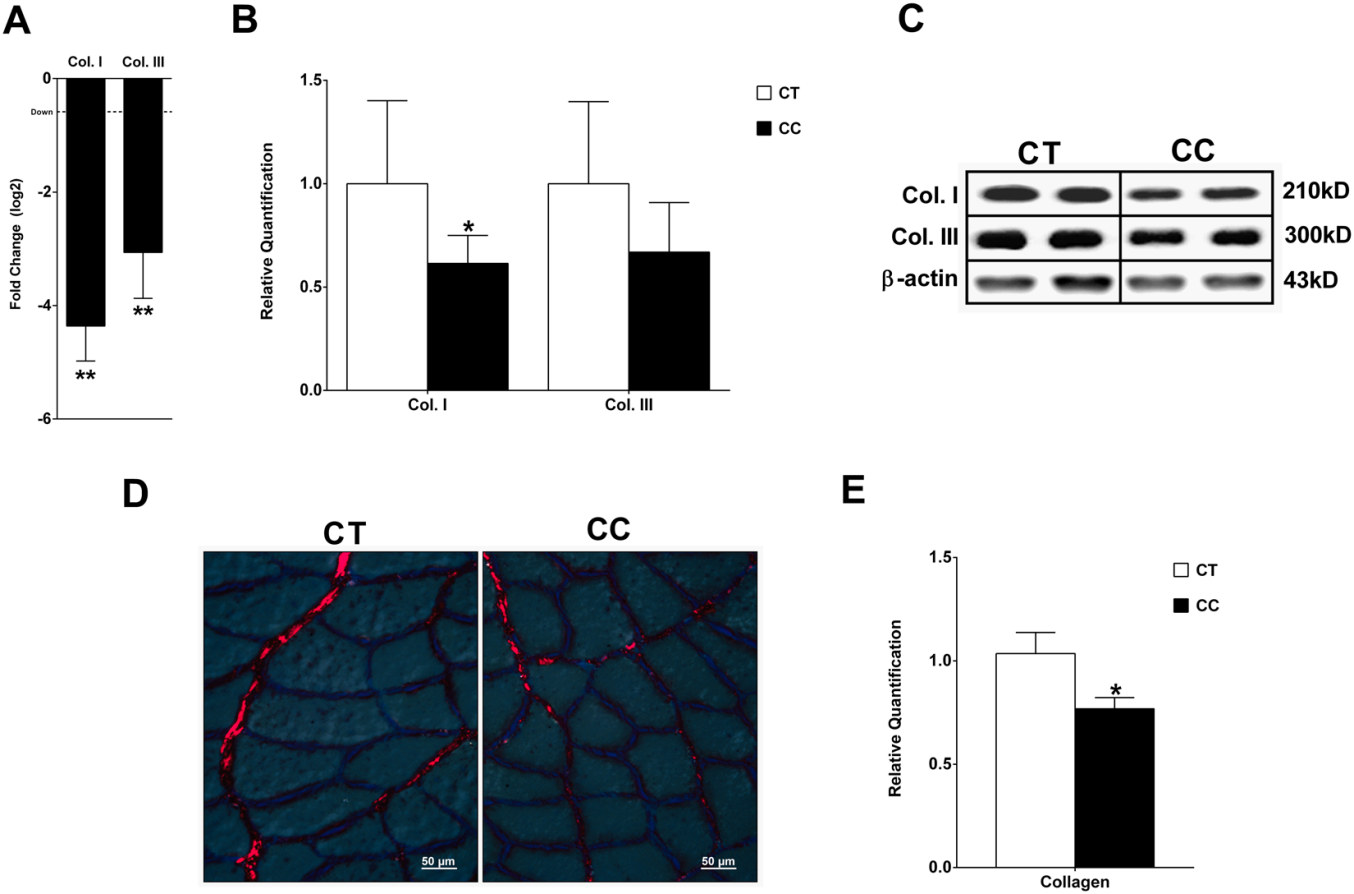
Decreased collagen in the extracellular matrix in the soleus muscle of rats with cardiac cachexia. Analysis of type I collagen gene expression by real-time PCR (in log 2) normalized by *Ppib*, *B2m*, and *Ppia* (**A**). Western blot of collagen I and III normalized by β-actin (**B**,**C**). Soleus muscle cross-sections stained with Sirius red and analyzed in microscope under light polarized (**D**). Mean gray scale level of Picrosirius red stained collagen fibers in the soleus muscle expressed as a percent of the mean gray scale level of collagen in the endomysium (**E**). Data are expressed as mean ± SD; n = 6 per group. *p < 0.05; **p < 0.01: statistical significance versus CT group.

**Figure 8:**
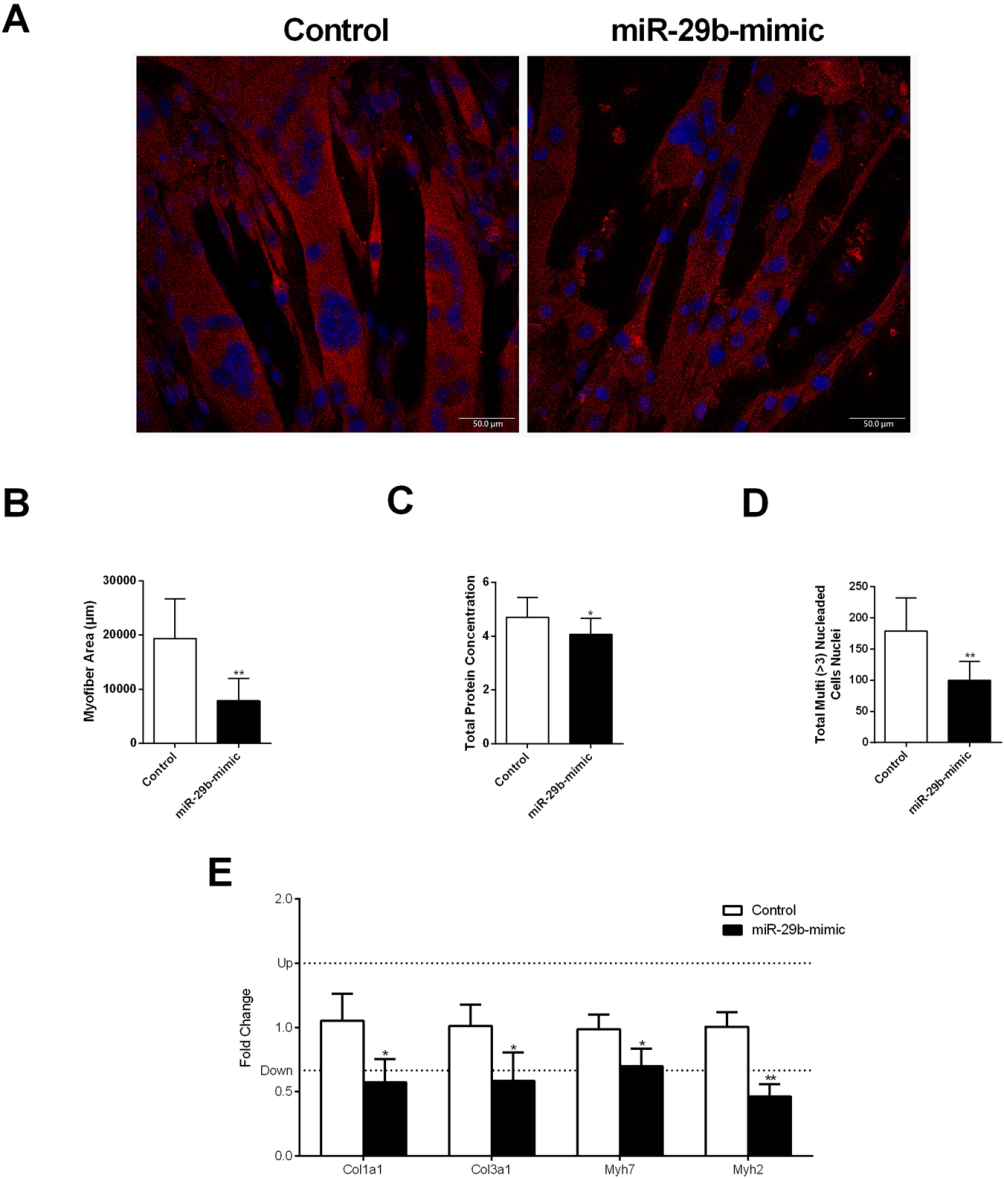
miR-29b promotes muscle atrophy and reduces collagens expression. Immunofluorescence of C2C12 myotubes transfected with miR-29b-3p-mimic stained with antibodies specific to myosin heavy chain Myh2 (red). Nuclei stained with DAPI (blue) **(A)**. Quantitative analyses of myotubes area **(B)**. Total protein concentration in C2C12 myotubes measured by the Bradford assay **(C)**. Number of total myotubes nuclei (three or more nucleus/cells) **(D)**. mRNA levels of *Col1a1*, *Col3a1*, *Myh7*, and *Myh2* in myotubes after miR-29b-mimic transfection compared to their levels of expression in CT, as detected by real-time PCR. The expression of each transcript was normalized by the expression of *Ppia* and *Ppib* **(E)**. Data represent the average of three independent experiments and are expressed as mean ± SD. *p < 0.05; **p < 0.001: statistical significance versus CT group.

The high degree of consistency in the pathway enrichment analyses clearly suggests that CC induces coherent interactions between the miRNAs and mRNAs involved in ECM. The ECM remodeling was confirmed by changes in both collagen gene (Fig. 7A) and protein expression (Fig. 7B,C). Corroborating these data, the cross-sectional analysis of the soleus muscle stained by Picrosirius technique demonstrated a relative decrease of collagen content in the ECM (Fig. 7D,E). Next, we asked whether miR-29b mediates the repression of *Col1a1* and *Col3a1* expression with a functional consequence in muscle cells atrophy. To test this, we transfected a synthetic miR-29b mimic into C2C12 myoblasts that were further fully differentiated in myotubes. C2C12 myotubes transfected with miR-29b mimic had a significant reduction in myotubes area (Fig. 8A and B), total protein concentration (Fig. 8C), number of myotubes (three or more nucleus/cells; Fig. 8D), and *Myh7* and *Myh2* expression (Fig. 8E). The miR-29b overexpression also repressed *Col1a1* and *Col3a1*transcript levels (Fig. 8E).

## Discussion

To the best of our knowledge, ours is the first study performing an integrated global miRNA and mRNA expression profiling in muscle of rats with CC to unravel novel regulatory networks and molecular pathways involved in muscle wasting. Our results highlight miRNA-regulated gene networks involved in skeletal muscle wasting in CC. Specifically, our results suggest that key ECM-associated miRNAs and their target genes may contribute to CC in HF.

Structural abnormality characteristics of skeletal myopathy have been described in CC^11, 31, 32^; however, transcriptome profile changes associated with CC remain widely unknown. We have produced a global transcriptome catalogue of muscle wasting in CC that identified 1,281 differentially expressed genes, of which 538 and 743 were up- or down-regulated, respectively. Among these, we selected the top 23 deregulated genes (p-value < 0.05 and fold change ≥ 5); from which, 8 genes were up regulated. These most highly expressed genes revealed the metallothioneins (*Mt1m*, *Mt1*, and *Mt2a*), which are significantly increased in human skeletal muscle after 48 h immobilization^33^; these metallothioneins have also been shown to mediate protective adaptations in soleus muscle following disuse mediated by spinal cord injury^34^ and oxidative stress protection in a mouse model of dystrophinopathy^35^. It is also noteworthy that *Cyp2e1*, which has been shown to impair *GLUT4* gene expression and function in muscle cells^36^, had the highest fold change (46.6X) among the upregulated genes. In addition, 15 genes were downregulated, including mRNAs encoding ECM (*Col1a1*, *Col3a1*, and *Mfap4*), neuromuscular junction (*Ky* and *Rab3a*), IGF-1 pathway (*Dok5* and Igfbp5), and myoblast proliferation proteins (*Mlf1*, *Nrep*, and *Sfrp4*); these findings are consistent with muscle fiber regeneration and ECM remodeling.

Category analysis of the differentially expressed genes showed up-regulation of genes for catabolism (e.g., ubiquitin-proteasome, p53 signaling pathway, autophagy, and apoptosis) and the suppression of structural genes (e.g., collagen biosynthesis, regulation of myotubes differentiation, ECM organization, and muscle contraction). Our results are consistent with a previous meta-analysis on gene expression signatures pertaining to different types of muscle atrophy^37^. These authors described six functional pathways that occupy central positions in the molecular network obtained by the integration of atrophy transcriptome and interaction data. Similar to our study, pathway analysis of different types of muscle atrophy transcriptome indicated that deregulated genes in atrophy conditions are mainly involved in ECM reorganization, cytoskeleton organization, cytokines signaling, and apoptosis pathways^37^. Our results are also in agreement with a previous transcriptome analysis of skeletal muscle wasting performed in a transgenic mouse model of HF and CC raised by sustained activation of Met Tyrosine Kinase in the heart^38^. These authors identified 107 differentially expressed genes (fold change > 1.7) in CC, and the functional categories and pathways in which these differentially expressed genes were classified presented some shared core molecular mechanisms with our CC model. These include genes associated with muscle metabolism, growth, protein synthesis, and inflammation.

Undoubtedly, the formation, maintenance, and physiological and pathophysiological responses of skeletal muscles, with all their complex regulatory circuits, are subject to regulation by miRNAs. To our knowledge, ours is the first study that analyzed genome-wide profiling of miRNA expression during muscle wasting in CC. Using this analysis, we identified 18 altered miRNAs; 13 were upregulated and 5 downregulated. Previous studies have also reported alterations of global miRNA expression in muscle atrophy in primary muscle disorders^23^, diabetes^24^, denervation^24^, dexamethasone-induced atrophy^25^, fasting^24^ and cancer cachexia^24^. The comparison of our CC miRNA profile with these previous studies did not reveal any similar miRNA profile but instead identified a specific subset of CC miRNAs. This is in line with the work by Soares *et al*., 2014^39^ who did not find a common signature of miRNAs in different atrophic models (starvation, denervation, diabetes, and cancer cachexia). Previously, several miRNAs have also been implicated in pathological cardiac hypertrophy and HF in humans and in mouse models of heart disease (reviewed elsewhere^40^). In this sense, our findings in the skeletal muscle are somewhat similar to what has been found in cardiac muscle in which miRNAs add an additional layer of regulation in muscle remodeling during HF.

Among the regulated skeletal muscle miRNAs in CC, we identified two upregulated miRNAs (miR-337-5p and miR434-3p,) that are located in the same cluster (<10 kb) and have very similar fold change values (1.98 and 1.94, respectively). Also notable, we identified miR-29b-3p, miR-29a-3p, miR-210-3p, miR-214, and miR-489, which had been previously reported as involved in the regulation of myogenesis^41–45^. Specifically, high level of miR-29 is important for driving myogenic differentiation, and loss of miR-29 promotes transdifferentiation of myoblasts into myofibroblasts by targeting extracellular molecules including collagens^46, 47^. In fact, previous studies have demonstrated the miR-29 family as a “master fibromiRNA” regulator of the liver, lung, skin, kidney, heart, and skeletal muscles fibrosis^48–55^. Multiple transcripts encoding standard ECM proteins such as collagens, fibrillins and elastin have been implicated as miR-29 family direct targets^49–53^. In C2C12 muscle cells, the stable over-expression of miR-29 down-regulates ECM and cell adhesion genes^46^. Most recently, Galimov *et al*., 2016^43^ used next generation RNA sequencing from miR‐29a knockout myoblasts to identified members of the basement membrane as the most abundant miR‐29a targets. This same study also showed that miR-29 can initiate muscle cell senescence leading to aging-induced atrophy by suppressing the expression of several mediators of cell proliferation and muscle growth. Furthermore, *in vivo* studies have shown that intramuscular injection of miR-29 into dystrophic limb muscles down-regulated collagen and elastin mRNA expression^53^, whereas the systemic delivery of miR-29 mimics led to significant improvement of dystrophic diaphragm muscle by reducing existing fibrosis and increasing regeneration^47^. Thus, the upregulation of the miRNAs miR-29b-3p and miR-29a-3p in our model of CC suggest that they may have an important role in ECM remodeling in this condition.

In order to reduce the complexity of predicted miRNA-mRNA interactions identified by in silico prediction, and to increase the list of miRNAs targets likely associated with muscle wasting in CC, we applied an integrated and simultaneous mRNA and miRNA analysis. This strategy enabled us to identify biologically relevant and experimentally validated miRNA target genes and provided a comprehensive picture of molecular networks regulated by the identified miRNAs. Specifically, this analysis identified mRNAs and miRNAs that play pivotal roles in modulating diverse important biological processes in the skeletal muscle such as proteasome protein degradation (*P = *3.2E-5), citric acid cycle and respiratory electron transport (*P = *5.6E-4), JNK cascade (*P = *3.4E-4), cellular response to TGF-β (*P = *7.8E-3) and, especially, ECM organization (*P = *9.2E-5). Our results showed that, combined with metabolic alterations, different degradation systems and ECM remodeling are key events that likely contribute to skeletal muscle wasting in CC.

Several mechanisms have been proposed to explain the reduced tolerance to exercise during cardiac failure, focusing largely on muscle fibers intracellular alterations. The data obtained in our model, right ventricular pressure overload, described in details mRNAs and miRNAs alterations that may contribute to alterations in muscle endomysium during CC. These results were further confirmed by a dramatic decrease in collagen deposition, demonstrating an actively remodeling of the ECM during skeletal muscle wasting in CC. These results differ from Filippatos *et al*., 2003^56^ who found increased fibrosis in quadriceps muscle of HF patients with CC. Other few studies have analyzed the changes in the skeletal muscle ECM in CC; these alterations mainly involve enhanced metalloproteinase (MMP) activity and collagen content^56–58^. Interestingly, our data agree with recent studies that showed thickening of endomysium and downregulation of several ECM gene transcripts in muscle wasting in cancer cachexia^59, 60^. Considering the isolated effect of miR-29b in C2C12 myotubes, our functional experiments also corroborate with previous studies in C2C12 cells demonstrating that miR-29b has an anti-fibrogenic effect by down-regulating collagen genes^46, 47^ and contributes to muscle atrophy^61^.

To summarize, we have discovered deregulated miRNAs and their target mRNAs in CC that modulate important biological processes in the skeletal muscle, such as proteasome protein degradation, citric acid cycle and respiratory electron transport, JNK cascade, cellular response to TGF-β and, importantly, ECM organization. In addition, our data showed that 11 miRNAs, including miR-29a-3p and miR-29b-3p, target 21 transcripts encoding proteins related to ECM, comprising the collagens *Col1a1*, *Col1a2*, *Col22a1*, *Col3a1*, *Col5a1*, and *Col6a2*. Furthermore, the up-regulated miR-29a-3p and miR-29b-3p had the higher number of overlapping targets mRNAs, including target transcripts that encode ECM proteins. Herein, our integrative miRNA and mRNA analysis highlight miRNA candidates to regulate genes that may contribute to the cachectic state observed in HF.

## Methods

### Cardiac cachexia model

Right HF was experimentally induced in 6 male Wistar rats, 250–300 g of body weight, by a single intra-peritoneal (ip, 60 mg/kg) injection of monocrotaline (MCT, Sigma-Aldrich, Germany), following the procedure described by Gary-Bobo *et al*., 2010^62^. MCT is a pyrrolizidine alkaloid that induces pulmonary vascular disease with severe right ventricle hypertrophy and failure^63^. The monocrotaline-induced pulmonary hypertension experimental model was used since CC is associated with right ventricular failure^64^. Moreover, this model stands out with rapidly progressive right HF and CC^65^, thus allowing an enhanced sensitivity of detection due to larger magnitude of change in a short time frame^65^. Six controls rats (CT group) were injected with saline and were given the same quantity of food as consumed on the previous day by the rats in the treatment group (CC).

CC and CT rats were studied 30 days after MCT administration when the HF group had developed overt HF. Upon anesthesia with intraperitoneal sodium pentobarbital (50 mg/Kg), animals were euthanized and body weight (BW) was evaluated. Soleus muscles were excised, immediately frozen in liquid nitrogen, and stored at −80 °C. Left ventricle weight (LVW), right ventricle weight (RVW), and atrium weight (ATW) normalized by body weight (BW) were used as indexes of heart hypertrophy. Fragments of liver and lung were weighed before and after drying sessions (65 °C for 72 h) to evaluate wet/dry weight ratios. All experiments were carried out in accordance with the Guide of the Institute of Biosciences, UNESP, Botucatu, SP, Brazil. The protocol was approved by the Institute of Biosciences, UNESP, Botucatu, SP, Brazil (Protocol # 201).

### Histochemical and morphometric procedures

Soleus histological sections (12 µm thick) from CC (n = 6) and CT (n = 6) were obtained in a cryostat JUNG CM1800 (Leica, Germany) at −24 °C to determine muscle fiber-type frequency and cross-sectional area (CSA), using myofibrillar adenosine triphosphatase (m-ATPase) histochemistry after pre-incubation at pH 4.35. Muscle fiber types were classified as Types I, Ic/IIc, and IIa. Fiber CSA for each fiber type, and fiber-type frequencies were determined using Image Analysis System Software (Leica, Germany). At least 200 fibers at different points of soleus muscle were measured and their frequency was expressed as the number of fibers per type against the total number of fibers measured.

### Electrophoretic analysis of myosin heavy chain

Myosin heavy chain (MyHC) isoform analysis was performed by sodium dodecyl sulphate polyacrylamide gel electrophoresis (SDS-PAGE) in triplicate. Twelve histological sections (12 µm thick) of CC (n = 6) and CT (n = 6) were collected from each whole muscle sample. The gels were stained with Coomassie Blue and used to identify the MyHC isoforms (MHC I and MyHC IIa) according to their molecular weight. The gels were photographed and densitometry analysis was performed using ImageMaster VDS Software v. 3.0 (GE, USA) to determine relative MyHC isoform content.

### RNA preparations

Total RNA was isolated using TRIzol reagent (Life Technologies, USA) as described by the manufacturer. Total RNA was solubilized in nuclease free-water and treated to eliminate genomic DNA contamination with DNA-free kit (Life Technologies, USA) as described by the manufacturer. Total RNA quantity was determined by the A 260 nm/A 280 nm and A 260 nm/A 230 nm ratios (acceptable when both ratios were > 1.8). RNA Integrity was ensured by obtaining a RNA Integrity Number - RIN > 8 with Agilent 2100 Bioanalyzer (Agilent Technologies, Germany).

### Expression profiling of miRNAs and reference genes

miRNA and mRNA was reverse transcribed using the Megaplex RT Primers Pools A and B and High Capacity RNA-to-cDNA master mix (Life Technologies, USA), respectively. Global miRNA profiling of CC (n = 6) and CT (n = 6) samples was performed with the TaqMan® Array Rodent MicroRNA Cards A and B v3.0 (Life Technologies, USA) for 373 mature miRNAs in rats. The expression profiling of 16 mRNAs commonly used as references genes were evaluated by using the TaqMan® assays Low Density Array Endogenous Control Panel (Life Technologies, USA) to determine the most stable reference genes. miRNA and mRNAs quantitative PCR (qPCR) analyses were performed as described by the manufacturer and run on the ViiA™ 7 Real-Time PCR System. Finally, raw data from each card set was retrieved and imported into Expression Suite Software v1.0.3 (Life Technologies, USA). The small RNAs snoRNA135, Y1, U87, and MammU6 were selected as reference control genes to normalize the miRNA data and genes *B2m*, *Ppia*, and *Ppib* to further normalize mRNA data based on geNorm calculations^66^. Relative quantification of miRNA expression was evaluated using the comparative quantification method^67^. Cutoffs for significant changes were a fold-change > 1.5 and a p-value ≤ 0.05.

### Quantitative analyses of gene expression by real-time reverse transcription polymerase chain reaction (RT-qPCR)

RT-qPCR was carried out with GoTaq® qPCR Master Mix (Promega, USA), using specifics primers (Supplementary Table S9) and cDNA of each sample of CC (n = 6) and CT (n = 6) groups. Reactions were set up in a total volume of 20 µL and performed in the ABI Prism 7300 real-time PCR system (Life Technologies, USA) as described by the manufacturer. Relative quantification of mRNA expression by SYBR green I were assessed by using REST software 2009 v2.0.13, using the pair-wise fixed randomization test with 10,000 permutations^68^, with PCR efficiencies calculated by linear regression from fluorescence increase in the exponential phase in the program LinRegPCR v11.1^69^. Cutoffs for significant changes were a fold-change > 1.5 and a p-value ≤ 0.05.

### Global gene expression profiling analysis

Gene expression profiling of CC (n = 3) and CT (n = 3) groups was performed using the Rat Gene 1.0 ST Array platform (Affymetrix, USA) that covers 17,061 RefSeq transcripts, according to the manufacturer’s instructions. The Ambion WT Expression Kit (Life Technologies, Carlsbad, CA, USA) was used to cDNA synthesis and cRNA amplification, while the fragmentation and labeling procedures were performed with the Affymetrix GeneChip WT Terminal Labeling Kit. Arrays hybridization, washing and scanning were carried on the Affymetrix GeneChip Hybridization Oven 645, Fluidic Station 450 and Scanner 3000 7 G, respectively. Quality control and probe set summarization to attain gene-level signal data was provided by Affymetrix Expression Console software. Data analysis was performed with the R language (v.2.13.0). Background correction and quartile data normalization were applied using RMA (Robust Multi-array Average) algorithm^70^. The limma Bioconductor package^71^ was used to identify differential expressed genes (DEG). Cutoffs for significant changes were a fold-change > 1.5 and a p-value ≤ 0.05.

### Western Blot analysis

Protein levels of soleus muscle of CC and CT samples were analyzed by Western blotting using antibodies specific for collagen I (1:100) (sc-25974, Santa Cruz) and collagen III (1:5000) (ab6310, Abcam). Protein levels were normalized by the endogenous β-actin (1:1000) (sc-81178, Santa Cruz). Muscle protein was extracted using Tris-Triton buffer (10 mM Tris pH 7.4, 100 mM NaCl, 1 mM EDTA, 1 mM EGTA, 1% Triton X-100, 10% glycerol, 0.1% SDS, 0.5% deoxycholate) containing Protease Inhibitor Cocktail (Sigma- Aldrich, USA) and quantified by the Bradford method^72^. Samples were separated on a polyacrylamide gel and then transferred to a nitrocellulose membrane. After blockage, membranes were incubated with the primary antibody. Membrane was washed with TBS-T and incubated with secondary peroxidase-conjugated antibody (1:2500). Super Signal® West Pico Chemiluminescent Substrate (Pierce Protein Research Products, Rockford, USA) was used to detect bound antibodies.

### Pathway and gene ontology enrichment analysis

To further understand the biological relevance of differential expressed genes, we performed functional enrichment analysis in the context of the Gene Ontology (GO) categories, Kyoto Encyclopedia of Genes and Genomes (KEGG) and Reactome databases. A p-value cut-off of 0.001 was used to identify enriched processes. A kappa score was calculated to reflect the relationships between the terms based on the similarity of their associated genes, PSIQUIC web services with the threshold set at 0.3. was used to provide a comprehensive view on the relevant pathways using experimental and *in silico* data from gene networks, protein–protein interactions, and functional interactions^73, 74^. Networks were visualized and analyzed with Cytoscape^75^.

### miRNA – target gene network

Candidate miRNA–target relationships were assessed by at least four target prediction algorithms (union set) extracted from: mirDB, TargetScan 5.1 (conservation and non-conservation sites) (www.targetscan.com), DIANA-microT-CDS v5^76^, miRWalK v2.0^77^, and miRanda^78^. Additionally, we used validated targets deposited in miRTarBase^79^, miRecords^80^, and miRwalK^81^. To avoid target multiplicity, we constructed miRNA target–gene networks considering differentially expressed genes identified by cDNA microarray experimental data.

### Determination of collagen content

Collagen content in soleus muscle was determined using Picrosirius red staining. Briefly, transverse cryosections (12 μm thick) of all samples were placed on the same slide to minimize staining differences; sections were incubated with saturated picric acid solution followed by Picrosirius red (0.1% Sirius red in saturated picric acid) for 3 min., dehydrated and mounted in Permount. Eight color pictures per sample were captured using the microscope with polarized light (400X magnification). Light intensity and filters alignment parameters used were the same for all samples. Quantitative analysis of endomysium collagen type I staining intensity was determined by measuring the grayscale with the Image Analysis System Software (Leica, Germany). The gray values were normalized by mean fiber area.

### Oligonucleotides and transfection

The miR-29b-3p mimic and the respective negative control were complexed with Opti-MEM reduced serum medium (Thermo Fisher Scientific, USA) before transfection. C2C12 myoblasts transfections were performed, when the cells were at approximately 80% confluent, with RNAiMAX lipofectamine (Thermo Fisher Scientific, USA) combined with 30 nM of each oligonucleotide for 15 h. Myoblasts were switched to medium containing 2% horse serum to induce differentiation into myotubes. Myotube area, total protein concentration, number of myotubes, and gene expression were analyzed after 5 days of differentiation.

### Immunostaining

C2C12 myotubes cultured in 6-well plates were fixed in 4% paraformaldehyde for 15 min, washed with PBS and 0.1% TritonX-100 (Sigma, USA), and blocked with 3% BSA, 1% glycine, 8% fetal bovine serum in PBS and 0.1% TritonX-100 for 1 h at room temperature. Subsequently, the cells were incubated with primary (Myh) antibodies overnight at 4 °C and, after washing, the cells were incubated with secondary antibodies for 1 h at room temperature and counterstained with DAPI. All images were acquired at room temperature by scanning confocal microscope TCS SP5 (Leica Microsystems, UK). Myotubes area and number of nuclei were measured by ImageJ software (National Institutes of Health, USA).

### Statistical analysis

Data were expressed as mean ± standard deviation (SD). Statistical analysis was performed using the GraphPad Prisma software v 6.07 (GraphPad Software, Inc., USA). For all statistical analyses not described elsewhere, we used an unpaired, Mann Whitney test. Statistical significance was considered achieved when the *p*-value was <0.05.

## Electronic supplementary material

Supplementary Information
