# Supplementary material for: Integration of miRNA and mRNA expression profiles reveals microRNA-regulated networks during muscle wasting in cardiac cachexia
Source: Sci Rep. 2017 Aug 1;7:6998. doi: 10.1038/s41598-017-07236-2 (PMC5539204; doi:10.1038/s41598-017-07236-2)
Supplement: Supplementary file 1 — Supplementary Information [file 41598_2017_7236_MOESM1_ESM.pdf]

Supplementary files

Integration of miRNA and mRNA expression profiles reveals microRNA-regulated networks during muscle wasting in cardiac cachexia

Leonardo N. Moraes[1], Geysson J. Fernandez[1], Ivan J. Vechetti-Júnior[1], Paula P. Freire[1], Rodrigo W. A. Souza[1], Rolando A. R. Villacis[2], Silvia R. Rogatto[2,3,4], Patricia P. Reis[3], Maeli Dal-Pai-Silva[1], and Robson F. Carvalho[1,*]

[1]Department of Morphology

Institute of Biosciences, São Paulo State University (UNESP)

Botucatu, SP, Brazil

[2]International Center of Research and Teaching (CIPE), Antônio Prudente Foundation, São Paulo, SP, Brazil

[3]Faculty of Medicine, São Paulo State University (UNESP), Brazil

Botucatu, SP, Brazil.

[4]Department of Clinical Cancer Genetics, Vejle Hospital, University of Odense, Odense, Denmark

*rcarvalho@ibb.unesp.br

Table Supplementary 1 mRNA differentially expressed in cardiac cachexia

Gene Symbol	Description	Ensembl ID	fold change log2	p value	fold change
Cyp2e1	cytochrome P450, family 2, subfamily e, polypeptide 1	ENSRNOG00000012458	5.63	0.0010	49.57
Mt1m	metallothionein 1M	ENSRNOG00000028841	3.03	0.0082	8.18
Fah	fumarylacetoacetate hydrolase	ENSRNOG00000013223	2.95	0.0023	7.74
Mt1	metallothionein 1	ENSRNOG00000025764	2.89	0.0042	7.42
Mt1	metallothionein 1	ENSRNOG00000038047	2.84	0.0037	7.17
Pbld1	phenazine biosynthesis-like protein domain containing 1	ENSRNOG00000000386	2.80	0.0061	6.99
Cdkn1a	cyclin-dependent kinase inhibitor 1A	ENSRNOG00000000521	2.65	0.0131	6.29
Serpine1	serpin peptidase inhibitor, clade E (nexin, plasminogen activator inhibitor type 1), member 1	ENSRNOG00000001414	2.46	0.0172	5.51
Mt2A	metallothionein 2A	ENSRNOG00000043098	2.41	0.0067	5.31
Lox	lysyl oxidase	ENSRNOG00000014426	2.28	0.0197	4.86
Slc7a8	solute carrier family 7 (amino acid transporter light chain, L system), member 8	ENSRNOG00000014311	2.17	0.0055	4.51
Stc1	stanniocalcin 1	ENSRNOG00000015075	2.04	0.0019	4.12
Acer2	alkaline ceramidase 2	ENSRNOG00000007637	2.04	0.0025	4.10
Galnt15	polypeptide N-acetylgalactosaminyltransferase 15	ENSRNOG00000019718	2.01	0.0028	4.04
Sdc4	syndecan 4	ENSRNOG00000014297	1.98	0.0023	3.96
Fam49a	family with sequence similarity 49, member A	ENSRNOG00000005836	1.98	0.0342	3.94
Tmem140	transmembrane protein 140	ENSRNOG00000026965	1.96	0.0080	3.89
Pnpla7	patatin-like phospholipase domain-containing protein 7-like	ENSRNOG00000008190	1.94	0.0039	3.83
Arntl	aryl hydrocarbon receptor nuclear translocator-like	ENSRNOG00000014448	1.93	0.0142	3.82
Alas2	aminolevulinate, delta-, synthase 2	ENSRNOG00000000167	1.93	0.0420	3.81
Fkbp5	FK506 binding protein 5	ENSRNOG00000022523	1.87	0.0023	3.65
Apold1	apolipoprotein L domain containing 1	ENSRNOG00000007830	1.87	0.0043	3.65
Hpd	4-hydroxyphenylpyruvate dioxygenase	ENSRNOG00000001338	1.86	0.0267	3.64
Sat1	spermidine/spermine N1-acetyl transferase 1	ENSRNOG00000003809	1.85	0.0066	3.62

Gene Symbol	Description	Ensembl ID	fold change log2	p value	fold change
Adamts9	ADAM metallopeptidase with thrombospondin type 1 motif, 9	ENSRNOG00000023257	1.85	0.0023	3.60
Ctsl	cathepsin L	ENSRNOG00000018566	1.84	0.0017	3.59
Mical2	microtubule associated monooxygenase, calponin and LIM domain containing 2	ENSRNOG00000016244	1.82	0.0100	3.54
Nfil3	nuclear factor, interleukin 3 regulated	ENSRNOG00000011668	1.81	0.0072	3.51
Eif4ebp1	eukaryotic translation initiation factor 4E binding protein 1	ENSRNOG00000012582	1.80	0.0023	3.49
Igfbp3	insulin-like growth factor binding protein 3	ENSRNOG00000008645	1.80	0.0030	3.49
Tgif1	TGFB-induced factor homeobox 1	ENSRNOG00000015906	1.80	0.0021	3.49
Tmem252	transmembrane protein 252	ENSRNOG00000025476	1.79	0.0054	3.47
Zfand2a	zinc finger, AN1-type domain 2A	ENSRNOG00000032917	1.78	0.0096	3.44
Gabarapl1	GABA(A) receptor-associated protein like 1	ENSRNOG00000008498	1.74	0.0023	3.33
Porf1	preoptic regulatory factor 1	ENSRNOG00000003891	1.73	0.0432	3.31
Spsb1	splA/ryanodine receptor domain and SOCS box containing 1	ENSRNOG00000017212	1.70	0.0142	3.26
Tp53inp1	tumor protein p53 inducible nuclear protein 1	ENSRNOG00000007964	1.69	0.0118	3.22
Ampd3	adenosine monophosphate deaminase 3	ENSRNOG00000018262	1.66	0.0042	3.17
Fam107a	family with sequence similarity 107, member A	ENSRNOG00000033261	1.66	0.0019	3.16
Nr4a1	nuclear receptor subfamily 4, group A, member 1	ENSRNOG00000007607	1.66	0.0240	3.15
Bcl2l1-ps1	Bcl2-like 1, pseudogene 1	ENSRNOG00000018503	1.66	0.0062	3.15
Abhd2	abhydrolase domain containing 2	ENSRNOG00000017120	1.64	0.0151	3.12
RGD621098	similar to RIKEN cDNA D230025D16Rik	ENSRNOG00000014668	1.64	0.0077	3.12
Sgk1	serum/glucocorticoid regulated kinase 1	ENSRNOG00000011815	1.63	0.0035	3.10
Slc4a1	solute carrier family 4 (anion exchanger), member 1	ENSRNOG00000020951	1.62	0.0154	3.08
Gadd45b	growth arrest and DNA-damage-inducible, beta	ENSRNOG00000019822	1.60	0.0082	3.04
Uck2	uridine-cytidine kinase 2	ENSRNOG00000003917	1.59	0.0363	3.02
Atp13a3	ATPase type 13A3	ENSRNOG00000001724	1.58	0.0257	3.00
Dapk1	death associated protein kinase 1	ENSRNOG00000018198	1.58	0.0142	2.99

Gene Symbol	Description	Ensembl ID	fold change log2	p value	fold change
Cpm	carboxypeptidase M	ENSRNOG00000034134	1.58	0.0249	2.98
Id1	inhibitor of DNA binding 1	ENSRNOG00000021750	1.57	0.0066	2.97
Gstm2	glutathione S-transferase mu 2	ENSRNOG00000019094	1.57	0.0436	2.96
Lgals3	lectin, galactoside-binding, soluble, 3	ENSRNOG00000010645	1.57	0.0214	2.96
Edn1	endothelin 1	ENSRNOG00000014361	1.56	0.0027	2.96
Btg2	BTG family, member 2	ENSRNOG00000003300	1.56	0.0030	2.95
Golm1	golgi membrane protein 1	ENSRNOG00000018400	1.56	0.0023	2.95
Tmem63c	transmembrane protein 63c	ENSRNOG00000011334	1.56	0.0163	2.94
Fgl2	fibrinogen-like 2	ENSRNOG00000012881	1.55	0.0056	2.94
Ahnak2	AHNAK nucleoprotein 2	ENSRNOG00000028545	1.55	0.0238	2.93
Sqrdl	sulfide quinone reductase-like (yeast)	ENSRNOG00000000172	1.53	0.0018	2.90
Rorc	RAR-related orphan receptor C	ENSRNOG00000020836	1.53	0.0082	2.89
Sik1	salt-inducible kinase 1	ENSRNOG00000001189	1.53	0.0179	2.88
Slc44a3	solute carrier family 44, member 3	ENSRNOG00000011723	1.53	0.0162	2.88
Arnt2	aryl hydrocarbon receptor nuclear translocator 2	ENSRNOG00000013017	1.52	0.0105	2.86
Oas1k	2 ' -5 ' oligoadenylate synthetase 1K	ENSRNOG00000033220	1.51	0.0142	2.86
Tcp11l2	t-complex 11, testis-specific-like 2	ENSRNOG00000007587	1.51	0.0053	2.85
Oser1	oxidative stress responsive serine-rich 1	ENSRNOG00000008297	1.51	0.0055	2.85
Nos1	nitric oxide synthase 1, neuronal	ENSRNOG00000001130	1.50	0.0434	2.83
Vwf	von Willebrand factor	ENSRNOG00000019689	1.49	0.0061	2.81
Ndrg1	N-myc downstream regulated 1	ENSRNOG00000007393	1.49	0.0035	2.81
Abra	actin-binding Rho activating protein	ENSRNOG00000007999	1.46	0.0088	2.76
Nek10	NIMA-related kinase 10	ENSRNOG00000005883	1.46	0.0450	2.75
Gpr157	G protein-coupled receptor 157	ENSRNOG00000017528	1.46	0.0042	2.74
Csrnp1	cysteine-serine-rich nuclear protein 1	ENSRNOG00000033433	1.45	0.0021	2.73

Gene Symbol	Description	Ensembl ID	fold change log2	p value	fold change
Clec2d	C-type lectin domain family 2 member D-like 1	ENSRNOG00000007866	1.44	0.0197	2.71
Rbm3	RNA binding motif (RNP1, RRM) protein 3	ENSRNOG00000005387	1.44	0.0023	2.71
Bcl2l1	Bcl2-like 1	ENSRNOG00000007946	1.44	0.0105	2.70
Pi4k2a	phosphatidylinositol 4-kinase type 2 alpha	ENSRNOG00000014675	1.44	0.0034	2.70
Rps27	ribosomal protein S27	ENSRNOG00000016961	1.41	0.0182	2.67
Myc	myelocytomatosis oncogene	ENSRNOG00000004500	1.41	0.0023	2.66
LOC689064	beta globin minor gene	ENSRNOG00000031230	1.40	0.0381	2.65
Cblb	Cbl proto-oncogene B, E3 ubiquitin protein ligase	ENSRNOG00000001982	1.40	0.0156	2.64
Foxo1	forkhead box O1	ENSRNOG00000013397	1.40	0.0154	2.64
Fam214a	family with sequence similarity 214, member A	ENSRNOG00000008150	1.39	0.0248	2.63
Pdk4	pyruvate dehydrogenase kinase, isozyme 4	ENSRNOG00000009565	1.39	0.0096	2.61
Ell	elongation factor RNA polymerase II	ENSRNOG00000019824	1.38	0.0090	2.61
Arid5a	AT rich interactive domain 5A (Mrf1 like)	ENSRNOG00000015382	1.38	0.0441	2.61
Sh3rf2	SH3 domain containing ring finger 2	ENSRNOG00000018780	1.38	0.0178	2.60
Ednrb	endothelin receptor type B	ENSRNOG00000010997	1.37	0.0319	2.58
Junb	jun B proto-oncogene	ENSRNOG00000042838	1.35	0.0224	2.55
Mreg	melanoregulin	ENSRNOG00000015774	1.34	0.0180	2.54
Osmr	oncostatin M receptor	ENSRNOG00000033192	1.33	0.0047	2.51
Gnrh1	gonadotropin-releasing hormone 1 (luteinizing-releasing hormone)	ENSRNOG00000013441	1.32	0.0498	2.49
Lgals5	lectin, galactose binding, soluble 5	ENSRNOG00000012557	1.32	0.0209	2.49
Cds2	CDP-diacylglycerol synthase (phosphatidate cytidylyltransferase) 2	ENSRNOG00000021265	1.31	0.0077	2.49
Ppp1r15a	protein phosphatase 1, regulatory subunit 15A	ENSRNOG00000020938	1.31	0.0045	2.48
Map3k6	mitogen-activated protein kinase kinase kinase 6	ENSRNOG00000008936	1.31	0.0133	2.48
Phlda1	pleckstrin homology-like domain, family A, member 1	ENSRNOG00000004019	1.30	0.0379	2.45
Ngp	neutrophilic granule protein	ENSRNOG00000024330	1.29	0.0088	2.45

Gene Symbol	Description	Ensembl ID	fold change log2	p value	fold change
Gpr56	G protein-coupled receptor 56	ENSRNOG00000014963	1.29	0.0043	2.45
Slc30a2	solute carrier family 30 (zinc transporter), member 2	ENSRNOG00000016650	1.29	0.0131	2.45
Bmx	BMX non-receptor tyrosine kinase	ENSRNOG00000003705	1.29	0.0043	2.44
Pik3c2a	phosphatidylinositol-4-phosphate 3-kinase, catalytic subunit type 2 alpha	ENSRNOG00000020479	1.28	0.0068	2.42
Map1lc3b	microtubule-associated protein 1 light chain 3 beta-like	ENSRNOG00000017905	1.27	0.0047	2.42
Colq	collagen-like tail subunit (single strand of homotrimer) of asymmetric acetylcholinesterase	ENSRNOG00000019615	1.27	0.0047	2.42
Ogfrl1	opioid growth factor receptor-like 1	ENSRNOG00000014142	1.27	0.0370	2.41
Per1	period circadian clock 1	ENSRNOG00000007387	1.26	0.0113	2.40
Mafk	v-maf avian musculoaponeurotic fibrosarcoma oncogene homolog K	ENSRNOG00000001277	1.26	0.0038	2.39
Unc5b	unc-5 homolog B (C. elegans)	ENSRNOG00000000567	1.25	0.0027	2.38
Tspan5	tetraspanin 5	ENSRNOG00000015913	1.25	0.0037	2.38
Ciart	circadian associated repressor of transcription	ENSRNOG00000042717	1.24	0.0376	2.37
Slco2a1	solute carrier organic anion transporter family, member 2a1	ENSRNOG00000009005	1.24	0.0354	2.36
Cdo1	cysteine dioxygenase type 1	ENSRNOG00000000158	1.22	0.0495	2.34
Fos	FBJ osteosarcoma oncogene	ENSRNOG00000008015	1.22	0.0220	2.33
Slc25a33	solute carrier family 25 (pyrimidine nucleotide carrier), member 33	ENSRNOG00000016949	1.22	0.0118	2.33
Ube2v1	ubiquitin-conjugating enzyme E2 variant 1	ENSRNOG00000025580	1.21	0.0155	2.32
Slc7a6	solute carrier family 7 (amino acid transporter light chain, y+L system), member 6	ENSRNOG00000019943	1.21	0.0030	2.32
Rhbdf2	rhomboid 5 homolog 2 (Drosophila)	ENSRNOG00000011459	1.21	0.0299	2.31
Cep85l	centrosomal protein 85-like	ENSRNOG00000000414	1.20	0.0360	2.29
Plat	plasminogen activator, tissue	ENSRNOG00000019018	1.20	0.0149	2.29
Gpihbp1	glycosylphosphatidylinositol anchored high density lipoprotein binding protein 1	ENSRNOG00000007475	1.20	0.0179	2.29
Hmox1	heme oxygenase (decycling) 1	ENSRNOG00000014117	1.19	0.0278	2.28
Cd24	CD24 molecule	ENSRNOG00000000321	1.19	0.0318	2.28

Gene Symbol	Description	Ensembl ID	fold change log2	p value	fold change
Ninj1	ninjurin 1	ENSRNOG00000016587	1.19	0.0411	2.28
LOC100910104	xin actin-binding repeat-containing protein 1-like	ENSRNOG00000037085	1.18	0.0054	2.27
Tmco4	transmembrane and coiled-coil domains 4	ENSRNOG00000017401	1.17	0.0136	2.26
Rpl22l1	ribosomal protein L22 like 1	ENSRNOG00000011817	1.17	0.0113	2.25
Ube4b	ubiquitination factor E4B	ENSRNOG00000014986	1.17	0.0179	2.24
Tinagl1	tubulointerstitial nephritis antigen-like 1	ENSRNOG00000013179	1.16	0.0027	2.23
Otulin	OTU deubiquitinase with linear linkage specificity	ENSRNOG00000012017	1.16	0.0216	2.23
PVR	poliovirus receptor	ENSRNOG00000019202	1.15	0.0342	2.21
Klf15	Kruppel-like factor 15	ENSRNOG00000017808	1.14	0.0183	2.21
Il18	interleukin 18	ENSRNOG00000009848	1.14	0.0434	2.21
Rpl22l2	ribosomal protein L22-like 2	ENSRNOG00000029173	1.14	0.0066	2.20
LOC685727	similar to neuron navigator 3	ENSRNOG00000042773	1.13	0.0186	2.19
Atp6v1h	ATPase, H+ transporting, lysosomal V1 subunit H	ENSRNOG00000030862	1.13	0.0081	2.19
Ppan	peter pan homolog (Drosophila)	ENSRNOG00000020608	1.13	0.0104	2.19
Lss	lanosterol synthase (2,3-oxidosqualene-lanosterol cyclase)	ENSRNOG00000001267	1.13	0.0271	2.19
Rhbdd1	rhomboid domain containing 1	ENSRNOG00000014620	1.13	0.0220	2.18
Bach1	BTB and CNC homology 1, basic leucine zipper transcription factor 1	ENSRNOG00000001582	1.12	0.0155	2.18
Gpnmb	glycoprotein (transmembrane) nmb	ENSRNOG00000008816	1.12	0.0084	2.17
Tcea1	transcription elongation factor A (SII) 1	ENSRNOG00000022323	1.12	0.0096	2.17
Gnl3	guanine nucleotide binding protein-like 3 (nucleolar)	ENSRNOG00000028461	1.11	0.0068	2.16
Pgpep1l	pyroglutamyl-peptidase I-like	ENSRNOG00000014402	1.10	0.0063	2.15
Cirbp	cold inducible RNA binding protein	ENSRNOG00000015999	1.10	0.0089	2.15
Chi3l1	chitinase 3-like 1 (cartilage glycoprotein-39)	ENSRNOG00000003312	1.10	0.0457	2.14
Tnfrsf12a	tumor necrosis factor receptor superfamily, member 12a	ENSRNOG00000003546	1.09	0.0131	2.14
Hgs	hepatocyte growth factor-regulated tyrosine kinase substrate	ENSRNOG00000036696	1.09	0.0066	2.13

Gene Symbol	Description	Ensembl ID	fold change log2	p value	fold change
Enc1	ectodermal-neural cortex 1	ENSRNOG00000016541	1.09	0.0233	2.12
Pkmyt1	protein kinase, membrane associated tyrosine/threonine 1	ENSRNOG00000003657	1.09	0.0042	2.12
Spon2	spondin 2, extracellular matrix protein	ENSRNOG00000006033	1.08	0.0479	2.12
Cyr61	cysteine-rich, angiogenic inducer, 61	ENSRNOG00000014350	1.08	0.0281	2.12
Fam117b	family with sequence similarity 117, member B	ENSRNOG00000022066	1.08	0.0066	2.11
Pla1a	phospholipase A1 member A	ENSRNOG00000027252	1.08	0.0221	2.11
Runx1	runt-related transcription factor 1	ENSRNOG00000001704	1.07	0.0290	2.10
Sfmbt1	Scm-like with four mbt domains 1	ENSRNOG00000016645	1.07	0.0068	2.10
Pcna	proliferating cell nuclear antigen	ENSRNOG00000021264	1.07	0.0066	2.10
Tmem38b	transmembrane protein 38B	ENSRNOG00000028063	1.07	0.0151	2.10
Nupr1	nuclear protein, transcriptional regulator, 1	ENSRNOG00000019206	1.06	0.0184	2.09
Gnl2	guanine nucleotide binding protein-like 2 (nucleolar)	ENSRNOG00000009430	1.06	0.0246	2.09
Psmd8	proteasome (prosome, macropain) 26S subunit, non-ATPase, 8	ENSRNOG00000037580	1.06	0.0118	2.08
Usp28	ubiquitin specific peptidase 28	ENSRNOG00000007325	1.05	0.0381	2.07
Sesn1	sestrin 1	ENSRNOG00000000302	1.05	0.0291	2.07
Tbc1d15	TBC1 domain family, member 15	ENSRNOG00000003889	1.05	0.0178	2.07
Sox18	SRY (sex determining region Y)-box 18	ENSRNOG00000016248	1.05	0.0096	2.07
Gfpt2	glutamine-fructose-6-phosphate transaminase 2	ENSRNOG00000002810	1.04	0.0342	2.06
Glis3	GLIS family zinc finger 3	ENSRNOG00000014768	1.04	0.0286	2.06
Rnf139	ring finger protein 139	ENSRNOG00000008987	1.04	0.0066	2.06
LOC688922	similar to Insulin-induced gene 1 protein (INSIG-1)	ENSRNOG00000027445	1.04	0.0352	2.06
Mecom	MDS1 and EVI1 complex locus	ENSRNOG00000012645	1.03	0.0213	2.04
Zfp143	zinc finger protein 143	ENSRNOG00000010087	1.03	0.0252	2.04
Chn2	chimerin 2	ENSRNOG00000009411	1.03	0.0415	2.04
Adm	adrenomedullin	ENSRNOG00000027030	1.03	0.0184	2.04

Gene Symbol	Description	Ensembl ID	fold change log2	p value	fold change
Chka	choline kinase alpha	ENSRNOG00000016791	1.03	0.0261	2.04
Hey1	hes-related family bHLH transcription factor with YRPW motif 1	ENSRNOG00000011593	1.02	0.0051	2.03
Ubc	ubiquitin C	ENSRNOG00000028756	1.02	0.0127	2.03
Olr1	oxidized low density lipoprotein (lectin-like) receptor 1	ENSRNOG00000008375	1.01	0.0172	2.02
Bcl2l11	BCL2-like 11 (apoptosis facilitator)	ENSRNOG00000016551	1.01	0.0039	2.02
Klhl18	kelch-like family member 18	ENSRNOG00000020880	1.01	0.0238	2.02
Arl4a	ADP-ribosylation factor-like 4A	ENSRNOG00000004282	1.01	0.0101	2.02
Utp15	UTP15, U3 small nucleolar ribonucleoprotein, homolog (S. cerevisiae)	ENSRNOG00000016591	1.01	0.0128	2.01
Zbtb10	zinc finger and BTB domain containing 10	ENSRNOG00000011319	1.01	0.0077	2.01
Slc2a3	solute carrier family 2 (facilitated glucose transporter), member 3	ENSRNOG00000008376	1.01	0.0179	2.01
Cd63	Cd63 molecule	ENSRNOG00000007650	1.01	0.0149	2.01
Fam219a	family with sequence similarity 219, member A	ENSRNOG00000039559	1.00	0.0082	2.00
Taf1d	TATA box binding protein (Tbp)-associated factor, RNA polymerase I, D	ENSRNOG00000010921	1.00	0.0163	2.00
Tex10	testis expressed 10	ENSRNOG00000008618	1.00	0.0489	2.00
Sorbs1	sorbin and SH3 domain containing 1	ENSRNOG00000015658	1.00	0.0162	2.00
Acsl4	acyl-CoA synthetase long-chain family member 4	ENSRNOG00000019180	1.00	0.0209	2.00
H6pd	hexose-6-phosphate dehydrogenase (glucose 1-dehydrogenase)	ENSRNOG00000017523	1.00	0.0251	2.00
Wdr62	WD repeat domain 62	ENSRNOG00000020807	0.99	0.0253	1.99
Fbxo32	F-box protein 32	ENSRNOG00000006738	0.99	0.0120	1.99
Angpt2	angiopoietin 2	ENSRNOG00000016696	0.99	0.0292	1.99
Fnip2	folliculin interacting protein 2	ENSRNOG00000027833	0.99	0.0172	1.99
Dsg4	desmoglein 4	ENSRNOG00000022364	0.99	0.0481	1.99
Rbp7	retinol binding protein 7, cellular	ENSRNOG00000015850	0.99	0.0114	1.98
RGD1564428	similar to FLJ32921 protein	ENSRNOG00000018721	0.99	0.0088	1.98
Utp18	UTP18 small subunit (SSU) processome component homolog (yeast)	ENSRNOG00000002644	0.99	0.0072	1.98

Gene Symbol	Description	Ensembl ID	fold change log2	p value	fold change
Ckap4	cytoskeleton-associated protein 4	ENSRNOG00000008016	0.99	0.0178	1.98
Cp	ceruloplasmin (ferroxidase)	ENSRNOG00000011913	0.99	0.0337	1.98
Il4r	interleukin 4 receptor	ENSRNOG00000015441	0.98	0.0225	1.98
Chrnb3	cholinergic receptor, nicotinic, beta 3 (neuronal)	ENSRNOG00000012448	0.98	0.0071	1.98
H3f3b	H3 histone, family 3B	ENSRNOG00000006532	0.98	0.0083	1.98
Arhgap10	Rho GTPase activating protein 10	ENSRNOG00000013152	0.98	0.0233	1.98
Ubxn8	UBX domain protein 8	ENSRNOG00000015109	0.98	0.0233	1.97
Actr1b	ARP1 actin-related protein 1 homolog B, centractin beta (yeast)	ENSRNOG00000016789	0.98	0.0477	1.97
Mall	mal, T-cell differentiation protein-like	ENSRNOG00000015599	0.98	0.0150	1.97
Mmp14	matrix metallopeptidase 14 (membrane-inserted)	ENSRNOG00000010947	0.97	0.0360	1.96
Cir1	corepressor interacting with RBPJ, 1	ENSRNOG00000018719	0.97	0.0151	1.96
Ccl2	chemokine (C-C motif) ligand 2	ENSRNOG00000007159	0.97	0.0279	1.96
Psmd11	proteasome (prosome, macropain) 26S subunit, non-ATPase, 11	ENSRNOG00000005538	0.97	0.0187	1.96
Fv1	Friend virus susceptibility 1	ENSRNOG00000019132	0.97	0.0054	1.95
Slc7a5	solute carrier family 7 (amino acid transporter light chain, L system), member 5	ENSRNOG00000018824	0.96	0.0465	1.95
Pcnx	pecanex homolog (Drosophila)	ENSRNOG00000007459	0.96	0.0068	1.94
Klf4	Kruppel-like factor 4 (gut)	ENSRNOG00000016299	0.95	0.0178	1.94
S100a9	S100 calcium binding protein A9	ENSRNOG00000011483	0.95	0.0238	1.93
Lgmn	legumain	ENSRNOG00000007089	0.94	0.0297	1.92
Whamm	WAS protein homolog associated with actin, golgi membranes and microtubules	ENSRNOG00000028113	0.94	0.0179	1.92
Rad18	RAD18 homolog (S. cerevisiae)	ENSRNOG00000005907	0.94	0.0322	1.91
Adrm1	adhesion regulating molecule 1	ENSRNOG00000006991	0.94	0.0142	1.91
Psmc4	proteasome (prosome, macropain) 26S subunit, ATPase, 4	ENSRNOG00000018994	0.93	0.0230	1.91
Pnrc1	proline-rich nuclear receptor coactivator 1	ENSRNOG00000007793	0.93	0.0066	1.91
Arhgap28	Rho GTPase activating protein 28	ENSRNOG00000017065	0.93	0.0118	1.91

Gene Symbol	Description	Ensembl ID	fold change log2	p value	fold change
Litaf	lipopolysaccharide-induced TNF factor	ENSRNOG00000002520	0.93	0.0445	1.91
Dll4	delta-like 4 (Drosophila)	ENSRNOG00000014011	0.93	0.0067	1.91
Zfp36l2	zinc finger protein 36, C3H1 type-like 2-like	ENSRNOG00000005067	0.93	0.0159	1.91
Creb3	cAMP responsive element binding protein 3	ENSRNOG00000016452	0.93	0.0374	1.90
Riok3	RIO kinase 3	ENSRNOG00000023376	0.93	0.0084	1.90
Fam134b	family with sequence similarity 134, member B	ENSRNOG00000010589	0.93	0.0142	1.90
Kctd9	potassium channel tetramerization domain containing 9	ENSRNOG00000012951	0.92	0.0233	1.90
Creg1	cellular repressor of E1A-stimulated genes 1	ENSRNOG00000003291	0.92	0.0450	1.90
Ftl1	ferritin light chain 1	ENSRNOG00000020843	0.92	0.0072	1.89
Dido1	death inducer-obliterator 1	ENSRNOG00000009936	0.91	0.0215	1.88
Tsnaxip1	translin-associated factor X interacting protein 1	ENSRNOG00000018954	0.91	0.0294	1.88
Osbpl8	oxysterol binding protein-like 8	ENSRNOG00000026962	0.91	0.0338	1.87
Usp53	ubiquitin specific peptidase 53	ENSRNOG00000014660	0.90	0.0085	1.87
Smad7	SMAD family member 7	ENSRNOG00000018359	0.90	0.0394	1.87
Phip	pleckstrin homology domain interacting protein	ENSRNOG00000008652	0.90	0.0073	1.87
Pnpla2	patatin-like phospholipase domain containing 2	ENSRNOG00000018736	0.90	0.0133	1.87
Lrrc8c	leucine rich repeat containing 8 family, member C	ENSRNOG00000002122	0.90	0.0142	1.86
Agfg2	ArfGAP with FG repeats 2	ENSRNOG00000001404	0.90	0.0290	1.86
Rgs3	regulator of G-protein signaling 3	ENSRNOG00000024501	0.90	0.0132	1.86
Lrrc30	leucine rich repeat containing 30	ENSRNOG00000030389	0.89	0.0181	1.86
Srgap1	SLIT-ROBO Rho GTPase activating protein 1	ENSRNOG00000004603	0.89	0.0179	1.86
Adarb1	adenosine deaminase, RNA-specific, B1	ENSRNOG00000001227	0.89	0.0286	1.86
Ccnl1	cyclin L1	ENSRNOG00000011586	0.89	0.0292	1.86
Tbc1d17	TBC1 domain family, member 17	ENSRNOG00000020191	0.89	0.0271	1.86
Mustn1	musculoskeletal, embryonic nuclear protein 1	ENSRNOG00000017369	0.89	0.0275	1.86

Gene Symbol	Description	Ensembl ID	fold change log2	p value	fold change
Nedd9	neural precursor cell expressed, developmentally down-regulated 9	ENSRNOG00000014548	0.89	0.0209	1.85
Utp11l	UTP11-like, U3 small nucleolar ribonucleoprotein, (yeast)	ENSRNOG00000007174	0.89	0.0485	1.85
Bid	BH3 interacting domain death agonist	ENSRNOG00000012439	0.88	0.0238	1.85
Hoga1	4-hydroxy-2-oxoglutarate aldolase 1	ENSRNOG00000029501	0.88	0.0212	1.85
Klf10	Kruppel-like factor 10	ENSRNOG00000006118	0.88	0.0197	1.84
Ift57	intraflagellar transport 57	ENSRNOG00000001958	0.88	0.0329	1.84
Pcsk5	proprotein convertase subtilisin/kexin type 5	ENSRNOG00000012036	0.88	0.0209	1.84
Clec12b	C-type lectin domain family 12, member B	ENSRNOG00000037070	0.87	0.0367	1.83
Ginm1	glycoprotein integral membrane 1	ENSRNOG00000015239	0.87	0.0102	1.83
Lin54	lin-54 DREAM MuvB core complex component	ENSRNOG00000002203	0.87	0.0149	1.83
Pmm1	phosphomannomutase 1	ENSRNOG00000005358	0.87	0.0414	1.83
RGD1566239	similar to RIKEN cDNA 2810428I15	ENSRNOG00000019990	0.87	0.0090	1.82
Zdhhc18	zinc finger, DHHC-type containing 18	ENSRNOG00000007021	0.87	0.0475	1.82
Cmip	c-Maf-inducing protein	ENSRNOG00000013178	0.86	0.0224	1.82
Gcnt2	glucosaminyl (N-acetyl) transferase 2, I-branching enzyme	ENSRNOG00000023778	0.86	0.0101	1.82
Nfkbia	nuclear factor of kappa light polypeptide gene enhancer in B-cells inhibitor, alpha	ENSRNOG00000007390	0.86	0.0146	1.82
Dnajb9	DnaJ (Hsp40) homolog, subfamily B, member 9	ENSRNOG00000004006	0.86	0.0085	1.82
Pim3	Pim-3 proto-oncogene, serine/threonine kinase	ENSRNOG00000029698	0.86	0.0253	1.82
Ftl1l1	ferritin light chain 1-like 1	ENSRNOG00000034150	0.86	0.0113	1.82
Nob1	NIN1/RPN12 binding protein 1 homolog (S. cerevisiae)	ENSRNOG00000021890	0.86	0.0100	1.81
Pdlim3	PDZ and LIM domain 3	ENSRNOG00000012658	0.85	0.0398	1.81
Cyp4f4	cytochrome P450, family 4, subfamily f, polypeptide 4	ENSRNOG00000032895	0.85	0.0197	1.80
Zc3h12a	zinc finger CCCH type containing 12A	ENSRNOG00000009131	0.85	0.0130	1.80
LOC102548013	uncharacterized LOC102548013	ENSRNOG00000023089	0.85	0.0122	1.80
Furin	furin (paired basic amino acid cleaving enzyme)	ENSRNOG00000011352	0.85	0.0151	1.80

Gene Symbol	Description	Ensembl ID	fold change log2	p value	fold change
Sprydd7	SPRY domain containing 7	ENSRNOG00000015095	0.84	0.0125	1.79
Mtss1	metastasis suppressor 1	ENSRNOG00000009001	0.84	0.0473	1.79
Gtpbp4	GTP binding protein 4	ENSRNOG00000016217	0.84	0.0133	1.79
Eif1	eukaryotic translation initiation factor 1	ENSRNOG00000033765	0.84	0.0238	1.79
Pappa	pregnancy-associated plasma protein A	ENSRNOG00000033527	0.83	0.0246	1.78
Nrarp	Notch-regulated ankyrin repeat protein	ENSRNOG00000009354	0.83	0.0092	1.78
Kpna1	karyopherin alpha 1	ENSRNOG00000000399	0.83	0.0432	1.78
Gpr4	G protein-coupled receptor 4	ENSRNOG00000016362	0.83	0.0494	1.78
Fam102b	family with sequence similarity 102, member B	ENSRNOG00000027540	0.83	0.0323	1.78
LOC499746	similar to hypothetical gene supported by AK097565; BC033939	ENSRNOG00000010002	0.83	0.0178	1.78
Npap60	nuclear pore associated protein	ENSRNOG00000013423	0.83	0.0342	1.77
Ftl1	ferritin light chain 1-like	ENSRNOG00000031506	0.82	0.0107	1.77
Efna1	ephrin A1	ENSRNOG00000020573	0.82	0.0433	1.77
Tgfbi	transforming growth factor, beta induced	ENSRNOG00000012216	0.82	0.0227	1.76
Terf2ip	telomeric repeat binding factor 2, interacting protein	ENSRNOG00000010712	0.82	0.0459	1.76
Rufy1	RUN and FYVE domain containing 1	ENSRNOG00000003536	0.82	0.0209	1.76
Maff	v-maf avian musculoaponeurotic fibrosarcoma oncogene homolog F	ENSRNOG00000012886	0.81	0.0415	1.76
Psmc6	proteasome (prosome, macropain) 26S subunit, ATPase, 6	ENSRNOG00000007203	0.81	0.0207	1.76
Igf1r	insulin-like growth factor 1 receptor	ENSRNOG00000014187	0.81	0.0135	1.76
Osbpl11	oxysterol binding protein-like 11	ENSRNOG00000001782	0.81	0.0467	1.76
Wsb1	WD repeat and SOCS box-containing 1	ENSRNOG00000012929	0.81	0.0223	1.75
Fhl2	four and a half LIM domains 2	ENSRNOG00000016866	0.81	0.0276	1.75
LOC691033	similar to GTPase activating protein testicular GAP1	ENSRNOG00000042535	0.80	0.0397	1.75
Pim1	Pim-1 proto-oncogene, serine/threonine kinase	ENSRNOG00000000529	0.80	0.0405	1.75
Ptprb	protein tyrosine phosphatase, receptor type, B	ENSRNOG00000004840	0.80	0.0147	1.74

Gene Symbol	Description	Ensembl ID	fold change log2	p value	fold change
Nubpl	nucleotide binding protein-like	ENSRNOG00000027444	0.80	0.0280	1.74
Slc25a2	solute carrier family 25 (mitochondrial carrier, ornithine transporter) member 2	ENSRNOG00000027359	0.80	0.0238	1.74
Ifngr1	interferon gamma receptor 1	ENSRNOG00000012074	0.80	0.0144	1.74
Capn8	calpain 8	ENSRNOG00000003468	0.80	0.0264	1.74
Myd88	myeloid differentiation primary response 88	ENSRNOG00000013634	0.80	0.0363	1.74
Notch4	notch 4	ENSRNOG00000000442	0.80	0.0100	1.74
LOC100363177	ferritin light chain 1-like	ENSRNOG00000039298	0.80	0.0155	1.74
Psmc1	proteasome (prosome, macropain) 26S subunit, ATPase, 1	ENSRNOG00000003951	0.80	0.0273	1.74
Mmp8	matrix metallopeptidase 8	ENSRNOG00000009907	0.79	0.0102	1.73
Haus6	HAUS augmin-like complex, subunit 6	ENSRNOG00000024854	0.79	0.0352	1.73
Itga5	integrin, alpha 5 (fibronectin receptor, alpha polypeptide)	ENSRNOG00000036832	0.79	0.0301	1.73
Ftsj3	FtsJ homolog 3 (E. coli)	ENSRNOG00000009857	0.79	0.0211	1.73
Slc28a2	solute carrier family 28 (concentrative nucleoside transporter), member 2	ENSRNOG00000028668	0.79	0.0216	1.73
Naa25	N(alpha)-acetyltransferase 25, NatB auxiliary subunit	ENSRNOG00000001350	0.79	0.0170	1.73
Slc2a1	solute carrier family 2 (facilitated glucose transporter), member 1	ENSRNOG00000007284	0.79	0.0102	1.72
Srfbp1	serum response factor binding protein 1	ENSRNOG00000014808	0.79	0.0175	1.72
Rbm20	RNA binding motif protein 20	ENSRNOG00000014705	0.78	0.0202	1.72
Tpsab1	tryptase alpha/beta 1	ENSRNOG00000024181	0.78	0.0446	1.72
Pi4k2b	phosphatidylinositol 4-kinase type 2 beta	ENSRNOG00000003924	0.78	0.0342	1.72
Vom2r11	vomeronasal 2 receptor, 11	ENSRNOG00000033775	0.78	0.0353	1.71
Ubxn4	UBX domain protein 4	ENSRNOG00000003625	0.78	0.0323	1.71
Wdr43	WD repeat domain 43	ENSRNOG00000026316	0.78	0.0105	1.71
Ankhd1	ankyrin repeat and KH domain containing 1	ENSRNOG00000030247	0.78	0.0173	1.71
Lztfl1	leucine zipper transcription factor-like 1	ENSRNOG00000006244	0.77	0.0179	1.71
Csrp2	cysteine and glycine-rich protein 2	ENSRNOG00000003772	0.77	0.0294	1.71

Gene Symbol	Description	Ensembl ID	fold change log2	p value	fold change
Ppp4r1	protein phosphatase 4, regulatory subunit 1	ENSRNOG00000013733	0.77	0.0278	1.71
Tbc1d30	TBC1 domain family, member 30	ENSRNOG00000023951	0.77	0.0284	1.71
Kctd20	potassium channel tetramerization domain containing 20	ENSRNOG00000000517	0.77	0.0398	1.71
Dmbt1	deleted in malignant brain tumors 1	ENSRNOG00000020560	0.77	0.0350	1.70
Pak1ip1	PAK1 interacting protein 1	ENSRNOG00000023799	0.77	0.0142	1.70
Adamts1	ADAM metallopeptidase with thrombospondin type 1 motif, 1	ENSRNOG00000001607	0.76	0.0172	1.70
Efcab2	EF-hand calcium binding domain 2	ENSRNOG00000042201	0.76	0.0155	1.70
Kcne4	potassium voltage-gated channel subfamily E member 4	ENSRNOG00000015039	0.76	0.0479	1.70
Uap1	UDP-N-acteylglucosamine pyrophosphorylase 1	ENSRNOG00000002926	0.76	0.0286	1.69
Dnttip1	deoxynucleotidyltransferase, terminal, interacting protein 1	ENSRNOG00000014933	0.76	0.0225	1.69
RGD1562608	similar to KIAA1328 protein	ENSRNOG00000029466	0.76	0.0180	1.69
Fmo2	flavin containing monooxygenase 2	ENSRNOG00000003510	0.76	0.0273	1.69
Lmbr1l	limb region 1 like	ENSRNOG00000033667	0.75	0.0249	1.69
Clec2e	C-type lectin domain family 2, member E	ENSRNOG00000037073	0.75	0.0209	1.68
Fbxo30	F-box protein 30	ENSRNOG00000014852	0.75	0.0109	1.68
Dhx37	DEAH (Asp-Glu-Ala-His) box polypeptide 37	ENSRNOG00000022171	0.75	0.0315	1.68
Igf2bp2	insulin-like growth factor 2 mRNA binding protein 2	ENSRNOG00000025946	0.75	0.0342	1.68
Crebrf	CREB3 regulatory factor	ENSRNOG00000020769	0.75	0.0463	1.68
Mybbp1a	MYB binding protein (P160) 1a	ENSRNOG00000015236	0.75	0.0101	1.68
Dot1l	DOT1-like histone H3K79 methyltransferase	ENSRNOG00000032546	0.75	0.0139	1.68
Nufip1	nuclear fragile X mental retardation protein interacting protein 1	ENSRNOG00000001033	0.75	0.0163	1.68
Eps8	epidermal growth factor receptor pathway substrate 8	ENSRNOG00000007047	0.74	0.0405	1.67
Id3	inhibitor of DNA binding 3	ENSRNOG00000026124	0.74	0.0169	1.67
Tfdp2	transcription factor Dp-2 (E2F dimerization partner 2)	ENSRNOG00000011241	0.74	0.0219	1.67
Rnasel	ribonuclease L (2',5'-oligoisoadenylate synthetase-dependent)	ENSRNOG00000027017	0.74	0.0101	1.67

Gene Symbol	Description	Ensembl ID	fold change log2	p value	fold change
Nfx1	nuclear transcription factor, X-box binding 1	ENSRNOG00000009015	0.74	0.0264	1.67
Tcerg1	transcription elongation regulator 1	ENSRNOG00000018849	0.74	0.0155	1.67
Acaca	acetyl-CoA carboxylase alpha	ENSRNOG00000034013	0.74	0.0417	1.67
Kdm3a	lysine (K)-specific demethylase 3A	ENSRNOG00000007814	0.74	0.0118	1.67
Nbr1	similar to neighbor of Brca1 gene 1	ENSRNOG00000020730	0.74	0.0497	1.67
Art3	ADP-ribosyltransferase 3	ENSRNOG00000002256	0.74	0.0284	1.67
Ddrgk1	DDRGK domain containing 1	ENSRNOG00000021232	0.73	0.0101	1.66
Exosc8	exosome component 8	ENSRNOG00000013434	0.73	0.0173	1.66
RGD1565498	similar to Hypothetical protein LOC270802	ENSRNOG00000028782	0.73	0.0153	1.66
Ccdc109b	coiled-coil domain containing 109B	ENSRNOG00000009433	0.73	0.0445	1.66
Zfand5	zinc finger, AN1-type domain 5	ENSRNOG00000018107	0.73	0.0139	1.66
Ranbp10	RAN binding protein 10	ENSRNOG00000018000	0.73	0.0113	1.66
Lrp2bp	Lrp2 binding protein	ENSRNOG00000011178	0.73	0.0194	1.65
Gata6	GATA binding protein 6	ENSRNOG00000023433	0.73	0.0286	1.65
S1pr1	sphingosine-1-phosphate receptor 1	ENSRNOG00000013683	0.73	0.0184	1.65
Nploc4	nuclear protein localization 4 homolog (S. cerevisiae)	ENSRNOG00000036698	0.72	0.0225	1.65
Gmeb2	glucocorticoid modulatory element binding protein 2	ENSRNOG00000013339	0.72	0.0215	1.65
Sap30	Sin3A-associated protein	ENSRNOG00000013218	0.72	0.0182	1.65
Nfkbiz	nuclear factor of kappa light polypeptide gene enhancer in B-cells inhibitor, zeta	ENSRNOG00000031163	0.72	0.0273	1.65
Pabpc1	poly(A) binding protein, cytoplasmic 1	ENSRNOG00000008639	0.72	0.0325	1.65
Tenc1	tensin like C1 domain containing phosphatase (tensin 2)	ENSRNOG00000010588	0.72	0.0123	1.64
Aup1	ancient ubiquitous protein 1	ENSRNOG00000007842	0.72	0.0123	1.64
Klf11	Kruppel-like factor 11	ENSRNOG00000000216	0.71	0.0234	1.64
Cited2	Cbp/p300-interacting transactivator, with Glu/Asp-rich carboxy-terminal domain, 2	ENSRNOG00000012193	0.71	0.0365	1.64
Anp32b	acidic (leucine-rich) nuclear phosphoprotein 32 family, member B	ENSRNOG00000009266	0.71	0.0134	1.64

Gene Symbol	Description	Ensembl ID	fold change log2	p value	fold change
Fam91a1	family with sequence similarity 91, member A1	ENSRNOG00000008271	0.71	0.0471	1.64
Tspan14	tetraspanin 14	ENSRNOG00000010813	0.71	0.0485	1.64
Tnfrsf1b	tumor necrosis factor receptor superfamily, member 1b	ENSRNOG00000016575	0.71	0.0271	1.63
Fcho2	FCH domain only 2	ENSRNOG00000015334	0.71	0.0177	1.63
Adcyap1r1	adenylate cyclase activating polypeptide 1 receptor 1	ENSRNOG00000012098	0.71	0.0315	1.63
Psmd3	proteasome (prosome, macropain) 26S subunit, non-ATPase, 3	ENSRNOG00000028103	0.71	0.0243	1.63
Medag	mesenteric estrogen-dependent adipogenesis	ENSRNOG00000000906	0.71	0.0473	1.63
Atl3	atlastin GTPase 3	ENSRNOG00000021203	0.70	0.0296	1.63
Sema3g	sema domain, immunoglobulin domain (Ig), short basic domain, secreted, (semaphorin) 3G	ENSRNOG00000018952	0.70	0.0195	1.63
LOC691960	similar to solute carrier family 28, member 2	ENSRNOG00000018204	0.70	0.0178	1.63
Tmem179	transmembrane protein 179	ENSRNOG00000013128	0.70	0.0191	1.63
Tsr1	TSR1, 20S rRNA accumulation, homolog (S. cerevisiae)	ENSRNOG00000002980	0.70	0.0285	1.62
Rlf	rearranged L-myc fusion	ENSRNOG00000027921	0.70	0.0200	1.62
Gnai3	guanine nucleotide binding protein (G protein), alpha inhibiting activity polypeptide 3	ENSRNOG00000019465	0.70	0.0398	1.62
Dgkh	diacylglycerol kinase, eta	ENSRNOG00000010065	0.70	0.0146	1.62
Amotl2	angiomotin like 2	ENSRNOG00000008487	0.70	0.0134	1.62
Pcf11	PCF11 cleavage and polyadenylation factor subunit	ENSRNOG00000009891	0.69	0.0165	1.62
Timp3	TIMP metallopeptidase inhibitor 3	ENSRNOG00000004303	0.69	0.0450	1.61
Fry	furry homolog (Drosophila)	ENSRNOG00000000894	0.69	0.0178	1.61
Avpi1	arginine vasopressin-induced 1	ENSRNOG00000014828	0.69	0.0276	1.61
Esam	endothelial cell adhesion molecule	ENSRNOG00000033217	0.69	0.0125	1.61
Plxna2	plexin A2	ENSRNOG00000007324	0.69	0.0155	1.61
RGD1311458	similar to cDNA sequence BC027231; hypothetical protein MGC27931	ENSRNOG00000013721	0.69	0.0142	1.61
Rybp	RING1 and YY1 binding protein	ENSRNOG00000005353	0.69	0.0185	1.61
Ctsd	cathepsin D	ENSRNOG00000020206	0.68	0.0197	1.61

Gene Symbol	Description	Ensembl ID	fold change log2	p value	fold change
Psmd13	proteasome (prosome, macropain) 26S subunit, non-ATPase, 13	ENSRNOG00000014109	0.68	0.0360	1.60
Tpbg	trophoblast glycoprotein	ENSRNOG00000010694	0.68	0.0435	1.60
Txnl1	thioredoxin-like 1	ENSRNOG00000018818	0.68	0.0352	1.60
Brpf3	bromodomain and PHD finger containing, 3	ENSRNOG00000028641	0.68	0.0329	1.60
Cnppd1	cyclin Pas1/PHO80 domain containing 1	ENSRNOG00000018325	0.68	0.0125	1.60
Map3k2	mitogen activated protein kinase kinase kinase 2	ENSRNOG00000014089	0.68	0.0197	1.60
Nucb2	nucleobindin 2	ENSRNOG00000020456	0.67	0.0471	1.60
Lrrcc1	leucine rich repeat and coiled-coil centrosomal protein 1	ENSRNOG00000010891	0.67	0.0324	1.60
Tmem214	transmembrane protein 214	ENSRNOG00000008812	0.67	0.0281	1.60
Palmd	palmdelphin	ENSRNOG00000016508	0.67	0.0373	1.59
Grina	glutamate receptor, ionotropic, N-methyl D-aspartate-associated protein 1 (glutamate binding)	ENSRNOG00000029941	0.67	0.0184	1.59
Ing3	inhibitor of growth family, member 3	ENSRNOG00000005496	0.67	0.0320	1.59
Naca	nascent polypeptide-associated complex alpha subunit	ENSRNOG00000002632	0.67	0.0441	1.59
Vwa1	von Willebrand factor A domain containing 1	ENSRNOG00000018338	0.67	0.0327	1.59
Psma4	proteasome (prosome, macropain) subunit, alpha type 4	ENSRNOG00000013493	0.67	0.0293	1.59
Tra2b	transformer 2 beta homolog (Drosophila)	ENSRNOG00000001783	0.67	0.0273	1.59
Zswim4	zinc finger, SWIM-type containing 4	ENSRNOG00000007582	0.67	0.0375	1.59
Zfp110	zinc finger protein 110	ENSRNOG00000031328	0.67	0.0151	1.59
Med13l	mediator complex subunit 13-like	ENSRNOG00000001120	0.67	0.0350	1.59
Cnksr1	connector enhancer of kinase suppressor of Ras 1	ENSRNOG00000022838	0.67	0.0217	1.59
Med15	mediator complex subunit 15	ENSRNOG00000001877	0.67	0.0357	1.59
Myb	myeloblastosis oncogene	ENSRNOG00000014313	0.67	0.0360	1.59
Tmem171	transmembrane protein 171	ENSRNOG00000015449	0.66	0.0209	1.59
Lin37	lin-37 DREAM MuvB core complex component	ENSRNOG00000020929	0.66	0.0433	1.59
Psmd2	proteasome (prosome, macropain) 26S subunit, non-ATPase, 2	ENSRNOG00000001719	0.66	0.0142	1.58

Gene Symbol	Description	Ensembl ID	fold change log2	p value	fold change
Brms1	breast cancer metastasis-suppressor 1 homolog	ENSRNOG00000020117	0.66	0.0372	1.58
Pgf	placental growth factor	ENSRNOG00000005650	0.66	0.0213	1.58
Tor1aip1	torsin A interacting protein 1	ENSRNOG00000003946	0.66	0.0177	1.58
Mast4	microtubule associated serine/threonine kinase family member 4	ENSRNOG00000010720	0.66	0.0209	1.58
Hectd1	HECT domain containing E3 ubiquitin protein ligase 1	ENSRNOG00000006905	0.66	0.0412	1.58
Hsf4	heat shock transcription factor 4	ENSRNOG00000015253	0.66	0.0238	1.58
Smad6	SMAD family member 6	ENSRNOG00000009173	0.66	0.0295	1.58
Zfp598	zinc finger protein 598	ENSRNOG00000012434	0.66	0.0160	1.58
Rad54b	RAD54 homolog B (S. cerevisiae)	ENSRNOG00000039949	0.66	0.0462	1.58
Atf6	activating transcription factor 6	ENSRNOG00000024632	0.66	0.0495	1.58
Pdgfrb	platelet derived growth factor receptor, beta polypeptide	ENSRNOG00000018461	0.66	0.0284	1.58
Lonrf3	LON peptidase N-terminal domain and ring finger 3	ENSRNOG00000013092	0.66	0.0376	1.58
Trmt6	tRNA methyltransferase 6 homolog (S. cerevisiae)	ENSRNOG00000021270	0.66	0.0435	1.57
Ddx21	DEAD (Asp-Glu-Ala-Asp) box helicase 21	ENSRNOG00000043099	0.65	0.0265	1.57
Peak1	pseudopodium-enriched atypical kinase 1	ENSRNOG00000042519	0.65	0.0375	1.57
Egln3	egl-9 family hypoxia-inducible factor 3	ENSRNOG00000005053	0.65	0.0227	1.57
Rlim	ring finger protein, LIM domain interacting	ENSRNOG00000002824	0.65	0.0249	1.57
Lrrc32	leucine rich repeat containing 32	ENSRNOG00000015310	0.65	0.0433	1.57
Arhgap26	Rho GTPase activating protein 26	ENSRNOG00000013920	0.65	0.0166	1.57
Zrsr1	zinc finger (CCCH type), RNA binding motif and serine/arginine rich 1	ENSRNOG00000009639	0.65	0.0405	1.57
Cul4b	cullin 4B	ENSRNOG00000002585	0.65	0.0159	1.57
Atr	ATR serine/threonine kinase	ENSRNOG00000010027	0.65	0.0390	1.57
Il6st	interleukin 6 signal transducer	ENSRNOG00000013963	0.65	0.0273	1.57
Slc7a6os	solute carrier family 7, member 6 opposite strand	ENSRNOG00000020049	0.65	0.0343	1.57
Zfp868	zinc finger protein 868	ENSRNOG00000028919	0.65	0.0244	1.57

Gene Symbol	Description	Ensembl ID	fold change log2	p value	fold change
Ddx27	DEAD (Asp-Glu-Ala-Asp) box polypeptide 27	ENSRNOG00000008081	0.65	0.0160	1.57
Anapc7	anaphase promoting complex subunit 7	ENSRNOG00000001283	0.65	0.0369	1.56
Txnip	thioredoxin interacting protein	ENSRNOG00000021201	0.65	0.0489	1.56
Rbm28	RNA binding motif protein 28	ENSRNOG00000005468	0.64	0.0209	1.56
Slc20a2	solute carrier family 20 (phosphate transporter), member 2	ENSRNOG00000019490	0.64	0.0462	1.56
Rad17	RAD17 homolog (S. pombe)	ENSRNOG00000018353	0.64	0.0215	1.56
Sec14l1	SEC14-like 1 (S. cerevisiae)	ENSRNOG00000002722	0.64	0.0360	1.56
Cxcl11	chemokine (C-X-C motif) ligand 11	ENSRNOG00000022298	0.64	0.0441	1.56
Strc	stereocilin	ENSRNOG00000014845	0.64	0.0230	1.56
F2rl1	coagulation factor II (thrombin) receptor-like 1	ENSRNOG00000018003	0.64	0.0155	1.56
Psma3	proteasome subunit alpha type-3-like	ENSRNOG00000022652	0.64	0.0225	1.56
Lamp2	lysosomal-associated membrane protein 2	ENSRNOG00000000164	0.64	0.0310	1.56
Rpl24	ribosomal protein L24	ENSRNOG00000001611	0.64	0.0152	1.55
Pmaip1	phorbol-12-myristate-13-acetate-induced protein 1	ENSRNOG00000018770	0.64	0.0256	1.55
Nap1l1	nucleosome assembly protein 1-like 1	ENSRNOG00000003890	0.64	0.0209	1.55
Arhgap27	Rho GTPase activating protein 27	ENSRNOG00000028569	0.63	0.0353	1.55
Chrna1	cholinergic receptor, nicotinic, alpha 1 (muscle)	ENSRNOG00000018286	0.63	0.0178	1.55
Sfr1	SWI5-dependent recombination repair 1	ENSRNOG00000012641	0.63	0.0338	1.55
Pacsin3	protein kinase C and casein kinase substrate in neurons 3	ENSRNOG00000014204	0.63	0.0442	1.55
Hes1	hes family bHLH transcription factor 1	ENSRNOG00000001720	0.63	0.0293	1.55
Ezh1	enhancer of zeste 1 polycomb repressive complex 2 subunit	ENSRNOG00000020336	0.63	0.0296	1.55
Cpsf7	cleavage and polyadenylation specific factor 7	ENSRNOG00000020668	0.63	0.0360	1.55
Uaca	uveal autoantigen with coiled-coil domains and ankyrin repeats	ENSRNOG00000012868	0.62	0.0159	1.54
Phka2	phosphorylase kinase, alpha 2	ENSRNOG00000003949	0.62	0.0274	1.54
Pes1	pescadillo ribosomal biogenesis factor 1	ENSRNOG00000004515	0.62	0.0212	1.54

Gene Symbol	Description	Ensembl ID	fold change log2	p value	fold change
Npm1	nucleophosmin (nucleolar phosphoprotein B23, numatrin)	ENSRNOG00000004616	0.62	0.0405	1.54
Sec23b	Sec23 homolog B (S. cerevisiae)	ENSRNOG00000008411	0.62	0.0498	1.54
RGD1309350	5-hydroxyisourate hydrolase-like	ENSRNOG00000012744	0.62	0.0184	1.54
Cdc42se1	CDC42 small effector 1	ENSRNOG00000021112	0.62	0.0342	1.54
Brd8	bromodomain containing 8	ENSRNOG00000020340	0.62	0.0344	1.54
Clcf1	cardiotrophin-like cytokine factor 1	ENSRNOG00000018752	0.62	0.0451	1.54
Efnb2	ephrin B2	ENSRNOG00000014648	0.62	0.0230	1.53
LOC685391	LRRGT00063	ENSRNOG00000043170	0.62	0.0477	1.53
Psmd1	proteasome (prosome, macropain) 26S subunit, non-ATPase, 1	ENSRNOG00000017730	0.62	0.0337	1.53
Psmd12	proteasome (prosome, macropain) 26S subunit, non-ATPase, 12	ENSRNOG00000003117	0.62	0.0176	1.53
Taf1b	TATA box binding protein (Tbp)-associated factor, RNA polymerase I, B	ENSRNOG00000004506	0.61	0.0209	1.53
Snx10	sorting nexin 10	ENSRNOG00000011944	0.61	0.0500	1.53
Slc3a2	solute carrier family 3 (amino acid transporter heavy chain), member 2	ENSRNOG00000018487	0.61	0.0183	1.53
Dyrk1a	dual-specificity tyrosine-(Y)-phosphorylation regulated kinase 1A	ENSRNOG00000001662	0.61	0.0463	1.53
Dnajc3	DnaJ (Hsp40) homolog, subfamily C, member 3	ENSRNOG00000010352	0.61	0.0371	1.53
Btaf1	BTAF1 RNA polymerase II, B-TFIID transcription factor-associated, 170kDa	ENSRNOG00000017938	0.61	0.0216	1.53
Ncl	nucleolin	ENSRNOG00000018273	0.61	0.0184	1.53
Rhbdd2	rhomboid domain containing 2	ENSRNOG00000001443	0.61	0.0272	1.53
Hlx	H2.0-like homeobox	ENSRNOG00000002309	0.61	0.0241	1.53
Fbxo4	F-box protein 4	ENSRNOG00000015622	0.61	0.0296	1.53
Amn1	antagonist of mitotic exit network 1 homolog (S. cerevisiae)	ENSRNOG00000036917	0.61	0.0208	1.53
Wfdc2	WAP four-disulfide core domain 2	ENSRNOG00000014739	0.61	0.0469	1.53
Gstp1	glutathione S-transferase pi 1	ENSRNOG00000018237	0.61	0.0367	1.52
Galc	galactosylceramidase	ENSRNOG00000003759	0.61	0.0269	1.52
Ptpn2	protein tyrosine phosphatase, non-receptor type 2	ENSRNOG00000017453	0.61	0.0265	1.52

Gene Symbol	Description	Ensembl ID	fold change log2	p value	fold change
Kcnmb4	potassium large conductance calcium-activated channel, subfamily M, beta member 4	ENSRNOG00000022297	0.61	0.0300	1.52
Hip1r	huntingtin interacting protein 1 related	ENSRNOG00000001091	0.61	0.0239	1.52
Zfp259	zinc finger protein 259	ENSRNOG00000018481	0.61	0.0209	1.52
Mpdu1	mannose-P-dolichol utilization defect 1	ENSRNOG00000012162	0.61	0.0292	1.52
Lemd3	LEM domain containing 3	ENSRNOG00000024027	0.61	0.0322	1.52
Blm	Bloom syndrome, RecQ helicase-like	ENSRNOG00000011213	0.60	0.0186	1.52
Hnrnpf	heterogeneous nuclear ribonucleoprotein F	ENSRNOG00000014562	0.60	0.0307	1.52
Pqlc2	PQ loop repeat containing 2	ENSRNOG00000017706	0.60	0.0425	1.52
Psmd14	proteasome (prosome, macropain) 26S subunit, non-ATPase, 14	ENSRNOG00000004911	0.60	0.0479	1.52
Ppm1d	protein phosphatase, Mg2+/Mn2+ dependent, 1D	ENSRNOG00000003329	0.60	0.0202	1.52
Psma6	proteasome (prosome, macropain) subunit, alpha type 6	ENSRNOG00000007114	0.60	0.0253	1.52
Utp14a	UTP14, U3 small nucleolar ribonucleoprotein, homolog A (yeast)	ENSRNOG00000005012	0.60	0.0274	1.52
Mecp2	methyl CpG binding protein 2	ENSRNOG00000037262	0.60	0.0307	1.52
Pms2	postmeiotic segregation increased 2 (S. cerevisiae)	ENSRNOG00000001040	0.60	0.0363	1.51
Slc25a17	solute carrier family 25 (mitochondrial carrier, peroxisomal membrane protein), member 17	ENSRNOG00000018920	0.60	0.0197	1.51
Zfp496	zinc finger protein 496	ENSRNOG00000003129	0.60	0.0373	1.51
Hbp1	HMG-box transcription factor 1	ENSRNOG00000008927	0.60	0.0172	1.51
Cmtm8	CKLF-like MARVEL transmembrane domain containing 8	ENSRNOG00000011201	0.60	0.0342	1.51
Ewsr1	EWS RNA-binding protein 1	ENSRNOG00000009437	0.60	0.0380	1.51
RGD1306353	similar to Zinc finger protein 198	ENSRNOG00000029220	0.60	0.0393	1.51
Meis3	Meis homeobox 3	ENSRNOG00000021390	0.60	0.0272	1.51
Foxk1	forkhead box K1	ENSRNOG00000001104	0.59	0.0459	1.51
Pcgf5	polycomb group ring finger 5	ENSRNOG00000018532	0.59	0.0312	1.51
Eva1c	eva-1 homolog C	ENSRNOG00000002072	0.59	0.0341	1.51
Ppp1r13l	protein phosphatase 1, regulatory subunit 13 like	ENSRNOG00000025350	0.59	0.0374	1.51

Gene Symbol	Description	Ensembl ID	fold change log2	p value	fold change
Ktn1	kinectin 1 (kinesin receptor)	ENSRNOG00000012255	0.59	0.0387	1.51
Slc25a38	solute carrier family 25, member 38	ENSRNOG00000018552	0.59	0.0360	1.51
Tbc1d9	TBC1 domain family, member 9 (with GRAM domain)	ENSRNOG00000003496	0.59	0.0459	1.51
Usp16	ubiquitin specific peptidase 16	ENSRNOG00000001598	0.59	0.0401	1.51
Sqstm1	sequestosome 1	ENSRNOG00000003147	0.59	0.0296	1.51
Nr2c2	nuclear receptor subfamily 2, group C, member 2	ENSRNOG00000010536	0.59	0.0405	1.51
Psmb3	proteasome (prosome, macropain) subunit, beta type 3	ENSRNOG00000012938	0.59	0.0337	1.51
Bach2	BTB and CNC homology 1, basic leucine zipper transcription factor 2	ENSRNOG00000006170	0.59	0.0398	1.51
Zscan21	zinc finger and SCAN domain containing 21	ENSRNOG00000039234	0.59	0.0253	1.50
March7	membrane-associated ring finger (C3HC4) 7, E3 ubiquitin protein ligase	ENSRNOG00000006241	0.59	0.0445	1.50
Cul1	cullin 1	ENSRNOG00000005310	0.59	0.0258	1.50
Stk11ip	serine/threonine kinase 11 interacting protein	ENSRNOG00000020107	0.59	0.0186	1.50
Mettl9	methyltransferase like 9-like	ENSRNOG00000025940	0.59	0.0294	1.50
Lphn2	latrophilin 2	ENSRNOG00000032660	0.59	0.0459	1.50
Tspan2	tetraspanin 2	ENSRNOG00000023338	0.59	0.0323	1.50
Cers4	ceramide synthase 4	ENSRNOG00000001072	-0.59	0.0475	-1.50
Lama4	laminin, alpha 4	ENSRNOG00000000599	-0.59	0.0370	-1.50
Papln	papilin, proteoglycan-like sulfated glycoprotein	ENSRNOG00000009448	-0.59	0.0307	-1.50
Slc16a12	solute carrier family 16, member 12	ENSRNOG00000021916	-0.59	0.0494	-1.50
Ndufb6	NADH dehydrogenase (ubiquinone) 1 beta subcomplex, 6	ENSRNOG00000024539	-0.59	0.0312	-1.50
Ift27	intraflagellar transport 27	ENSRNOG00000006440	-0.59	0.0477	-1.50
Pqlc1	PQ loop repeat containing 1	ENSRNOG00000017204	-0.59	0.0225	-1.50
Wdr7	WD repeat domain 7	ENSRNOG00000018387	-0.59	0.0450	-1.50
Hook3	hook microtubule-tethering protein 3	ENSRNOG00000014275	-0.59	0.0391	-1.51
Tspan9	tetraspanin 9	ENSRNOG00000005442	-0.59	0.0255	-1.51

Gene Symbol	Description	Ensembl ID	fold change log2	p value	fold change
Extl2	exostosin-like glycosyltransferase 2	ENSRNOG00000014323	-0.59	0.0294	-1.51
Giot1	gonadotropin inducible ovarian transcription factor 1	ENSRNOG00000008417	-0.59	0.0405	-1.51
Ndufs5	rCG31129-like	ENSRNOG00000026646	-0.59	0.0251	-1.51
Pax7	paired box 7	ENSRNOG00000018739	-0.59	0.0198	-1.51
Atp5c1	ATP synthase, H+ transporting, mitochondrial F1 complex, gamma polypeptide 1	ENSRNOG00000019223	-0.59	0.0342	-1.51
Fgd1	FYVE, RhoGEF and PH domain containing 1	ENSRNOG00000038970	-0.59	0.0436	-1.51
Chst7	carbohydrate (N-acetylglucosamine 6-O) sulfotransferase 7	ENSRNOG00000004258	-0.59	0.0363	-1.51
Zfp518b	zinc finger protein 518B	ENSRNOG00000028534	-0.59	0.0180	-1.51
Idh3g	isocitrate dehydrogenase 3 (NAD), gamma	ENSRNOG00000037284	-0.59	0.0473	-1.51
Sdhd	succinate dehydrogenase complex, subunit D, integral membrane protein	ENSRNOG00000022980	-0.59	0.0342	-1.51
Tulp4	tubby like protein 4	ENSRNOG00000018012	-0.60	0.0203	-1.51
Col22a1	collagen, type XXII, alpha 1	ENSRNOG00000024824	-0.60	0.0281	-1.51
Pcnxl4	pecanex-like 4 (Drosophila)	ENSRNOG00000005568	-0.60	0.0390	-1.51
Gls2	glutaminase 2 (liver, mitochondrial)	ENSRNOG00000031612	-0.60	0.0218	-1.51
Zfp13	zinc finger protein 13	ENSRNOG00000003455	-0.60	0.0420	-1.51
Fam69b	family with sequence similarity 69, member B	ENSRNOG00000004532	-0.60	0.0435	-1.51
Gemin7	gem (nuclear organelle) associated protein 7	ENSRNOG00000034168	-0.60	0.0301	-1.51
Zfp553	zinc finger protein 553	ENSRNOG00000017837	-0.60	0.0369	-1.51
Tceal8	transcription elongation factor A (SII)-like 8	ENSRNOG00000028585	-0.60	0.0471	-1.51
Ube2d1	ubiquitin-conjugating enzyme E2D 1	ENSRNOG00000000611	-0.60	0.0405	-1.51
Rimbp2	RIMS binding protein 2	ENSRNOG00000022893	-0.60	0.0203	-1.51
Grin3a	glutamate receptor, ionotropic, N-methyl-D-aspartate 3A	ENSRNOG00000005723	-0.60	0.0493	-1.51
Grcc10	gene rich cluster, C10 gene	ENSRNOG00000025810	-0.60	0.0197	-1.52
Sh2b2	SH2B adaptor protein 2	ENSRNOG00000001425	-0.60	0.0360	-1.52
Adamts20	ADAM metallopeptidase with thrombospondin type 1 motif, 20	ENSRNOG00000033397	-0.60	0.0246	-1.52

Gene Symbol	Description	Ensembl ID	fold change log2	p value	fold change
LOC681458	similar to stearoyl-coenzyme A desaturase 3	ENSRNOG00000013279	-0.60	0.0486	-1.52
Fem1c	fem-1 homolog c (C. elegans)	ENSRNOG00000003578	-0.60	0.0342	-1.52
Isoc1	isochorismatase domain containing 1	ENSRNOG00000019711	-0.60	0.0352	-1.52
Srgap3	SLIT-ROBO Rho GTPase activating protein 3	ENSRNOG00000006509	-0.60	0.0271	-1.52
Lyrm2	LYR motif containing 2	ENSRNOG00000043105	-0.60	0.0407	-1.52
Lrig1	leucine-rich repeats and immunoglobulin-like domains 1	ENSRNOG00000012952	-0.60	0.0226	-1.52
Tex2	testis expressed 2	ENSRNOG00000013659	-0.60	0.0275	-1.52
Sema4d	sema domain, immunoglobulin domain (Ig), transmembrane domain (TM) and short cytoplasmic domain	ENSRNOG00000013679	-0.60	0.0212	-1.52
Mknk1	MAP kinase-interacting serine/threonine kinase 1	ENSRNOG00000010381	-0.60	0.0342	-1.52
Ndufa12	NADH dehydrogenase (ubiquinone) 1 alpha subcomplex, 12	ENSRNOG00000007407	-0.60	0.0248	-1.52
Prex1	phosphatidylinositol-3,4,5-trisphosphate-dependent Rac exchange factor 1	ENSRNOG00000006952	-0.60	0.0304	-1.52
Patz1	POZ (BTB) and AT hook containing zinc finger 1	ENSRNOG00000018709	-0.61	0.0459	-1.52
C2cd2	C2 calcium-dependent domain containing 2	ENSRNOG00000001621	-0.61	0.0173	-1.52
Ndrg3	NDRG family member 3	ENSRNOG00000036813	-0.61	0.0293	-1.52
Atp6v0e2	ATPase, H+ transporting V0 subunit e2	ENSRNOG00000008218	-0.61	0.0367	-1.52
Mrpl16	mitochondrial ribosomal protein L16	ENSRNOG00000021005	-0.61	0.0459	-1.52
Fgf7	fibroblast growth factor 7	ENSRNOG00000009425	-0.61	0.0163	-1.52
Colgalt2	collagen beta(1-O)galactosyltransferase 2	ENSRNOG00000028207	-0.61	0.0346	-1.52
Sept8	septin 8	ENSRNOG00000007462	-0.61	0.0269	-1.53
Epb41l3	erythrocyte membrane protein band 4.1-like 3	ENSRNOG00000016724	-0.61	0.0164	-1.53
Mpp5	membrane protein, palmitoylated 5 (MAGUK p55 subfamily member 5)	ENSRNOG00000008788	-0.61	0.0342	-1.53
Rpl3l	ribosomal protein L3-like	ENSRNOG00000014641	-0.61	0.0420	-1.53
Gbp2	guanylate binding protein 2, interferon-inducible	ENSRNOG00000031743	-0.61	0.0212	-1.53
Scarf1	scavenger receptor class F, member 1	ENSRNOG00000037268	-0.61	0.0152	-1.53
Cpeb2	cytoplasmic polyadenylation element binding protein 2	ENSRNOG00000005043	-0.62	0.0277	-1.53

Gene Symbol	Description	Ensembl ID	fold change log2	p value	fold change
Glrx5	glutaredoxin 5	ENSRNOG00000004206	-0.62	0.0254	-1.53
Hs6st2	heparan sulfate 6-O-sulfotransferase 2	ENSRNOG00000030880	-0.62	0.0276	-1.53
LOC691920	similar to kinesin-like motor protein C20orf23	ENSRNOG00000010050	-0.62	0.0356	-1.53
Ak2	adenylate kinase 2	ENSRNOG00000000122	-0.62	0.0363	-1.53
Map3k7cl	MAP3K7 C-terminal like	ENSRNOG00000001584	-0.62	0.0228	-1.53
Xrcc5	X-ray repair complementing defective repair in Chinese hamster cells 5	ENSRNOG00000016105	-0.62	0.0405	-1.53
Tlr3	toll-like receptor 3	ENSRNOG00000021726	-0.62	0.0303	-1.54
Cav3	caveolin 3	ENSRNOG00000005798	-0.62	0.0382	-1.54
Ndufa8	NADH dehydrogenase (ubiquinone) 1 alpha subcomplex, 8	ENSRNOG00000005668	-0.62	0.0371	-1.54
Dars2	aspartyl-tRNA synthetase 2 (mitochondrial)	ENSRNOG00000002813	-0.62	0.0445	-1.54
Mpz	myelin protein zero	ENSRNOG00000003171	-0.62	0.0216	-1.54
Msh3	mutS homolog 3	ENSRNOG00000013673	-0.62	0.0341	-1.54
Naa38	N(alpha)-acetyltransferase 38, NatC auxiliary subunit	ENSRNOG00000009823	-0.62	0.0175	-1.54
Gzf1	GDNF-inducible zinc finger protein 1	ENSRNOG00000004735	-0.62	0.0282	-1.54
Nmrk1	nicotinamide riboside kinase 1	ENSRNOG00000012665	-0.62	0.0236	-1.54
LOC690271	similar to mitochondrial ribosomal protein S11	ENSRNOG00000003080	-0.62	0.0314	-1.54
Sbk1	SH3 domain binding kinase 1	ENSRNOG00000019082	-0.62	0.0360	-1.54
Ecel1	endothelin converting enzyme-like 1	ENSRNOG00000019447	-0.63	0.0184	-1.54
Atp2b3	ATPase, Ca++ transporting, plasma membrane 3	ENSRNOG00000017798	-0.63	0.0445	-1.54
Smap2	small ArfGAP2	ENSRNOG00000011421	-0.63	0.0369	-1.54
Uqcrc1	ubiquinol-cytochrome c reductase core protein I	ENSRNOG00000032134	-0.63	0.0244	-1.54
Dennd4b	DENN/MADD domain containing 4B	ENSRNOG00000022373	-0.63	0.0335	-1.54
Abcb7	ATP-binding cassette, subfamily B (MDR/TAP), member 7	ENSRNOG00000002790	-0.63	0.0479	-1.55
Ltbp3	latent transforming growth factor beta binding protein 3	ENSRNOG00000020813	-0.63	0.0370	-1.55
Jazf1	JAZF zinc finger 1	ENSRNOG00000027026	-0.63	0.0373	-1.55

Gene Symbol	Description	Ensembl ID	fold change log2	p value	fold change
Fyb	FYN binding protein	ENSRNOG00000013886	-0.63	0.0327	-1.55
Pank1	pantothenate kinase 1	ENSRNOG00000018944	-0.63	0.0177	-1.55
Hmbs	hydroxymethylbilane synthase	ENSRNOG00000010390	-0.64	0.0414	-1.55
Ndufs5	NADH dehydrogenase (ubiquinone) Fe-S protein 5	ENSRNOG00000029339	-0.64	0.0184	-1.56
F1LUT6	Uncharacterized protein	ENSRNOG00000000014	-0.64	0.0186	-1.56
Stradb	STE20-related kinase adaptor beta	ENSRNOG00000010728	-0.64	0.0352	-1.56
LOC685888	hypothetical protein LOC685888	ENSRNOG00000028087	-0.64	0.0202	-1.56
Duxbl1	double homeobox B-like 1	ENSRNOG00000025408	-0.64	0.0165	-1.56
Mylk3	myosin light chain kinase 3	ENSRNOG00000017546	-0.64	0.0338	-1.56
Cpne7	copine VII	ENSRNOG00000015397	-0.64	0.0477	-1.56
Mfn2	Mitofusin-2	ENSRNOG00000006904	-0.64	0.0421	-1.56
Smim19	small integral membrane protein 19	ENSRNOG00000024930	-0.64	0.0416	-1.56
Ifi47	interferon gamma inducible protein 47	ENSRNOG00000002470	-0.64	0.0342	-1.56
Lpp	LIM domain containing preferred translocation partner in lipoma	ENSRNOG00000031669	-0.64	0.0303	-1.56
Dusp22	dual specificity phosphatase 22	ENSRNOG00000018218	-0.64	0.0316	-1.56
Mapk10	mitogen activated protein kinase 10	ENSRNOG00000002079	-0.64	0.0473	-1.56
Prkaca	protein kinase, cAMP-dependent, catalytic, alpha	ENSRNOG00000005257	-0.64	0.0319	-1.56
Napepld	N-acyl phosphatidylethanolamine phospholipase D	ENSRNOG00000011363	-0.64	0.0316	-1.56
Hspd1	heat shock protein 1 (chaperonin)	ENSRNOG00000014525	-0.64	0.0360	-1.56
Oxsm	3-oxoacyl-ACP synthase, mitochondrial	ENSRNOG00000005993	-0.65	0.0285	-1.56
Fam20c	family with sequence similarity 20, member C	ENSRNOG00000001314	-0.65	0.0271	-1.57
Wdr37	WD repeat domain 37	ENSRNOG00000016834	-0.65	0.0190	-1.57
Slc22a17	solute carrier family 22, member 17	ENSRNOG00000016414	-0.65	0.0280	-1.57
Tmx2	thioredoxin-related transmembrane protein 2	ENSRNOG00000005308	-0.65	0.0291	-1.57
Ubqln4	ubiquilin 4	ENSRNOG00000019933	-0.65	0.0290	-1.57

Gene Symbol	Description	Ensembl ID	fold change log2	p value	fold change
Ankrd52	ankyrin repeat domain 52	ENSRNOG00000030597	-0.65	0.0323	-1.57
Tle2	transducin-like enhancer of split 2	ENSRNOG00000005874	-0.65	0.0303	-1.57
Ndn	necdin, melanoma antigen (MAGE) family member	ENSRNOG00000010146	-0.65	0.0236	-1.57
RGD1309540	similar to hypothetical protein MGC40841; similar to hypothetical protein MGC4707	ENSRNOG00000014798	-0.65	0.0391	-1.57
Gfra1	GDNF family receptor alpha 1	ENSRNOG00000017438	-0.66	0.0403	-1.57
Leprel2	leprecan-like 2	ENSRNOG00000016071	-0.66	0.0330	-1.58
Kcnb1	Uncharacterized protein	ENSRNOG00000008204	-0.66	0.0324	-1.58
Efhc1	EF-hand domain (C-terminal) containing 1	ENSRNOG00000042729	-0.66	0.0418	-1.58
Ppat	phosphoribosyl pyrophosphate amidotransferase	ENSRNOG00000002128	-0.66	0.0273	-1.58
Nid2	nidogen 2 (osteonidogen)	ENSRNOG00000000341	-0.66	0.0239	-1.58
Sms	spermine synthase	ENSRNOG00000007688	-0.66	0.0283	-1.58
Mfap3l	microfibrillar-associated protein 3-like	ENSRNOG00000011775	-0.66	0.0296	-1.58
Kcnj3	potassium inwardly-rectifying channel, subfamily J, member 3	ENSRNOG00000005369	-0.66	0.0159	-1.58
Pdgfa	platelet-derived growth factor alpha polypeptide	ENSRNOG00000001312	-0.66	0.0315	-1.58
Mtfp1	mitochondrial fission process 1	ENSRNOG00000004640	-0.66	0.0329	-1.58
Fundc1	FUN14 domain containing 1	ENSRNOG00000003470	-0.66	0.0298	-1.58
RGD1561277	RGD1561277	ENSRNOG00000023431	-0.66	0.0233	-1.58
Emc9	ER membrane protein complex subunit 9	ENSRNOG00000019162	-0.66	0.0479	-1.58
Dpep2	dipeptidase 2	ENSRNOG00000023303	-0.66	0.0486	-1.58
Acn9	ACN9 homolog (S. cerevisiae)	ENSRNOG00000011283	-0.66	0.0320	-1.58
Bcl9	B-cell CLL/lymphoma 9	ENSRNOG00000017516	-0.66	0.0275	-1.58
Armc1	armadillo repeat containing 1	ENSRNOG00000013253	-0.66	0.0179	-1.58
Zfp870	zinc finger protein 870	ENSRNOG00000030416	-0.66	0.0185	-1.58
Trps1	trichorhinophalangeal syndrome I	ENSRNOG00000024998	-0.66	0.0463	-1.58
Adam33	ADAM metallopeptidase domain 33	ENSRNOG00000021242	-0.66	0.0209	-1.58

Gene Symbol	Description	Ensembl ID	fold change log2	p value	fold change
Gng7	guanine nucleotide binding protein (G protein), gamma 7	ENSRNOG00000019857	-0.66	0.0154	-1.58
Pla2g4e	phospholipase A2, group IVE	ENSRNOG00000024904	-0.66	0.0374	-1.58
Lrrc75a	leucine rich repeat containing 75A	ENSRNOG00000027286	-0.66	0.0218	-1.58
Agr2	anterior gradient 2	ENSRNOG00000005023	-0.66	0.0275	-1.58
Zfp692	zinc finger protein 692	ENSRNOG00000002682	-0.66	0.0209	-1.58
Guf1	GUF1 GTPase homolog (S. cerevisiae)	ENSRNOG00000002207	-0.66	0.0346	-1.58
Zfhx4	zinc finger homeobox 4	ENSRNOG00000008765	-0.66	0.0230	-1.58
Ptpn14	protein tyrosine phosphatase, non-receptor type 14	ENSRNOG00000003407	-0.66	0.0255	-1.58
Sdpr	serum deprivation response	ENSRNOG00000025895	-0.66	0.0295	-1.59
Unc119	UNC-119 homolog (C. elegans)	ENSRNOG00000011060	-0.67	0.0407	-1.59
Deptor	DEP domain containing MTOR-interacting protein	ENSRNOG00000004328	-0.67	0.0178	-1.59
Fkbp14	FK506 binding protein 14	ENSRNOG00000009886	-0.67	0.0435	-1.59
Trak1	trafficking protein, kinesin binding 1	ENSRNOG00000019262	-0.67	0.0184	-1.59
Rufy2	RUN and FYVE domain-containing protein 2-like	ENSRNOG00000000390	-0.67	0.0179	-1.59
Hibch	3-hydroxyisobutyryl-CoA hydrolase	ENSRNOG00000028557	-0.67	0.0332	-1.59
Ptprq	protein tyrosine phosphatase, receptor type, Q	ENSRNOG00000032479	-0.67	0.0500	-1.59
Nsg1	neuron specific gene family member 1	ENSRNOG00000005700	-0.67	0.0397	-1.59
Fabp3	fatty acid binding protein 3, muscle and heart	ENSRNOG00000012879	-0.67	0.0341	-1.59
Irf9	interferon regulatory factor 9	ENSRNOG00000019478	-0.67	0.0480	-1.60
Trim45	tripartite motif-containing 45	ENSRNOG00000015347	-0.68	0.0209	-1.60
Reps2	RALBP1 associated Eps domain containing protein 2	ENSRNOG00000026846	-0.68	0.0290	-1.60
Cdk5	cyclin-dependent kinase 5	ENSRNOG00000008017	-0.68	0.0323	-1.60
Gga2	golgi associated, gamma adaptin ear containing, ARF binding protein 2	ENSRNOG00000018599	-0.68	0.0363	-1.60
Smarca2	SWI/SNF related, matrix associated, actin dependent regulator of chromatin, subfamily a, member 2	ENSRNOG00000011931	-0.68	0.0142	-1.60
Atf5	activating transcription factor 5	ENSRNOG00000020060	-0.68	0.0280	-1.60

Gene Symbol	Description	Ensembl ID	fold change log2	p value	fold change
Cnnm4	cyclin M4	ENSRNOG00000015886	-0.68	0.0238	-1.60
Stard10	StAR-related lipid transfer (START) domain containing 10	ENSRNOG00000019491	-0.68	0.0486	-1.60
Scrn3	secernin 3	ENSRNOG00000018657	-0.68	0.0275	-1.60
Epn3	epsin 3	ENSRNOG00000003284	-0.68	0.0133	-1.60
Itgav	integrin alpha V	ENSRNOG00000004912	-0.68	0.0278	-1.60
Axin2	axin 2	ENSRNOG00000003612	-0.68	0.0134	-1.60
Akap12	A kinase (PRKA) anchor protein 12	ENSRNOG00000019549	-0.68	0.0420	-1.60
Samhd1	SAM domain and HD domain, 1	ENSRNOG00000006418	-0.68	0.0394	-1.61
Hspa2	heat shock protein 2	ENSRNOG00000006472	-0.68	0.0281	-1.61
LOC100361946	nucleotide-binding oligomerization domain containing 2-like	ENSRNOG00000001872	-0.69	0.0167	-1.61
Lgals3bp	lectin, galactoside-binding, soluble, 3 binding protein	ENSRNOG00000003217	-0.69	0.0178	-1.61
Rimkla	ribosomal modification protein rimK-like family member A	ENSRNOG00000008625	-0.69	0.0177	-1.61
Afap1l1	actin filament associated protein 1-like 1	ENSRNOG00000019403	-0.69	0.0139	-1.61
Flt3lg	fms-related tyrosine kinase 3 ligand-like	ENSRNOG00000042254	-0.69	0.0238	-1.61
Spopl	speckle-type POZ protein-like	ENSRNOG00000005070	-0.69	0.0337	-1.61
RGD1304963	similar to hypothetical protein MGC38716	ENSRNOG00000008942	-0.69	0.0432	-1.61
Ano8	anoctamin 8	ENSRNOG00000017567	-0.69	0.0405	-1.61
RGD1561832	zinc finger protein 658-like	ENSRNOG00000023233	-0.69	0.0416	-1.61
Fgf9	fibroblast growth factor 9	ENSRNOG00000011471	-0.69	0.0155	-1.62
Ric8b	RIC8 guanine nucleotide exchange factor B	ENSRNOG00000007323	-0.69	0.0275	-1.62
Ppp2r5b	protein phosphatase 2, regulatory subunit B', beta	ENSRNOG00000021025	-0.69	0.0333	-1.62
Ankrd9	ankyrin repeat domain 9	ENSRNOG00000008056	-0.69	0.0441	-1.62
Ndufv1	NADH dehydrogenase (ubiquinone) flavoprotein 1	ENSRNOG00000018117	-0.69	0.0218	-1.62
Adamts3	ADAM metallopeptidase with thrombospondin type 1, motif 3	ENSRNOG00000027463	-0.70	0.0498	-1.62
Smc6	structural maintenance of chromosomes 6	ENSRNOG00000004908	-0.70	0.0243	-1.62

Gene Symbol	Description	Ensembl ID	fold change log2	p value	fold change
H2afv	H2A histone family, member V	ENSRNOG00000007026	-0.70	0.0342	-1.62
Hyal3	hyaluronoglucosaminidase 3	ENSRNOG00000016093	-0.70	0.0212	-1.62
Cyc1	cytochrome c-1	ENSRNOG00000012457	-0.70	0.0197	-1.62
Thbs3	thrombospondin 3	ENSRNOG00000020524	-0.70	0.0151	-1.62
Hoxc5	homeo box C5	ENSRNOG00000016598	-0.70	0.0178	-1.62
LOC306766	hypothetical LOC306766	ENSRNOG00000017133	-0.70	0.0186	-1.63
Hadh	hydroxyacyl-CoA dehydrogenase	ENSRNOG00000010697	-0.70	0.0142	-1.63
Trpc1	transient receptor potential cation channel, subfamily C, member 1	ENSRNOG00000009601	-0.70	0.0471	-1.63
Slc25a35	solute carrier family 25, member 35	ENSRNOG00000004668	-0.70	0.0420	-1.63
Socs2	suppressor of cytokine signaling 2	ENSRNOG00000008965	-0.70	0.0433	-1.63
Stard8	StAR-related lipid transfer (START) domain containing 8	ENSRNOG00000033883	-0.70	0.0233	-1.63
Man2b2	mannosidase, alpha, class 2B, member 2	ENSRNOG00000005526	-0.71	0.0383	-1.63
Fgf11	fibroblast growth factor 11	ENSRNOG00000014882	-0.71	0.0441	-1.63
Pyurfl1	PIGY upstream reading frame-like 1	ENSRNOG00000006858	-0.71	0.0475	-1.63
Ptgfrn	prostaglandin F2 receptor inhibitor	ENSRNOG00000015655	-0.71	0.0202	-1.63
LOC100174910	glutaredoxin-like protein	ENSRNOG00000013738	-0.71	0.0147	-1.63
Herc1	HECT and RLD domain containing E3 ubiquitin protein ligase family member 1	ENSRNOG00000043162	-0.71	0.0197	-1.63
Entpd6	ectonucleoside triphosphate diphosphohydrolase 6	ENSRNOG00000007427	-0.71	0.0341	-1.64
Ncald	neurocalcin delta	ENSRNOG00000042978	-0.71	0.0178	-1.64
Akr1b1	aldo-keto reductase family 1, member B1 (aldose reductase)	ENSRNOG00000009513	-0.71	0.0172	-1.64
Sh3bgr	SH3 domain binding glutamate-rich protein	ENSRNOG00000028238	-0.71	0.0200	-1.64
Fam53b	family with sequence similarity 53, member B	ENSRNOG00000017125	-0.71	0.0493	-1.64
Herc1	HECT and RLD domain containing E3 ubiquitin protein ligase family member 1	ENSRNOG00000017592	-0.71	0.0223	-1.64
Atp5g1	ATP synthase, H+ transporting, mitochondrial Fo complex, subunit C1 (subunit 9)	ENSRNOG00000007235	-0.71	0.0341	-1.64

Gene Symbol	Description	Ensembl ID	fold change log2	p value	fold change
Plekha2	pleckstrin homology domain-containing, family A (phosphoinositide binding specific) member 2	ENSRNOG00000038365	-0.72	0.0275	-1.64
Zswim7	zinc finger, SWIM-type containing 7	ENSRNOG00000002970	-0.72	0.0367	-1.64
Zfp827	zinc finger protein 827	ENSRNOG00000011697	-0.72	0.0342	-1.64
Ctnnal1	catenin (cadherin associated protein), alpha-like 1	ENSRNOG00000010593	-0.72	0.0296	-1.64
Ints2	integrator complex subunit 2	ENSRNOG00000003576	-0.72	0.0275	-1.64
Gria4	glutamate receptor, ionotropic, AMPA 4	ENSRNOG00000006957	-0.72	0.0163	-1.64
CAMK2A	Uncharacterized protein	ENSRNOG00000018712	-0.72	0.0160	-1.65
Hddc2	HD domain containing 2	ENSRNOG00000021442	-0.72	0.0323	-1.65
Cenpp	hypothetical protein LOC679342	ENSRNOG00000015545	-0.72	0.0351	-1.65
RGD1302996	hypothetical protein MGC:15854	ENSRNOG00000000812	-0.72	0.0477	-1.65
Gen1	GEN1 Holliday junction 5' flap endonuclease	ENSRNOG00000004667	-0.72	0.0303	-1.65
Map6d1	MAP6 domain containing 1	ENSRNOG00000001911	-0.72	0.0133	-1.65
Bcam	basal cell adhesion molecule (Lutheran blood group)	ENSRNOG00000029399	-0.72	0.0197	-1.65
Abca8a	ATP-binding cassette, subfamily A (ABC1), member 8a	ENSRNOG00000004147	-0.72	0.0441	-1.65
Acsf3	acyl-CoA synthetase family member 3	ENSRNOG00000015077	-0.72	0.0479	-1.65
Apol9a	apolipoprotein L 9a	ENSRNOG00000023410	-0.72	0.0172	-1.65
Col6a1	collagen, type VI, alpha 1	ENSRNOG00000001249	-0.72	0.0220	-1.65
Nkain1	Na+/K+ transporting ATPase interacting 1	ENSRNOG00000011445	-0.72	0.0453	-1.65
Vegfb	vascular endothelial growth factor B	ENSRNOG00000021156	-0.73	0.0275	-1.65
Nudt15	nudix (nucleoside diphosphate linked moiety X)-type motif 15	ENSRNOG00000025239	-0.73	0.0224	-1.66
Hdhd2	haloacid dehalogenase-like hydrolase domain containing 2	ENSRNOG00000043171	-0.73	0.0342	-1.66
Ndufb5	NADH dehydrogenase (ubiquinone) 1 beta subcomplex, 5	ENSRNOG00000011949	-0.73	0.0300	-1.66
Rasl10a	RAS-like, family 10, member A	ENSRNOG00000008951	-0.73	0.0181	-1.66
Pigq	phosphatidylinositol glycan anchor biosynthesis, class Q	ENSRNOG00000020140	-0.73	0.0211	-1.66
Ppil3	peptidylprolyl isomerase (cyclophilin)-like 3	ENSRNOG00000013636	-0.73	0.0435	-1.66

Gene Symbol	Description	Ensembl ID	fold change log2	p value	fold change
Limch1	LIM and calponin homology domains 1	ENSRNOG00000002318	-0.73	0.0318	-1.66
Atp5o	ATP synthase, H+ transporting, mitochondrial F1 complex, O subunit	ENSRNOG00000001991	-0.73	0.0363	-1.66
RGD1564845	similar to Xlr-like	ENSRNOG00000027907	-0.73	0.0187	-1.66
Smo	smoothened, frizzled class receptor	ENSRNOG00000008332	-0.73	0.0162	-1.66
Lsm14b	LSM14B, SCD6 homolog B (S. cerevisiae)	ENSRNOG00000008877	-0.73	0.0310	-1.66
Atf7ip	activating transcription factor 7 interacting protein	ENSRNOG00000008870	-0.73	0.0409	-1.66
Nmnat1	nicotinamide nucleotide adenylyltransferase 1	ENSRNOG00000015962	-0.73	0.0142	-1.66
Hopx	HOP homeobox	ENSRNOG00000024689	-0.74	0.0225	-1.66
Pip4k2b	phosphatidylinositol-5-phosphate 4-kinase, type II, beta	ENSRNOG00000013030	-0.74	0.0341	-1.66
Prkra	protein kinase, interferon inducible double stranded RNA dependent activator	ENSRNOG00000011195	-0.74	0.0323	-1.67
Ndufb3	NADH dehydrogenase (ubiquinone) 1 beta subcomplex 3-like	ENSRNOG00000011825	-0.74	0.0297	-1.67
Desi2	desumoylating isopeptidase 2	ENSRNOG00000004524	-0.74	0.0323	-1.67
Hif1an	hypoxia-inducible factor 1, alpha subunit inhibitor	ENSRNOG00000014234	-0.74	0.0356	-1.67
Acadm	acyl-CoA dehydrogenase, C-4 to C-12 straight chain	ENSRNOG00000009845	-0.74	0.0303	-1.67
Sdk2	sidekick cell adhesion molecule 2	ENSRNOG00000024711	-0.74	0.0155	-1.67
Mrps11	mitochondrial ribosomal protein S11	ENSRNOG00000018531	-0.74	0.0181	-1.67
Invs	inversin	ENSRNOG00000008632	-0.74	0.0374	-1.67
Ndufa2	NADH dehydrogenase (ubiquinone) 1 alpha subcomplex, 2	ENSRNOG00000017571	-0.74	0.0309	-1.67
Stim1	stromal interaction molecule 1	ENSRNOG00000020425	-0.74	0.0216	-1.67
Phkg1	phosphorylase kinase, gamma 1	ENSRNOG00000000920	-0.75	0.0417	-1.68
Pomk	protein-O-mannose kinase	ENSRNOG00000014628	-0.75	0.0494	-1.68
Hlf	hepatic leukemia factor	ENSRNOG00000002456	-0.75	0.0159	-1.68
LOC500350	LRRGT00139	ENSRNOG00000030158	-0.75	0.0235	-1.68
Irs1	insulin receptor substrate 1	ENSRNOG00000014597	-0.75	0.0133	-1.68
Hccs	holocytochrome c synthase	ENSRNOG00000025910	-0.75	0.0184	-1.68

Gene Symbol	Description	Ensembl ID	fold change log2	p value	fold change
Cacybp	calcyclin binding protein	ENSRNOG00000002572	-0.75	0.0258	-1.68
Slc9a2	solute carrier family 9, subfamily A (NHE2, cation proton antiporter 2), member 2	ENSRNOG00000015567	-0.75	0.0350	-1.69
Vamp1	vesicle-associated membrane protein 1	ENSRNOG00000019219	-0.75	0.0345	-1.69
RT1-T24-4	RT1 class I, locus T24, gene 4	ENSRNOG00000042905	-0.76	0.0224	-1.69
RGD1561206	similar to solute carrier family 25 (mitochondrial deoxynucleotide carrier), member 19	ENSRNOG00000016331	-0.76	0.0197	-1.69
Tstd3	thiosulfate sulfurtransferase (rhodanese)-like domain containing 3	ENSRNOG00000028185	-0.76	0.0212	-1.69
Abcd1	ATP-binding cassette, subfamily D (ALD), member 1	ENSRNOG00000018857	-0.76	0.0307	-1.69
Fmnl1	formin-like 1	ENSRNOG00000003207	-0.76	0.0166	-1.69
Klf12	Kruppel-like factor 12	ENSRNOG00000009145	-0.76	0.0249	-1.69
Atp5i	ATP synthase, H+ transporting, mitochondrial Fo complex, subunit E	ENSRNOG00000000064	-0.76	0.0163	-1.69
Psme2	proteasome (prosome, macropain) activator subunit 2	ENSRNOG00000019246	-0.76	0.0301	-1.69
Adtrp	androgen-dependent TFPI-regulating protein	ENSRNOG00000014481	-0.76	0.0342	-1.69
Lrsam1	leucine rich repeat and sterile alpha motif containing 1	ENSRNOG00000022312	-0.76	0.0223	-1.70
Cacna2d1	calcium channel, voltage-dependent, alpha2/delta subunit 1	ENSRNOG00000033531	-0.76	0.0307	-1.70
Slc37a4	solute carrier family 37 (glucose-6-phosphate transporter), member 4	ENSRNOG00000011361	-0.76	0.0327	-1.70
Pcyox1	prenylcysteine oxidase 1	ENSRNOG00000016704	-0.76	0.0244	-1.70
Parp14	poly (ADP-ribose) polymerase family, member 14	ENSRNOG00000023334	-0.77	0.0290	-1.70
Cacna2d3	calcium channel, voltage-dependent, alpha2/delta subunit 3	ENSRNOG00000031287	-0.77	0.0133	-1.70
Rassf3	Ras association (RalGDS/AF-6) domain family member 3	ENSRNOG00000005388	-0.77	0.0360	-1.70
Cars2	cysteinyl-tRNA synthetase 2, mitochondrial	ENSRNOG00000014526	-0.77	0.0230	-1.70
Fastk	Fas-activated serine/threonine kinase	ENSRNOG00000011667	-0.77	0.0261	-1.70
Copg1	coatomer protein complex, subunit gamma 1	ENSRNOG00000010474	-0.77	0.0170	-1.70
Entpd2	ectonucleoside triphosphate diphosphohydrolase 2	ENSRNOG00000013102	-0.77	0.0354	-1.70
Lrrc55	leucine rich repeat containing 55	ENSRNOG00000028266	-0.77	0.0269	-1.70
Ndufs8	NADH dehydrogenase (ubiquinone) Fe-S protein 8	ENSRNOG00000017446	-0.77	0.0300	-1.70

Gene Symbol	Description	Ensembl ID	fold change log2	p value	fold change
Mlkl	mixed lineage kinase domain-like	ENSRNOG00000042353	-0.77	0.0199	-1.71
Mlec	malectin	ENSRNOG00000021725	-0.77	0.0253	-1.71
Coa5	cytochrome C oxidase assembly factor 5	ENSRNOG00000018102	-0.77	0.0449	-1.71
Maged2	melanoma antigen, family D, 2	ENSRNOG00000002449	-0.77	0.0409	-1.71
Rnf128	ring finger protein 128, E3 ubiquitin protein ligase	ENSRNOG00000043123	-0.77	0.0147	-1.71
Inppl1	inositol polyphosphate phosphatase-like 1	ENSRNOG00000019730	-0.77	0.0113	-1.71
Cdk6	cyclin-dependent kinase 6	ENSRNOG00000009258	-0.78	0.0142	-1.71
Igsf10	immunoglobulin superfamily, member 10	ENSRNOG00000013917	-0.78	0.0246	-1.71
Mrpl48	mitochondrial ribosomal protein L48	ENSRNOG00000018042	-0.78	0.0414	-1.71
Siae	sialic acid acetylesterase	ENSRNOG00000031266	-0.78	0.0307	-1.72
Endod1	endonuclease domain containing 1	ENSRNOG00000024757	-0.78	0.0236	-1.72
Isoc2b	isochorismatase domain containing 2b	ENSRNOG00000016829	-0.78	0.0142	-1.72
Tnfaip2	tumor necrosis factor, alpha-induced protein 2	ENSRNOG00000010165	-0.78	0.0136	-1.72
Adck1	aarF domain containing kinase 1	ENSRNOG00000012685	-0.78	0.0342	-1.72
Nudt7	nudix (nucleoside diphosphate linked moiety X)-type motif 7	ENSRNOG00000011976	-0.78	0.0229	-1.72
Bola3	bolA family member 3	ENSRNOG00000021866	-0.78	0.0212	-1.72
Inpp5f	inositol polyphosphate-5-phosphatase F	ENSRNOG00000020388	-0.79	0.0223	-1.72
Mki67	marker of proliferation Ki-67	ENSRNOG00000028137	-0.79	0.0370	-1.72
Phlda3	pleckstrin homology-like domain, family A, member 3	ENSRNOG00000009068	-0.79	0.0374	-1.72
Sufu	suppressor of fused homolog (Drosophila)	ENSRNOG00000019807	-0.79	0.0202	-1.73
Timm21	translocase of inner mitochondrial membrane 21 homolog (yeast)	ENSRNOG00000015142	-0.79	0.0341	-1.73
Sema5a	Sema domain, seven thrombospondin repeats (type 1 and type 1-like), transmembrane domain (TM) and short cytoplasmic domain, (semaphorin) 5A	ENSRNOG00000011977	-0.79	0.0385	-1.73
Hspg2	perlecan (heparan sulfate proteoglycan 2)	ENSRNOG00000021437	-0.79	0.0101	-1.73
Aadacl3	arylacetamide deacetylase-like 3	ENSRNOG00000026613	-0.79	0.0238	-1.73

Gene Symbol	Description	Ensembl ID	fold change log2	p value	fold change
Pkhd1l1	polycystic kidney and hepatic disease 1-like 1	ENSRNOG00000004398	-0.79	0.0437	-1.73
Atp5g3	ATP synthase, H+ transporting, mitochondrial Fo complex, subunit C3 (subunit 9)	ENSRNOG00000001596	-0.79	0.0216	-1.73
Rab11fip3	RAB11 family interacting protein 3 (class II)	ENSRNOG00000032152	-0.79	0.0420	-1.73
Zfp949	zinc finger protein 949	ENSRNOG00000043223	-0.79	0.0473	-1.73
Flad1	flavin adenine dinucleotide synthetase 1	ENSRNOG00000020642	-0.79	0.0283	-1.73
Mdp1	magnesium-dependent phosphatase 1	ENSRNOG00000019840	-0.79	0.0075	-1.73
Ngfrap1	nerve growth factor receptor (TNFRSF16) associated protein 1	ENSRNOG00000028822	-0.80	0.0477	-1.74
Extl1	exostosin-like glycosyltransferase 1	ENSRNOG00000016776	-0.80	0.0460	-1.74
Mbd4	methyl-CpG binding domain protein 4	ENSRNOG00000010919	-0.80	0.0421	-1.74
Eci1	enoyl-CoA delta isomerase 1	ENSRNOG00000008843	-0.80	0.0374	-1.74
Sema6a	sema domain, transmembrane domain (TM), and cytoplasmic domain, (semaphorin) 6A	ENSRNOG00000004033	-0.80	0.0304	-1.74
Fam212b	family with sequence similarity 212, member B	ENSRNOG00000015691	-0.80	0.0129	-1.74
Slc35b4	solute carrier family 35 (UDP-xylose/UDP-N-acetylglucosamine transporter), member B4	ENSRNOG00000008851	-0.80	0.0279	-1.74
Dgkb	diacylglycerol kinase, beta	ENSRNOG00000030771	-0.80	0.0223	-1.74
RGD1309104	similar to RIKEN cDNA 1700025G04 gene	ENSRNOG00000028236	-0.80	0.0375	-1.74
Dnajc27	DnaJ (Hsp40) homolog, subfamily C, member 27	ENSRNOG00000003988	-0.80	0.0441	-1.74
Zfp879	zinc finger protein 879	ENSRNOG00000030517	-0.80	0.0136	-1.75
Rtn4ip1	reticulon 4 interacting protein 1	ENSRNOG00000000279	-0.80	0.0356	-1.75
Raph1	Ras association (RalGDS/AF-6) and pleckstrin homology domains 1	ENSRNOG00000014722	-0.81	0.0165	-1.75
Grm8	glutamate receptor, metabotropic 8	ENSRNOG00000021468	-0.81	0.0389	-1.75
Wrn	Werner syndrome, RecQ helicase-like	ENSRNOG00000015440	-0.81	0.0318	-1.75
RGD1309362	similar to interferon-inducible GTPase	ENSRNOG00000038960	-0.81	0.0212	-1.75
Ccdc141	coiled-coil domain containing 141	ENSRNOG00000012580	-0.81	0.0068	-1.75
RGD1304978	similar to RIKEN cDNA 0610027B03	ENSRNOG00000004926	-0.81	0.0187	-1.76

Gene Symbol	Description	Ensembl ID	fold change log2	p value	fold change
RT1-M6-1	RT1 class Ib, locus M4	ENSRNOG00000022107	-0.81	0.0187	-1.76
Wee1	WEE1 G2 checkpoint kinase	ENSRNOG00000010017	-0.81	0.0118	-1.76
B3galnt2	beta-1,3-N-acetylgalactosaminyltransferase 2	ENSRNOG00000016855	-0.81	0.0476	-1.76
LOC297568	alpha-1-inhibitor III	ENSRNOG00000042228	-0.82	0.0476	-1.76
Oasl2	2'-5' oligoadenylate synthetase-like 2	ENSRNOG00000028814	-0.82	0.0213	-1.76
Zfand1	zinc finger, AN1-type domain 1	ENSRNOG00000010342	-0.82	0.0177	-1.76
Dpf3	D4, zinc and double PHD fingers, family 3	ENSRNOG00000008086	-0.82	0.0177	-1.76
Mrm1	mitochondrial rRNA methyltransferase 1 homolog (S. cerevisiae)	ENSRNOG00000027901	-0.82	0.0179	-1.76
Atpaf1	ATP synthase mitochondrial F1 complex assembly factor 1	ENSRNOG00000010169	-0.82	0.0498	-1.76
Mmp16	matrix metallopeptidase 16	ENSRNOG00000005708	-0.82	0.0323	-1.77
Lpar6	lysophosphatidic acid receptor 6	ENSRNOG00000015577	-0.82	0.0102	-1.77
Tln2	talin 2	ENSRNOG00000018373	-0.82	0.0149	-1.77
Tmem50b	transmembrane protein 50B	ENSRNOG00000002028	-0.82	0.0267	-1.77
Myocd	myocardin	ENSRNOG00000003669	-0.82	0.0484	-1.77
Crem	cAMP responsive element modulator	ENSRNOG00000014900	-0.83	0.0107	-1.77
Cda	cytidine deaminase-like	ENSRNOG00000015677	-0.83	0.0398	-1.77
Lurap1l	leucine rich adaptor protein 1-like	ENSRNOG00000033740	-0.83	0.0101	-1.77
Cmpk2	cytidine monophosphate (UMP-CMP) kinase 2, mitochondrial	ENSRNOG00000007690	-0.83	0.0142	-1.78
Fam189b	family with sequence similarity 189, member B	ENSRNOG00000020518	-0.83	0.0327	-1.78
Cyp2t1	cytochrome P450, family 2, subfamily t, polypeptide 1	ENSRNOG00000028891	-0.83	0.0187	-1.78
Gria3	glutamate receptor, ionotropic, AMPA 3	ENSRNOG00000007682	-0.83	0.0298	-1.78
Elk1	ELK1, member of ETS oncogene family	ENSRNOG00000010171	-0.83	0.0142	-1.78
Slc46a3	solute carrier family 46, member 3	ENSRNOG00000000937	-0.83	0.0195	-1.78
Nr3c2	nuclear receptor subfamily 3, group C, member 2	ENSRNOG00000034007	-0.84	0.0181	-1.78
LOC100912012	tetranectin-like	ENSRNOG00000004540	-0.84	0.0159	-1.79

Gene Symbol	Description	Ensembl ID	fold change log2	p value	fold change
Itga8	integrin, alpha 8	ENSRNOG00000016538	-0.84	0.0206	-1.79
Gdf11	growth differentiation factor 11	ENSRNOG00000007610	-0.84	0.0238	-1.79
Tenm4	teneurin transmembrane protein 4	ENSRNOG00000011151	-0.84	0.0148	-1.79
Acad10	acyl-CoA dehydrogenase family, member 10	ENSRNOG00000037815	-0.84	0.0436	-1.79
Cdc26	cell division cycle 26	ENSRNOG00000029785	-0.84	0.0186	-1.79
Rasgrp3	RAS guanyl releasing protein 3 (calcium and DAG-regulated)	ENSRNOG00000032703	-0.84	0.0441	-1.79
Emx2	empty spiracles homeobox 2	ENSRNOG00000009482	-0.84	0.0186	-1.79
Casq2	calsequestrin 2 (cardiac muscle)	ENSRNOG00000016243	-0.84	0.0345	-1.79
Trim5	tripartite motif-containing 5	ENSRNOG00000017191	-0.84	0.0314	-1.79
Slc39a13	solute carrier family 39 (zinc transporter), member 13	ENSRNOG00000011981	-0.84	0.0142	-1.79
St8sia4	ST8 alpha-N-acetyl-neuraminide alpha-2,8-sialyltransferase 4	ENSRNOG00000019128	-0.84	0.0177	-1.80
Pcsk6	proprotein convertase subtilisin/kexin type 6	ENSRNOG00000011526	-0.84	0.0189	-1.80
Casp12	caspase 12	ENSRNOG00000033434	-0.85	0.0374	-1.80
Shc3	SHC (Src homology 2 domain containing) transforming protein 3	ENSRNOG00000014366	-0.85	0.0320	-1.80
Sim1	single-minded family bHLH transcription factor 1	ENSRNOG00000037600	-0.85	0.0246	-1.80
Col6a2	collagen, type VI, alpha 2	ENSRNOG00000001254	-0.85	0.0108	-1.80
Ccbl1	cysteine conjugate-beta lyase, cytoplasmic	ENSRNOG00000016097	-0.85	0.0466	-1.80
Mstn	myostatin	ENSRNOG00000021294	-0.86	0.0201	-1.81
Slc36a2	solute carrier family 36 (proton/amino acid symporter), member 2	ENSRNOG00000011892	-0.86	0.0219	-1.81
Alox5	arachidonate 5-lipoxygenase	ENSRNOG00000012972	-0.86	0.0253	-1.81
C1qtnf6	C1q and tumor necrosis factor related protein 6	ENSRNOG00000007300	-0.86	0.0306	-1.81
Tmem143	transmembrane protein 143	ENSRNOG00000021096	-0.86	0.0155	-1.81
Atxn1	ataxin 1	ENSRNOG00000016998	-0.86	0.0129	-1.81
Filip1l	filamin A interacting protein 1-like	ENSRNOG00000001645	-0.86	0.0343	-1.81
Ccdc146	coiled-coil domain containing 146	ENSRNOG00000012932	-0.86	0.0191	-1.81

Gene Symbol	Description	Ensembl ID	fold change log2	p value	fold change
Ubac1	UBA domain containing 1	ENSRNOG00000017983	-0.86	0.0329	-1.82
Ltbp4	latent transforming growth factor beta binding protein 4	ENSRNOG00000020871	-0.86	0.0182	-1.82
Ppip5k2	diphosphoinositol pentakisphosphate kinase 2	ENSRNOG00000011613	-0.86	0.0437	-1.82
Tefm	transcription elongation factor, mitochondrial	ENSRNOG00000004000	-0.86	0.0498	-1.82
Slc2a12	solute carrier family 2 (facilitated glucose transporter), member 12	ENSRNOG00000011161	-0.86	0.0178	-1.82
Leprel1	leprecan-like 1	ENSRNOG00000001925	-0.86	0.0230	-1.82
Nudt5	nudix (nucleoside diphosphate linked moiety X)-type motif 5	ENSRNOG00000017741	-0.87	0.0405	-1.82
Stxbp6	syntaxin binding protein 6 (amisyn)	ENSRNOG00000004198	-0.87	0.0125	-1.82
Casq1	calsequestrin 1 (fast-twitch, skeletal muscle)	ENSRNOG00000006930	-0.87	0.0369	-1.82
Smyd1	SET and MYND domain containing 1	ENSRNOG00000006776	-0.87	0.0310	-1.82
Kdelc2	KDEL (Lys-Asp-Glu-Leu) containing 2	ENSRNOG00000007177	-0.87	0.0322	-1.83
Dnm1l	dynamin 1-like	ENSRNOG00000001813	-0.87	0.0307	-1.83
Dkk2	dickkopf WNT signaling pathway inhibitor 2	ENSRNOG00000011360	-0.87	0.0360	-1.83
Wdr92	WD repeat domain 92	ENSRNOG00000022857	-0.87	0.0305	-1.83
LOC100365881	glutathione S-transferase alpha-4-like	ENSRNOG00000000217	-0.87	0.0244	-1.83
Slc25a40	solute carrier family 25, member 40	ENSRNOG00000022837	-0.87	0.0323	-1.83
Mpdz	multiple PDZ domain protein	ENSRNOG00000007894	-0.87	0.0125	-1.83
Perm1	PPARGC1 and ESRR induced regulator, muscle 1	ENSRNOG00000020244	-0.87	0.0494	-1.83
Phf11	PHD finger protein 11	ENSRNOG00000011580	-0.88	0.0178	-1.84
Dock10	dedicator of cytokinesis 10	ENSRNOG00000016165	-0.88	0.0238	-1.84
Kcnt2	potassium channel, subfamily T, member 2	ENSRNOG00000013312	-0.88	0.0374	-1.84
Trim35	tripartite motif-containing 35	ENSRNOG00000009449	-0.88	0.0218	-1.84
Ndufs3	NADH dehydrogenase (ubiquinone) Fe-S protein 3	ENSRNOG00000009155	-0.88	0.0261	-1.85
Cd28	Cd28 molecule	ENSRNOG00000010283	-0.89	0.0453	-1.85
Mpc2	mitochondrial pyruvate carrier 2	ENSRNOG00000003150	-0.89	0.0147	-1.85

Gene Symbol	Description	Ensembl ID	fold change log2	p value	fold change
Gcgr	glucagon receptor	ENSRNOG00000036692	-0.89	0.0281	-1.85
Leprel4	leprecan-like 4	ENSRNOG00000015787	-0.89	0.0184	-1.85
Dach1	dachshund family transcription factor 1	ENSRNOG00000008834	-0.89	0.0113	-1.85
RGD1559731	similar to RIKEN cDNA 4930578C19	ENSRNOG00000004265	-0.89	0.0068	-1.85
Tex9	testis expressed 9	ENSRNOG00000024291	-0.89	0.0417	-1.85
Col6a6	collagen, type VI, alpha 6	ENSRNOG00000023007	-0.89	0.0123	-1.85
Cradd	CASP2 and RIPK1 domain containing adaptor with death domain	ENSRNOG00000008507	-0.89	0.0131	-1.86
Aspa	aspartoacylase	ENSRNOG00000019659	-0.89	0.0180	-1.86
Ccdc92	coiled-coil domain containing 92	ENSRNOG00000021691	-0.89	0.0101	-1.86
Lbx1	ladybird homeobox 1	ENSRNOG00000025520	-0.89	0.0094	-1.86
Frk	fyn-related Src family tyrosine kinase	ENSRNOG00000000543	-0.89	0.0212	-1.86
Zfp763	zinc finger protein 763	ENSRNOG00000004557	-0.90	0.0209	-1.86
Rnaseh2a	ribonuclease H2, subunit A	ENSRNOG00000003504	-0.90	0.0202	-1.86
Mppe1	metallophosphoesterase 1	ENSRNOG00000018648	-0.90	0.0156	-1.87
Kcnma1	potassium large conductance calcium-activated channel, subfamily M, alpha member 1	ENSRNOG00000005985	-0.90	0.0315	-1.87
Slc38a3	solute carrier family 38, member 3	ENSRNOG00000016827	-0.90	0.0360	-1.87
Vldlr	very low density lipoprotein receptor	ENSRNOG00000027491	-0.90	0.0453	-1.87
Ints3	integrator complex subunit 3	ENSRNOG00000015153	-0.90	0.0180	-1.87
Cxadr	coxsackie virus and adenovirus receptor	ENSRNOG00000001557	-0.90	0.0218	-1.87
Zfp606	zinc finger protein 606	ENSRNOG00000019127	-0.90	0.0177	-1.87
Trmt1l	tRNA methyltransferase 1-like	ENSRNOG00000002580	-0.91	0.0343	-1.87
Calcr	calcitonin receptor	ENSRNOG00000010053	-0.91	0.0133	-1.87
Mdh1	malate dehydrogenase 1, NAD (soluble)	ENSRNOG00000008103	-0.91	0.0307	-1.87
Ndufb7	NADH dehydrogenase (ubiquinone) 1 beta subcomplex, 7	ENSRNOG00000028717	-0.91	0.0131	-1.88
Slco3a1	solute carrier organic anion transporter family, member 3a1	ENSRNOG00000032798	-0.91	0.0177	-1.88

Gene Symbol	Description	Ensembl ID	fold change log2	p value	fold change
Mrpl12	mitochondrial ribosomal protein L12	ENSRNOG00000036695	-0.91	0.0212	-1.88
Rfesd	Rieske (Fe-S) domain containing	ENSRNOG00000012810	-0.92	0.0348	-1.89
Clasp1	cytoplasmic linker associated protein 1	ENSRNOG00000002376	-0.92	0.0177	-1.89
Fam118b	family with sequence similarity 118, member B	ENSRNOG00000011543	-0.92	0.0258	-1.89
Ppapdc3	phosphatidic acid phosphatase type 2 domain containing 3	ENSRNOG00000010068	-0.92	0.0100	-1.89
Chordc1	cysteine and histidine-rich domain (CHORD)-containing 1	ENSRNOG00000026643	-0.92	0.0197	-1.89
Vom1r93	vomeronasal 1 receptor 93	ENSRNOG00000017735	-0.92	0.0202	-1.89
Elovl7	ELOVL fatty acid elongase 7	ENSRNOG00000010450	-0.92	0.0136	-1.90
Kcnh2	potassium voltage-gated channel, subfamily H (eag-related), member 2	ENSRNOG00000009872	-0.92	0.0090	-1.90
Aknad1	AKNA domain containing 1	ENSRNOG00000028025	-0.92	0.0073	-1.90
Acyp1	acylphosphatase 1, erythrocyte (common) type	ENSRNOG00000006744	-0.92	0.0163	-1.90
Lmod3	leiomodin 3 (fetal)	ENSRNOG00000032443	-0.92	0.0238	-1.90
C1qtnf2	C1q and tumor necrosis factor related protein 2	ENSRNOG00000003870	-0.92	0.0102	-1.90
Sirt3	sirtuin 3	ENSRNOG00000013828	-0.93	0.0066	-1.90
Gcnt4	glucosaminyl (N-acetyl) transferase 4, core 2	ENSRNOG00000016500	-0.93	0.0067	-1.90
Doc2g	double C2-like domains, gamma	ENSRNOG00000018029	-0.93	0.0342	-1.90
Ramp1	receptor (G protein-coupled) activity modifying protein 1	ENSRNOG00000019926	-0.93	0.0134	-1.90
Ddx60	DEAD (Asp-Glu-Ala-Asp) box polypeptide 60	ENSRNOG00000014541	-0.93	0.0318	-1.90
Pfn2	profilin 2	ENSRNOG00000017427	-0.93	0.0445	-1.91
Fam149a	family with sequence similarity 149, member A	ENSRNOG00000021693	-0.93	0.0275	-1.91
Cntn2	contactin 2 (axonal)	ENSRNOG00000009033	-0.93	0.0133	-1.91
Asah2	N-acylsphingosine amidohydrolase (non-lysosomal ceramidase) 2	ENSRNOG00000012196	-0.93	0.0150	-1.91
Dyrk1b	dual-specificity tyrosine-(Y)-phosphorylation regulated kinase 1b	ENSRNOG00000019254	-0.93	0.0396	-1.91
Ptpn3	protein tyrosine phosphatase, non-receptor type 3	ENSRNOG00000011425	-0.93	0.0397	-1.91
Pde7b	phosphodiesterase 7B	ENSRNOG00000013436	-0.93	0.0276	-1.91

Gene Symbol	Description	Ensembl ID	fold change log2	p value	fold change
Arhgef6	Rac/Cdc42 guanine nucleotide exchange factor (GEF) 6	ENSRNOG00000000869	-0.94	0.0346	-1.91
Pgm5	phosphoglucomutase 5	ENSRNOG00000015406	-0.94	0.0101	-1.91
Adamts19	ADAM metallopeptidase with thrombospondin type 1 motif, 19	ENSRNOG00000019577	-0.94	0.0159	-1.92
Gbp5	guanylate binding protein 5	ENSRNOG00000032240	-0.95	0.0223	-1.93
Mx2	myxovirus (influenza virus) resistance 2	ENSRNOG00000001963	-0.95	0.0398	-1.93
Amy2a3	amylase 2a3	ENSRNOG00000016168	-0.95	0.0479	-1.93
C2cd5	C2 calcium-dependent domain containing 5	ENSRNOG00000014382	-0.95	0.0125	-1.93
Cacng6	calcium channel, voltage-dependent, gamma subunit 6	ENSRNOG00000014326	-0.95	0.0365	-1.93
Zkscan4	zinc finger with KRAB and SCAN domains 4	ENSRNOG00000038818	-0.95	0.0476	-1.93
Dnmt3a	DNA (cytosine-5-)-methyltransferase 3 alpha	ENSRNOG00000026649	-0.95	0.0176	-1.93
Tm6sf1	transmembrane 6 superfamily member 1	ENSRNOG00000019662	-0.95	0.0142	-1.93
Sugct	succinylCoA:glutarate-CoA transferase	ENSRNOG00000014028	-0.95	0.0139	-1.93
Cacnb4	calcium channel, voltage-dependent, beta 4 subunit	ENSRNOG00000007666	-0.95	0.0257	-1.94
Lyrm5	LYR motif containing 5	ENSRNOG00000015848	-0.96	0.0317	-1.94
Sh3kbp1	SH3-domain kinase binding protein 1	ENSRNOG00000004322	-0.96	0.0246	-1.94
Ablim2	actin binding LIM protein family, member 2	ENSRNOG00000007882	-0.96	0.0370	-1.94
Abcd3	ATP-binding cassette, subfamily D (ALD), member 3	ENSRNOG00000011929	-0.96	0.0072	-1.94
Slc25a20	solute carrier family 25 (carnitine/acylcarnitine translocase), member 20	ENSRNOG00000020288	-0.96	0.0179	-1.94
Popdc2	popeye domain containing 2	ENSRNOG00000002994	-0.96	0.0264	-1.95
mrpl24	mitochondrial ribosomal protein L24	ENSRNOG00000022234	-0.96	0.0136	-1.95
Atp1b1	ATPase, Na+/K+ transporting, beta 1 polypeptide	ENSRNOG00000002934	-0.96	0.0227	-1.95
Nudt14	nudix (nucleoside diphosphate linked moiety X)-type motif 14	ENSRNOG00000014362	-0.96	0.0301	-1.95
Ank2	ankyrin 2, neuronal	ENSRNOG00000011076	-0.96	0.0164	-1.95
Ccnd2	cyclin D2	ENSRNOG00000019939	-0.96	0.0177	-1.95
Fam126b	family with sequence similarity 126, member B	ENSRNOG00000025079	-0.97	0.0055	-1.95

Gene Symbol	Description	Ensembl ID	fold change log2	p value	fold change
Coq7	coenzyme Q7 homolog, ubiquinone (yeast)	ENSRNOG00000017012	-0.97	0.0298	-1.95
Chdh	choline dehydrogenase	ENSRNOG00000015859	-0.97	0.0476	-1.95
Ano5	anoctamin 5	ENSRNOG00000015972	-0.97	0.0457	-1.96
Bmp1	bone morphogenetic protein 1	ENSRNOG00000010890	-0.97	0.0051	-1.96
Uba7	ubiquitin-like modifier activating enzyme 7	ENSRNOG00000029195	-0.97	0.0159	-1.96
Angptl1	angiopoietin-like 1	ENSRNOG00000004712	-0.97	0.0277	-1.96
Map7d3	MAP7 domain containing 3	ENSRNOG00000027831	-0.97	0.0391	-1.96
Sec14l5	SEC14-like 5 (S. cerevisiae)	ENSRNOG00000002917	-0.97	0.0133	-1.96
Fuom	fucose mutarotase	ENSRNOG00000018476	-0.97	0.0205	-1.97
RGD1307461	similar to RIKEN cDNA 6430571L13 gene; similar to g20 protein	ENSRNOG00000015718	-0.98	0.0209	-1.97
Car4	carbonic anhydrase 4	ENSRNOG00000002916	-0.98	0.0176	-1.97
Sec16b	SEC16 homolog B (S. cerevisiae)	ENSRNOG00000005229	-0.98	0.0123	-1.97
Tle1	transducin-like enhancer of split 1 (E(sp1) homolog, Drosophila)	ENSRNOG00000005882	-0.98	0.0201	-1.98
Sorl1	sortilin-related receptor, LDLR class A repeats-containing	ENSRNOG00000024374	-0.98	0.0113	-1.98
Ppm1j	protein phosphatase, Mg2+/Mn2+ dependent, 1J	ENSRNOG00000012481	-0.98	0.0061	-1.98
Ankh	ANKH inorganic pyrophosphate transport regulator	ENSRNOG00000010960	-0.98	0.0209	-1.98
Paqr7	progestin and adipoQ receptor family member VII	ENSRNOG00000022054	-0.98	0.0066	-1.98
Gpsm2	G-protein signaling modulator 2	ENSRNOG00000012149	-0.99	0.0090	-1.98
Aldh1a2	aldehyde dehydrogenase 1 family, member A2	ENSRNOG00000016042	-0.99	0.0155	-1.98
Dusp10	dual specificity phosphatase 10	ENSRNOG00000004003	-0.99	0.0257	-1.98
Itgb6	integrin, beta 6	ENSRNOG00000008346	-0.99	0.0104	-1.98
Fkbp4	FK506 binding protein 4	ENSRNOG00000006444	-0.99	0.0316	-1.99
Ptgr2	prostaglandin reductase 2	ENSRNOG00000038166	-0.99	0.0198	-1.99
Cep70	centrosomal protein 70	ENSRNOG00000022845	-0.99	0.0222	-1.99
RGD1561161	similar to BC067074 protein	ENSRNOG00000039582	-0.99	0.0073	-1.99

Gene Symbol	Description	Ensembl ID	fold change log2	p value	fold change
Tmem132a	transmembrane protein 132A	ENSRNOG00000021338	-1.00	0.0178	-1.99
Per3	period circadian clock 3	ENSRNOG00000018413	-1.00	0.0107	-1.99
Prepl	prolyl endopeptidase-like	ENSRNOG00000007326	-1.00	0.0136	-2.00
Fxyd6	FXYD domain-containing ion transport regulator 6	ENSRNOG00000016412	-1.00	0.0043	-2.00
Lrrc17	leucine rich repeat containing 17	ENSRNOG00000012817	-1.00	0.0142	-2.00
Frem1	Fras1 related extracellular matrix 1	ENSRNOG00000022309	-1.01	0.0155	-2.01
Bpnt1	3'(2'), 5'-bisphosphate nucleotidase 1	ENSRNOG00000002378	-1.01	0.0322	-2.01
Cdon	cell adhesion associated, oncogene regulated	ENSRNOG00000011789	-1.01	0.0092	-2.01
Stac3	cell adhesion associated, oncogene regulated	ENSRNOG00000008050	-1.01	0.0096	-2.01
Fndc3c1	fibronectin type III domain containing 3C1	ENSRNOG00000002451	-1.01	0.0187	-2.01
MAMDC2	Uncharacterized protein	ENSRNOG00000024620	-1.01	0.0094	-2.02
Rab15	RAB15, member RAS oncogene family	ENSRNOG00000007364	-1.01	0.0051	-2.02
Tfrc	transferrin receptor	ENSRNOG00000001766	-1.01	0.0179	-2.02
Rerg	RAS-like, estrogen-regulated, growth-inhibitor	ENSRNOG00000027592	-1.01	0.0318	-2.02
Ptprz1	protein tyrosine phosphatase, receptor-type, Z polypeptide 1	ENSRNOG00000006030	-1.02	0.0047	-2.02
Fitm2	fat storage-inducing transmembrane protein 2	ENSRNOG00000027434	-1.02	0.0222	-2.02
Dock2	dedicator of cytokinesis 2	ENSRNOG00000006932	-1.02	0.0233	-2.03
Pgpep1	pyroglutamyl-peptidase I	ENSRNOG00000019639	-1.02	0.0153	-2.03
Nudt12	nudix (nucleoside diphosphate linked moiety X)-type motif 12	ENSRNOG00000022576	-1.02	0.0398	-2.03
Zfp426	zinc finger protein 266	ENSRNOG00000033624	-1.03	0.0178	-2.04
Jph2	junctophilin 2	ENSRNOG00000008170	-1.03	0.0249	-2.04
Trak2	trafficking protein, kinesin binding 2	ENSRNOG00000010881	-1.03	0.0146	-2.04
Fgf6	fibroblast growth factor 6	ENSRNOG00000019836	-1.03	0.0257	-2.04
Pkia	protein kinase (cAMP-dependent, catalytic) inhibitor alpha	ENSRNOG00000012095	-1.03	0.0246	-2.04
Klhl31	kelch-like family member 31	ENSRNOG00000006224	-1.03	0.0382	-2.05

Gene Symbol	Description	Ensembl ID	fold change log2	p value	fold change
Tbc1d31	TBC1 domain family, member 31	ENSRNOG00000006103	-1.03	0.0441	-2.05
Dpp4	dipeptidylpeptidase 4	ENSRNOG00000030763	-1.03	0.0236	-2.05
Fras1	Fraser extracellular matrix complex subunit 1	ENSRNOG00000002053	-1.04	0.0323	-2.05
Nceh1	neutral cholesterol ester hydrolase 1	ENSRNOG00000013313	-1.04	0.0095	-2.06
Nfam1	NFAT activating protein with ITAM motif 1	ENSRNOG00000022975	-1.04	0.0044	-2.06
Tmem150c	transmembrane protein 150C	ENSRNOG00000002258	-1.04	0.0257	-2.06
Ghr	growth hormone receptor	ENSRNOG00000015654	-1.05	0.0155	-2.07
Tpmt	thiopurine S-methyltransferase	ENSRNOG00000016468	-1.05	0.0142	-2.07
MGC108823	similar to interferon-inducible GTPase	ENSRNOG00000019542	-1.05	0.0280	-2.07
Col6a3	collagen, type VI, alpha 3	ENSRNOG00000019648	-1.05	0.0142	-2.07
Ccna1	cyclin A1	ENSRNOG00000014052	-1.06	0.0108	-2.08
Cxxc5	CXXC finger protein 5	ENSRNOG00000032878	-1.06	0.0037	-2.08
Ppic	peptidylprolyl isomerase C	ENSRNOG00000017416	-1.06	0.0332	-2.08
Rtn4	reticulon 4	ENSRNOG00000004621	-1.06	0.0312	-2.08
Nkiras1	NFKB inhibitor interacting Ras-like 1	ENSRNOG00000008565	-1.06	0.0043	-2.08
Hmcn2	hemicentin 2	ENSRNOG00000008820	-1.06	0.0042	-2.09
Dmrt2	doublesex and mab-3 related transcription factor 2	ENSRNOG00000016301	-1.06	0.0131	-2.09
Nr0b2	nuclear receptor subfamily 0, group B, member 2	ENSRNOG00000007229	-1.07	0.0177	-2.09
P2rx6	purinergic receptor P2X, ligand-gated ion channel, 6	ENSRNOG00000001873	-1.07	0.0167	-2.09
Mchr1	melanin-concentrating hormone receptor 1	ENSRNOG00000018895	-1.07	0.0092	-2.10
Entpd5	ectonucleoside triphosphate diphosphohydrolase 5	ENSRNOG00000033206	-1.07	0.0303	-2.10
Fam13a	family with sequence similarity 13, member A	ENSRNOG00000007947	-1.07	0.0224	-2.10
D2hgdh	D-2-hydroxyglutarate dehydrogenase	ENSRNOG00000019012	-1.07	0.0291	-2.10
Fgf2	fibroblast growth factor 2	ENSRNOG00000017392	-1.07	0.0187	-2.10
Tmc7	transmembrane channel-like 7	ENSRNOG00000016679	-1.07	0.0066	-2.11

Gene Symbol	Description	Ensembl ID	fold change log2	p value	fold change
Wnt5a	wingless-type MMTV integration site family, member 5A	ENSRNOG00000015618	-1.08	0.0053	-2.11
Rcan2	regulator of calcineurin 2	ENSRNOG00000010350	-1.08	0.0043	-2.11
Serpinf1	serpin peptidase inhibitor, clade F (alpha-2 antiplasmin, pigment epithelium derived factor), member 1	ENSRNOG00000003172	-1.08	0.0494	-2.11
Actr3b	ARP3 actin-related protein 3 homolog B (yeast)	ENSRNOG00000031855	-1.08	0.0172	-2.12
Calm1	calmodulin 1	ENSRNOG00000016770	-1.08	0.0323	-2.12
Dzip1	DAZ interacting zinc finger protein 1	ENSRNOG00000010311	-1.08	0.0282	-2.12
Coq3	coenzyme Q3 methyltransferase	ENSRNOG00000009974	-1.08	0.0178	-2.12
Rhobtb3	Rho-related BTB domain containing 3	ENSRNOG00000012414	-1.10	0.0050	-2.14
Hyal1	hyaluronoglucosaminidase 1	ENSRNOG00000015858	-1.10	0.0153	-2.15
Clec11a	C-type lectin domain family 11, member A	ENSRNOG00000019138	-1.11	0.0201	-2.16
Zim1	zinc finger, imprinted 1	ENSRNOG00000015071	-1.11	0.0248	-2.16
Epdr1	ependymin related 1	ENSRNOG00000018989	-1.11	0.0272	-2.16
Oplah	5-oxoprolinase (ATP-hydrolysing)	ENSRNOG00000011781	-1.11	0.0342	-2.16
Kcnq1	potassium voltage-gated channel, KQT-like subfamily, member 1	ENSRNOG00000020532	-1.12	0.0179	-2.17
Wls	wntless Wnt ligand secretion mediator	ENSRNOG00000036816	-1.12	0.0249	-2.17
Kif24	kinesin family member 24	ENSRNOG00000012735	-1.12	0.0055	-2.17
Cyp2j4	cytochrome P450, family 2, subfamily j, polypeptide 4	ENSRNOG00000031004	-1.12	0.0192	-2.18
Tesk2	testis-specific kinase 2	ENSRNOG00000017282	-1.13	0.0054	-2.18
Acsl6	acyl-CoA synthetase long-chain family member 6	ENSRNOG00000026745	-1.13	0.0462	-2.18
Fbxw7	F-box and WD repeat domain containing 7, E3 ubiquitin protein ligase	ENSRNOG00000010889	-1.13	0.0042	-2.19
Mamstr	MEF2 activating motif and SAP domain containing transcriptional regulator	ENSRNOG00000024580	-1.13	0.0483	-2.19
Bcs1l	BC1 (ubiquinol-cytochrome c reductase) synthesis-like	ENSRNOG00000016754	-1.13	0.0203	-2.19
Sobp	sine oculis-binding protein homolog (Drosophila)	ENSRNOG00000000316	-1.13	0.0164	-2.19
Armcx3	armadillo repeat containing, X-linked 3	ENSRNOG00000025730	-1.13	0.0314	-2.19
RGD1560334	similar to Myosin light chain 1 slow a	ENSRNOG00000003860	-1.13	0.0037	-2.19

Gene Symbol	Description	Ensembl ID	fold change log2	p value	fold change
Fn1	fibronectin 1	ENSRNOG00000014288	-1.13	0.0223	-2.19
Cc2d2a	coiled-coil and C2 domain containing 2A	ENSRNOG00000005106	-1.14	0.0067	-2.20
Rps6ka2	ribosomal protein S6 kinase polypeptide 2	ENSRNOG00000013194	-1.14	0.0342	-2.20
C1qtnf3	C1q and tumor necrosis factor related protein 3	ENSRNOG00000018570	-1.14	0.0133	-2.21
Ddah2	dimethylarginine dimethylaminohydrolase 2	ENSRNOG00000000842	-1.14	0.0155	-2.21
Ccnd1	cyclin D1	ENSRNOG00000020918	-1.14	0.0233	-2.21
Ndufs1	NADH dehydrogenase (ubiquinone) Fe-S protein 1	ENSRNOG00000011849	-1.14	0.0151	-2.21
Tmtc4	transmembrane and tetratricopeptide repeat containing 4	ENSRNOG00000014310	-1.14	0.0044	-2.21
Klhl13	kelch-like family member 13	ENSRNOG00000014029	-1.15	0.0131	-2.21
Sv2b	synaptic vesicle glycoprotein 2b	ENSRNOG00000011160	-1.15	0.0118	-2.22
Rilp	Rab interacting lysosomal protein	ENSRNOG00000003784	-1.15	0.0146	-2.23
Klhl23	kelch-like family member 23	ENSRNOG00000007981	-1.16	0.0133	-2.23
Taco1	translational activator of mitochondrially encoded cytochrome c oxidase I	ENSRNOG00000008405	-1.16	0.0129	-2.24
Tppp	tubulin polymerization promoting protein	ENSRNOG00000028261	-1.17	0.0459	-2.25
Akr1e2	aldo-keto reductase family 1, member E2	ENSRNOG00000017165	-1.17	0.0266	-2.25
Pcolce	procollagen C-endopeptidase enhancer	ENSRNOG00000025001	-1.17	0.0117	-2.25
Pde4a	phosphodiesterase 4A, cAMP-specific	ENSRNOG00000020828	-1.17	0.0398	-2.26
Plekha6	pleckstrin homology domain containing, family A member 6	ENSRNOG00000002907	-1.18	0.0063	-2.26
Fstl1	follistatin-like 1	ENSRNOG00000002746	-1.18	0.0241	-2.26
Lgi2	leucine-rich repeat LGI family, member 2	ENSRNOG00000003887	-1.18	0.0085	-2.27
RGD1311744	similar to RIKEN cDNA 5830475I06	ENSRNOG00000005960	-1.18	0.0092	-2.27
Schip1	schwannomin interacting protein 1	ENSRNOG00000009276	-1.18	0.0223	-2.27
Fam161b	family with sequence similarity 161, member B	ENSRNOG00000011112	-1.19	0.0089	-2.28
Dnajc28	DnaJ (Hsp40) homolog, subfamily C, member 28	ENSRNOG00000002026	-1.19	0.0342	-2.28
Tbck	TBC1 domain containing kinase	ENSRNOG00000011454	-1.19	0.0469	-2.28

Gene Symbol	Description	Ensembl ID	fold change log2	p value	fold change
Atp1b4	ATPase, (Na+)/K+ transporting, beta 4 polypeptide	ENSRNOG00000007059	-1.19	0.0178	-2.28
Tnfsf10	tumor necrosis factor (ligand) superfamily, member 10	ENSRNOG00000013269	-1.19	0.0125	-2.28
Cit	citron rho-interacting serine/threonine kinase	ENSRNOG00000001143	-1.19	0.0066	-2.29
Scube2	signal peptide, CUB domain, EGF-like 2	ENSRNOG00000013123	-1.20	0.0131	-2.30
Tp53inp2	tumor protein p53 inducible nuclear protein 2	ENSRNOG00000018225	-1.20	0.0166	-2.30
Smoc2	SPARC related modular calcium binding 2	ENSRNOG00000014166	-1.20	0.0136	-2.31
Opcml	opioid binding protein/cell adhesion molecule-like	ENSRNOG00000023809	-1.21	0.0149	-2.31
Dhrs11	dehydrogenase/reductase (SDR family) member 11	ENSRNOG00000027891	-1.22	0.0183	-2.33
Slc25a30	solute carrier family 25, member 30	ENSRNOG00000001052	-1.23	0.0142	-2.34
Palld	palladin, cytoskeletal associated protein	ENSRNOG00000010107	-1.23	0.0217	-2.34
Lgals1	lectin, galactoside-binding, soluble, 1	ENSRNOG00000009884	-1.23	0.0042	-2.35
Reep1	receptor accessory protein 1	ENSRNOG00000008481	-1.23	0.0113	-2.35
Abi3bp	ABI family, member 3 (NESH) binding protein	ENSRNOG00000001627	-1.24	0.0307	-2.36
Mkks	McKusick-Kaufman syndrome	ENSRNOG00000006705	-1.24	0.0341	-2.36
Armcx6	armadillo repeat containing, X-linked 6	ENSRNOG00000037707	-1.25	0.0073	-2.37
Col11a1	collagen, type XI, alpha 1	ENSRNOG00000023148	-1.25	0.0225	-2.38
Osgin2	oxidative stress induced growth inhibitor family member 2	ENSRNOG00000009358	-1.25	0.0119	-2.38
Grb14	growth factor receptor bound protein 14	ENSRNOG00000031396	-1.25	0.0104	-2.39
Cd248	CD248 molecule, endosialin	ENSRNOG00000020197	-1.26	0.0066	-2.39
Fsd1l	fibronectin type III and SPRY domain containing 1-like	ENSRNOG00000017823	-1.26	0.0210	-2.39
Prkg1	protein kinase, cGMP-dependent, type 1	ENSRNOG00000033641	-1.27	0.0172	-2.40
Myh7b	myosin, heavy chain 7B, cardiac muscle, beta	ENSRNOG00000018997	-1.27	0.0241	-2.41
Ncf1	neutrophil cytosolic factor 1	ENSRNOG00000001480	-1.27	0.0178	-2.42
RGD1311863	similar to RIKEN cDNA 2410127L17	ENSRNOG00000012872	-1.27	0.0229	-2.42
Oxnad1	oxidoreductase NAD-binding domain containing 1	ENSRNOG00000019760	-1.28	0.0138	-2.42

Gene Symbol	Description	Ensembl ID	fold change log2	p value	fold change
Wfdc1	WAP four-disulfide core domain 1	ENSRNOG00000015904	-1.28	0.0068	-2.43
Pi16	peptidase inhibitor 16	ENSRNOG00000000525	-1.28	0.0209	-2.43
Aqp7	aquaporin 7	ENSRNOG00000009686	-1.28	0.0317	-2.43
Sspn	sarcospan	ENSRNOG00000001807	-1.28	0.0194	-2.43
Tppp3	tubulin polymerization-promoting protein family member 3	ENSRNOG00000016890	-1.29	0.0163	-2.44
Pecr	peroxisomal trans-2-enoyl-CoA reductase	ENSRNOG00000015809	-1.29	0.0390	-2.44
Acot13	acyl-CoA thioesterase 13	ENSRNOG00000018415	-1.29	0.0445	-2.45
Pstk	phosphoseryl-tRNA kinase	ENSRNOG00000020605	-1.30	0.0459	-2.46
Efr3b	EFR3 homolog B (S. cerevisiae)	ENSRNOG00000012950	-1.30	0.0159	-2.46
Dusp19	dual specificity phosphatase 19	ENSRNOG00000008868	-1.30	0.0118	-2.47
Smtn	smoothelin	ENSRNOG00000019451	-1.31	0.0026	-2.47
Dffa	DNA fragmentation factor, alpha subunit	ENSRNOG00000013603	-1.31	0.0172	-2.48
Lynx1	Ly6/neurotoxin 1	ENSRNOG00000006086	-1.31	0.0216	-2.48
Fam216b	family with sequence similarity 216, member B	ENSRNOG00000021943	-1.31	0.0063	-2.48
Slc47a1	solute carrier family 47 (multidrug and toxin extrusion), member 1	ENSRNOG00000002355	-1.32	0.0213	-2.49
Nudt8	nudix (nucleoside diphosphate linked moiety X)-type motif 8	ENSRNOG00000017955	-1.32	0.0115	-2.49
Capn6	calpain 6	ENSRNOG00000004882	-1.32	0.0101	-2.49
Actc1	actin, alpha, cardiac muscle 1	ENSRNOG00000008536	-1.32	0.0131	-2.50
Ssc5d	scavenger receptor cysteine rich family, 5 domains	ENSRNOG00000016687	-1.32	0.0023	-2.50
Bgn	biglycan	ENSRNOG00000017440	-1.33	0.0371	-2.51
Abcd2	ATP-binding cassette, subfamily D (ALD), member 2	ENSRNOG00000015538	-1.34	0.0355	-2.53
Igdcc4	immunoglobulin superfamily, DCC subclass, member 4	ENSRNOG00000033496	-1.35	0.0123	-2.54
Cilp	cartilage intermediate layer protein, nucleotide pyrophosphohydrolase	ENSRNOG00000029911	-1.35	0.0133	-2.55
Scn1a	sodium channel, voltage-gated, type I, alpha subunit	ENSRNOG00000005989	-1.35	0.0275	-2.55
Dynll1	dynein light chain LC8-type 1	ENSRNOG00000011222	-1.36	0.0229	-2.56

Gene Symbol	Description	Ensembl ID	fold change log2	p value	fold change
RragB	Ras-related GTP binding B	ENSRNOG00000003160	-1.37	0.0320	-2.58
Ebpl	emopamil binding protein-like	ENSRNOG00000014659	-1.37	0.0023	-2.59
Slc16a3	solute carrier family 16 (monocarboxylate transporter), member 3	ENSRNOG00000036677	-1.38	0.0095	-2.59
Inpp5j	inositol polyphosphate-5-phosphatase J	ENSRNOG00000019361	-1.38	0.0023	-2.60
Tmem25	transmembrane protein 25	ENSRNOG00000014218	-1.38	0.0030	-2.60
Pik3ap1	phosphoinositide-3-kinase adaptor protein 1	ENSRNOG00000013309	-1.38	0.0296	-2.60
Carns1	carnosine synthase 1	ENSRNOG00000018603	-1.39	0.0066	-2.62
Bub1	BUB1 mitotic checkpoint serine/threonine kinase	ENSRNOG00000032778	-1.39	0.0208	-2.63
Nt5c1a	5'-nucleotidase, cytosolic IA	ENSRNOG00000015283	-1.40	0.0279	-2.63
Ppara	peroxisome proliferator activated receptor alpha	ENSRNOG00000021463	-1.40	0.0133	-2.64
Clcn4	chloride channel, voltage-sensitive 4	ENSRNOG00000003533	-1.40	0.0407	-2.64
Mmp15	matrix metallopeptidase 15	ENSRNOG00000012622	-1.41	0.0071	-2.67
Plekhh3	pleckstrin homology domain containing, family H (with MyTH4 domain) member 3	ENSRNOG00000020238	-1.42	0.0021	-2.68
Fndc1	fibronectin type III domain containing 1	ENSRNOG00000030210	-1.44	0.0241	-2.72
Zfp385a	zinc finger protein 385A	ENSRNOG00000036833	-1.46	0.0223	-2.75
Myo3b	myosin IIIB	ENSRNOG00000030022	-1.48	0.0310	-2.78
Ccdc80	coiled-coil domain containing 80	ENSRNOG00000002052	-1.48	0.0225	-2.79
Cnst	consortin, connexin sorting protein	ENSRNOG00000002710	-1.48	0.0142	-2.79
Postn	periostin, osteoblast specific factor	ENSRNOG00000012660	-1.54	0.0129	-2.91
Mum1l1	melanoma associated antigen (mutated) 1-like 1	ENSRNOG00000030475	-1.54	0.0035	-2.91
Asb15	ankyrin repeat and SOCS box containing 15	ENSRNOG00000006365	-1.55	0.0378	-2.93
Dbp	D site of albumin promoter (albumin D-box) binding protein	ENSRNOG00000021027	-1.56	0.0096	-2.94
Sfrp2	secreted frizzled-related protein 2	ENSRNOG00000009465	-1.56	0.0023	-2.95
Scd1	stearoyl-Coenzyme A desaturase 1	ENSRNOG00000013552	-1.57	0.0324	-2.96
Esr1	estrogen receptor 1	ENSRNOG00000019358	-1.57	0.0056	-2.96

Gene Symbol	Description	Ensembl ID	fold change log2	p value	fold change
Sesn3	sestrin 3	ENSRNOG00000008173	-1.57	0.0043	-2.97
Sema6c	sema domain, transmembrane domain (TM), and cytoplasmic domain, (semaphorin) 6C	ENSRNOG00000021101	-1.60	0.0225	-3.04
Rcor2	REST corepressor 2	ENSRNOG00000021183	-1.60	0.0023	-3.04
Slco5a1	solute carrier organic anion transporter family, member 5A1	ENSRNOG00000008966	-1.61	0.0112	-3.05
Agbl1	ATP/GTP binding protein-like 1	ENSRNOG00000022610	-1.61	0.0327	-3.06
G0s2	G0/G1switch 2	ENSRNOG00000006019	-1.62	0.0018	-3.08
Abhd1	abhydrolase domain containing 1	ENSRNOG00000025689	-1.63	0.0072	-3.10
Ppargc1b	peroxisome proliferator-activated receptor gamma, coactivator 1 beta	ENSRNOG00000017503	-1.63	0.0023	-3.10
Sypl2	synaptophysin-like 2	ENSRNOG00000019780	-1.65	0.0365	-3.14
Stat1	signal transducer and activator of transcription 1	ENSRNOG00000014079	-1.65	0.0085	-3.14
Arhgef9	Cdc42 guanine nucleotide exchange factor (GEF) 9	ENSRNOG00000007733	-1.65	0.0039	-3.14
Gpd1	glycerol-3-phosphate dehydrogenase 1 (soluble)	ENSRNOG00000019213	-1.66	0.0068	-3.16
Col14a1	collagen, type XIV, alpha 1	ENSRNOG00000026415	-1.66	0.0240	-3.17
Rasgrf1	RAS protein-specific guanine nucleotide-releasing factor 1	ENSRNOG00000014025	-1.69	0.0017	-3.22
Crabp1	cellular retinoic acid binding protein 1	ENSRNOG00000023633	-1.71	0.0133	-3.26
Cdc42ep2	CDC42 effector protein (Rho GTPase binding) 2	ENSRNOG00000020904	-1.71	0.0101	-3.27
Hpdl	4-hydroxyphenylpyruvate dioxygenase-like	ENSRNOG00000018143	-1.71	0.0131	-3.27
Angptl2	angiopoietin-like 2	ENSRNOG00000016678	-1.73	0.0057	-3.32
Sacs	sacsin molecular chaperone	ENSRNOG00000014509	-1.73	0.0017	-3.32
Akr1b10	aldo-keto reductase family 1, member B10 (aldose reductase)	ENSRNOG00000027433	-1.74	0.0039	-3.35
Otub2	OTU deubiquitinase, ubiquitin aldehyde binding 2	ENSRNOG00000009117	-1.75	0.0077	-3.35
Pdp1	pyruvate dehyrogenase phosphatase catalytic subunit 1	ENSRNOG00000016180	-1.75	0.0023	-3.37
Nqo2	NAD(P)H dehydrogenase, quinone 2	ENSRNOG00000017820	-1.77	0.0125	-3.42
Adamts8	ADAM metallopeptidase with thrombospondin type 1 motif, 8	ENSRNOG00000005574	-1.80	0.0342	-3.47
Trim7	tripartite motif-containing 7	ENSRNOG00000002469	-1.80	0.0019	-3.48

Gene Symbol	Description	Ensembl ID	fold change log2	p value	fold change
Fsd2	fibronectin type III and SPRY domain containing 2	ENSRNOG00000019278	-1.80	0.0202	-3.49
Plcd4	phospholipase C, delta 4	ENSRNOG00000016361	-1.83	0.0142	-3.55
Cdh23	cadherin-related 23	ENSRNOG00000033087	-1.86	0.0447	-3.62
Myl6b	myosin, light chain 6B, alkali, smooth muscle and non-muscle	ENSRNOG00000028837	-1.89	0.0017	-3.71
Itgb1bp2	integrin beta 1 binding protein 2	ENSRNOG00000003596	-1.90	0.0115	-3.72
Sel1l3	sel-1 suppressor of lin-12-like 3 (C. elegans)	ENSRNOG00000004932	-1.90	0.0078	-3.73
Gamt	guanidinoacetate N-methyltransferase	ENSRNOG00000024577	-1.91	0.0035	-3.76
Col1a2	collagen, type I, alpha 2	ENSRNOG00000011292	-1.96	0.0066	-3.88
Scn4b	sodium channel, voltage-gated, type IV, beta subunit	ENSRNOG00000026679	-1.96	0.0246	-3.89
Chodl	chondrolectin	ENSRNOG00000001915	-1.98	0.0055	-3.96
Pfkfb1	6-phosphofructo-2-kinase/fructose-2,6-biphosphatase 1	ENSRNOG00000000165	-2.01	0.0321	-4.04
Lum	lumican	ENSRNOG00000004610	-2.13	0.0039	-4.38
Gadl1	glutamate decarboxylase-like 1	ENSRNOG00000013221	-2.13	0.0088	-4.39
Itm2a	integral membrane protein 2A	ENSRNOG00000002365	-2.14	0.0032	-4.40
Grm4	glutamate receptor, metabotropic 4	ENSRNOG00000000487	-2.15	0.0017	-4.45
Clcn1	chloride channel, voltage-sensitive 1	ENSRNOG00000016917	-2.17	0.0323	-4.50
P2ry1	purinergic receptor P2Y, G-protein coupled, 1	ENSRNOG00000014232	-2.28	0.0146	-4.85
Col5a1	collagen, type V, alpha 1	ENSRNOG00000008749	-2.28	0.0049	-4.85
Smco1	single-pass membrane protein with coiled-coil domains 1	ENSRNOG00000024960	-2.30	0.0142	-4.91
Tet1	tet methylcytosine dioxygenase 1	ENSRNOG00000000277	-2.30	0.0023	-4.92
Ky	kyphoscoliosis peptidase	ENSRNOG00000008210	-2.32	0.0088	-5.00
Igfbp5	insulin-like growth factor binding protein 5	ENSRNOG00000017206	-2.33	0.0023	-5.04
Dok5	docking protein 5	ENSRNOG00000013196	-2.35	0.0223	-5.08
Cpa1	carboxypeptidase A1 (pancreatic)	ENSRNOG00000010725	-2.38	0.0016	-5.22
Kcnj13	potassium inwardly-rectifying channel, subfamily J, member 13	ENSRNOG00000016057	-2.41	0.0420	-5.30

Gene Symbol	Description	Ensembl ID	fold change log2	p value	fold change
Kera	keratocan	ENSRNOG00000004635	-2.46	0.0471	-5.51
Col1a1	collagen, type I, alpha 1	ENSRNOG00000003897	-2.53	0.0035	-5.77
Sfrp4	secreted frizzled-related protein 4	ENSRNOG00000018893	-2.58	0.0163	-5.98
Mfap4	microfibril-associated glycoprotein 4-like	ENSRNOG00000002382	-2.58	0.0030	-5.98
Rab3a	RAB3A, member RAS oncogene family	ENSRNOG00000019433	-2.60	0.0023	-6.08
Map2k6	mitogen-activated protein kinase kinase 6	ENSRNOG00000004437	-2.64	0.0017	-6.24
Mlf1	myeloid leukemia factor 1	ENSRNOG00000012827	-2.77	0.0063	-6.83
Pik3c2g	phosphatidylinositol-4-phosphate 3-kinase, catalytic subunit type 2 gamma	ENSRNOG00000034228	-2.80	0.0100	-6.97
Col3a1	collagen, type III, alpha 1	ENSRNOG00000003357	-3.09	0.0035	-8.53
Nrep	neuronal regeneration related protein	ENSRNOG00000020467	-3.25	0.0017	-9.52
RGD1565323	similar to OTTMUSP00000000621	ENSRNOG00000021789	-3.84	0.0201	-14.33

Table Supplementary 2 Functional classification of genes differentially expressed in cardiac cachexia

Go Module	GOTERM	GO database	GOID	p value term
**Structural Genes**	Collagen biosynthesis	REACTOME	5417884	3.5 E-4
	Regulation of myotube differentiation	GO: BiologicalProcess	10830	2.2 E-2
	Extracellular matrix organization	REACTOME	5417076	6.0 E-7
	Muscle contraction	GO: BiologicalProcess	6936	4.3 E-2
**Metabolic processes**	Regulation of lipid storage	GO: BiologicalProcess	10883	6.8 E-3
	The citric acid cycle and respiratory electron transport	REACTOME	5416705	6.0 E-4
	Metabolism of amino acids and derivatives	REACTOME	5416707	1.9 E-4
	Glycerolipid metabolic process	GO: BiologicalProcess	46486	4.4 E-4
	ATP metabolic process	GO: BiologicalProcess	46034	3.6 E-2
	Carbohydrate metabolic process	GO: BiologicalProcess	5975	4.4 E-6
**Cell Proteolytic systems and cell death**	Ubiquitin-Proteasome	KEGG	3050	3.7 E-8
	p53 signaling pathway	KEGG	4115	5.7 E-3
	Senescence and Autophagy	WikiPathways	1267	2.9 E-2
	Apoptosis	REACTOME	5416750	5.5 E-7
	Positive regulation of cell death	GO: BiologicalProcess	10942	1.5 E-6
	Positive regulation of peptidase activity	GO: BiologicalProcess	10952	1.3 E-4
**Muscle growth and differentiation**	IGF signaling pathway	GO: BiologicalProcess	48009	3.1 E-3
	TGF beta signaling pathway	GO: BiologicalProcess	7179	7.9 E-3
	PI3K-Akt signaling pathway	KEGG	4151	4.0 E-9
	Positive regulation of cell adhesion	GO: BiologicalProcess	45785	2.6 E-2
	Negative regulation of cell migration	GO: BiologicalProcess	30336	1.7 E-2
	Signaling by Wnt	REACTOME	5417230	4.2 E-2
	MAPK signaling pathway	KEGG	4010	3.0 E-3
	Negative regulation of cell proliferation	GO: BiologicalProcess	8285	2.7 E-6
**Cytokines signaling pathway**	Negative regulation of chemokine production	GO: BiologicalProcess	32682	1.6 E-2
	Regulation of I-kappaB kinase/NF-kappaB signaling	GO: BiologicalProcess	43122	3.6 E-2
**Ion regulation**	Positive regulation of ion transport	GO: BiologicalProcess	43270	2.4 E-2
	Cellular calcium ion homeostasis	GO: BiologicalProcess	6874	3.0 E-3
**Others**	positive regulation of angiogenesis	GO: BiologicalProcess	45766	1.7 E-2
	protein kinase B signaling	GO: BiologicalProcess	43491	1.9 E-2

Table Supplementary 2 Functional classification of genes differentially expressed in cardiac cachexia

Go Module	% associated genes	Nr.genes	Associated genes found
**Structural Genes**	24.074074	13	[Bmp1, Col11a1, Col14a1, Col1a1, Col1a2, Col3a1, Col5a1, Col6a1, Col6a2, Colgalt2
	18.965517	11	[Cav3, Cdon, Hif1an, Id3, Igfbp3, Mamstr, Myocd, Naca, Ppapdc3, Sik1, Smyd1]
	14.230769	37	[Bgn, Bmp1, Col11a1, Col14a1, Col1a1, Col1a2, Col3a1, Col5a1, Col6a1, Col6a2,
	10.900474	23	[Actc1, Ank2, Casq1, Casq2, Cav3, Chrna1, Edn1, Ednrb, Kcnh2, Kcnma1, Kcnq1,
**Metabolic processes**	26.470589	9	[Fitm2, Itgav, Nfkbia, Osbpl11, Osbpl8, Pnpla2, Ppara, Ptpn2, Zc3h12a]
	13.953488	24	[Atp5c1, Atp5g1, Atp5k, Atp5o, Cyc1, Idh3g, Mpc2, Ndufa12, Ndufa2, Ndufa8,
	13.705584	27	[Ccbl1, Cdo1, Fah, Gamt, Gls2, Hibch, Hpd, Psma3, Psma4, Psma6, Psmb3,
	12.345679	30	[Acsl4, Acsl6, Bpnt1, Cav3, Chka, Cyp2e1, Dgkh, Fabp3, Fgf2, Fgf7, Fitm2, Gpd1,
	10.810811	24	[Abcb7, Abcd1, Abcd3, Actc1, Ak2, Atf7ip, Atp1b1, Atp5c1, Atp5g1, Atp5g3, Atp5k,
	10.032362	62	[Acadm, Acer2, Adcyap1r1, Akr1b3, Art3, Atp1b1, B3galnt2, C1qtnf2, C1qtnf3, Cds2,
**Cell Proteolytic systems and cell death**	35.555557	16	[Psma3, Psma4, Psma6, Psmb3, Psmc1, Psmc4, Psmc6, Psmd1, Psmd11,
	18.84058	13	[Atr, Bid, Ccnd1, Ccnd2, Cdk6, Cdkn1a, Gadd45b, Igfbp3, Pmaip1, Ppm1d, Serpine1,
	18.333334	11	[Cdkn1a, Col1a1, Creg1, Fn1, Igf1r, Igfbp5, Il6st, Lamp2, Plat, Rnasel, Serpine1]
	17.058823	29	[Arhgap10, Bcl2l1, Bcl2l11, Bid, Bmx, Dapk1, Dffa, Dnm1l, Dynll1, Kpna1, Pmaip1,
	11.898734	47	[Acer2, Adm, Aldh1a2, Axin2, Bcl2l1, Bcl2l11, Bid, Brms1, Casp12, Cd248, Cd24a,
	16.17647	22	[Acer2, Adrm1, Anp32b, Bcl2l11, Bid, Casp12, Cradd, Cyr61, Fn1, Hspd1, Ift57,
**Muscle growth and differentiation**	29.032259	9	[Atxn1, Cilp, Ghr, Igf1r, Igfbp3, Igfbp5, Inppl1, Irs1, Phip]
	14.285714	18	[Cav3, Ccl2, Cited2, Col1a2, Col3a1, Dusp22, Fos, Furin, Gcnt2, Itga8, Lemd3,
	13.802817	49	[Angpt2, Bcl2l1, Bcl2l11, Ccnd1, Ccnd2, Cdk6, Cdkn1a, Col11a1, Col1a1, Col1a2,
	12.582782	19	[Abi3bp, Ccdc80, Cd24a, Cd63, Cdk6, Cited2, Cyr61, Gcnt2, Gpr56, Hyal1, Itga5,
	12.578616	20	[Abhd2, Adarb1, Angpt2, Cd63, Cited2, Col3a1, Dach1, Dll4, Fgf2, Gnrh1, Gpr56,
	11.167513	22	[Calm1, Cul1, Gng7, Ppp2r5b, Psma3, Psma4, Psma6, Psmb3, Psmc1, Psmc4,
	11.417323	29	[Cacna2d1, Cacna2d3, Cacnb4, Cacng6, Dusp10, Elk1, Fgf11, Fgf2, Fgf6, Fgf7,
	10.575139	57	[Adarb1, Adm, Aldh1a2, Axin2, Btg2, Cblb, Cd24a, Cdk6, Cdkn1a, Ctsl, Cxadr,
**Cytokines signaling pathway**	50	5	[C1qtnf3, F2rl1, Gstp1, Klf4, Slc37a4]
	11.904762	20	[C1qtnf3, Cxxc5, Esr1, F2rl1, Gstp1, Hmox1, Il18, Lgals1, Lgals9, Litaf, Myd88, Nfkbia,
**Ion regulation**	12.269938	20	[Acsl6, Adcyap1r1, Ank2, Creb3, Hbp1, Kcnh2, Kcnq1, Lgals3, Map2k6, Mchr1,
	10.847458	32	[Adcyap1r1, Adm, Ank2, Cacnb4, Calcr, Casq1, Casq2, Cav3, Ccdc109b, Ccl2,
**Others**	14.545455	16	[Adm, Angpt2, Chil1, Fgf2, Gata6, Hmox1, Hyal1, Mir23a, Mir23b, Mir27a, Pgf,
	13.333333	18	[C1qtnf3, Cav3, Chil1, Clcf1, Fgf2, Gcnt2, Igf1r, Igfbp5, Inpp5f, Irs1, Klf4, Meis3,

Table Supplementary 2 Functional classification of genes differentially expressed in cardiac cachexia

Go Module	Associated genes found
**Structural Genes**	Leprel1, Leprel2, Pcolce]
	Colgalt2, Ctsd, Ctsl, Fgf2, Fn1, Furin, Hspg2, Itga5, Itga8, Itgav, Itgb6, Lama4, Leprel1, Leprel2, Lox, Ltbp3, Ltbp4, Lum,
	Map2k6, Mkks, Myh7b, Myl6b, Myocd, Ncf1, Nos1, Ppp1r13l, Prkg1, Scn4b, Smad7, Vegfb]
**Metabolic processes**	
	Ndufb3, Ndufb5, Ndufb6, Ndufb7, Ndufs1, Ndufs3, Ndufs5, Ndufs8, Ndufv1, Pdk4, Pdp1, Sdhd, Slc16a3, Uqcrc1]
	Psmc1, Psmc4, Psmc6, Psmd1, Psmd11, Psmd12, Psmd13, Psmd14, Psmd2, Psmd3, Psmd8, Psme2, Sat1, Slc25a2,
	H6pd, Il6st, Inpp5f, Inpp5j, Inppl1, Mecp2, Mppe1, Pdgfa, Pdgfrb, Pi4k2a, Pigq, Pik3c2a, Pik3c2g, Pip4k2b, Plcd4, Pnpla2,
	Atp5o, Blm, Carns1, Entpd5, Msh3, Myc, Myh7b, Ndufs1, Pcyox1, Pms2, Rad54b, Rhobtb3, Wrn]
	Chil1, Chst7, Colgalt2, Coq3, Entpd5, Extl1, Foxo1, Fuom, Galc, Gcgr, Gcnt2, Gfpt2, Gpd1, H6pd, Hyal1, Hyal3, Igfbp3,
**Cell Proteolytic systems and cell death**	Psmd12, Psmd13, Psmd14, Psmd2, Psmd3, Psmd8, Psme2]
	Sesn1, Sesn3]
	Psma3, Psma4, Psma6, Psmb3, Psmc1, Psmc4, Psmc6, Psmd1, Psmd11, Psmd12, Psmd13, Psmd14, Psmd2, Psmd3,
	Cdk5, Cdkn1a, Cyr61, Dffa, Dnm1l, Efhc1, Elk1, Fndc1, Gadd45b, Gata6, Hspd1, Id3, Igfbp3, Il18, Ing3, Kcnma1, Klf11, Map2k6,
	Mmp14, Myc, Pcolce, Pmaip1, Psmd14, Psme2, S100a9, Sfrp2, Stat1, Tnfsf10, Uaca]
**Muscle growth and differentiation**	
	Ltbp3, Ltbp4, Mstn, Myocd, Nrep, Smad6, Smad7]
	Col3a1, Col5a1, Col6a1, Col6a2, Col6a3, Col6a6, Creb3, Efna1, Eif4ebp1, Fgf11, Fgf2, Fgf6, Fgf7, Fgf9, Fn1, Ghr, Gng7,
	Itgav, Lgals1, Plekha2, Prex1, Sdc4, Smad7, Smoc2, Wnt5a]
	Gtpbp4, Igfbp3, Igfbp5, Klf4, Osbpl8, Pfn2, Prkg1, Serpine1, Sfrp2]
	Psmc6, Psmd1, Psmd11, Psmd12, Psmd13, Psmd14, Psmd2, Psmd3, Psmd8, Psme2, Wls, Wnt5a]
	Fgf9, Fos, Gadd45b, Hspa2, Map2k6, Map3k2, Map3k6, Mapk10, Mecom, Mknk1, Myc, Nr4a1, Pdgfa, Pdgfrb, Pla2g4e,
	Dach1, Dll4, Efnb2, Fgf2, Gdf11, Gpr56, Gstp1, Gtpbp4, Hes1, Hmox1, Hyal1, Ift57, Igfbp3, Igfbp5, Inppl1, Klf11, Klf4, Lbx1,
**Cytokines signaling pathway**	
	Pik3ap1, Sqstm1, Stat1, Terf2ip, Tle1, Tlr3, Uaca, Ube2v1]
**Ion regulation**	Nos1, P2ry1, Pdgfrb, Rtn4, Scn4b, Serpine1, Sgk1, Slc36a2, Tmem132a, Trpc1]
	Cd24a, Cdh23, Edn1, Ednrb, Efhc1, Esr1, F2rl1, Fgf2, Il6st, Itgav, Jph2, Mchr1, Ndn, Nos1, Nucb2, P2ry1, Prkg1, S1pr1,
**Others**	Runx1, Sema5a, Serpine1, Sfrp2, Wnt5a]
	Osbpl8, Pdgfa, Phlda3, Sema5a, Sesn1, Sesn3]

Table Supplementary 2 Functional classification of genes differentially expressed in cardiac cachexia

Go Module	Associated genes found
**Structural Genes**	Mfap4, Mmp14, Mmp15, Mmp16, Mmp8, Nid2, Pcolce, Pdgfa, Sdc4]
**Metabolic processes**	Sms, Sqrdl]<br>Sik1, Slc37a4]<br>Igfbp5, Il6st, Inppl1, Irs1, Man2b2, Mdh1, Mecp2, Mlec, Mpdu1, Myc, P2ry1, Pak1ip1, Pdk4, Pfkfb1, Pgm5, Phka2, Phkg1, Pigq,
**Cell Proteolytic systems and cell death**	Psmd8, Psme2, Tnfsf10, Unc5b]<br>Myc, Nos1, Nr4a1, Nupr1, Phlda1, Phlda3, Pmaip1, Rps6ka2, Sfrp2, Sfrp4, Sik1, Tnfrsf12a, Tnfsf10, Trim35, Txnip, Uaca,
**Muscle growth and differentiation**	Igf1r, Il4ra, Irs1, Itga5, Itga8, Itgav, Itgb6, Lama4,<br>Prkaca, Rasgrf1, Rasgrp3, Rps6ka2]<br>Leprel1, Leprel2, Lrrc32, Mbd4, Mfn2, Mtss1, Myc, Myocd, Nos1, Npm1, Nupr1, Pmaip1, Ptpn14, Ptpn2, Rerg, Rnf139,
**Cytokines signaling pathway**	
**Ion regulation**	Slc37a4, Stc1, Sypl2, Trpc1]
**Others**	

Table Supplementary 2 Functional classification of genes differentially expressed in cardiac cachexia

Go Module	Associated genes found
Structural Genes	
Metabolic processes	Pmm1, Pomk, Ppara, Prkg1, Ptpn2, Ramp1, Rorc, Siae, Sik1, Slc35b4, Slc37a4, Slc3a2, Sorbs1, St8sia4, Tet1, Trak2, Vegfb]
Cell Proteolytic systems and cell death	Utp11l, Wnt5a]
Muscle growth and differentiation	Rps6ka2, Serpinf1, Sfrp2, Sfrp4, Smad6, Smarca2, Stat1, Tenc1, Tgif1, Trim35, Wfdc1, Wnt5a]
Cytokines signaling pathway	
Ion regulation	
Others	

Table Supplementary S3 Top upregulated genes in cardiac cachexia.
Ranked by a combination of p-value < 0.05 and fold change ≥ 5. [a] GO terms determined by Uniprot and AmiGO 2 databases.

Gene Symbol	Description	Ensembl ID	GO Biological Process[a]	Fold Change	p Value
Cyp2e1	Cytochrome P450, family 2, subfamily e, polypeptide 1	ENSRNOG00000012458	Metabolic and oxi-reduction process	49.57	0.0010
Mt1m	Metallothionein 1M	ENSRNOG00000028841	Metabolic process	8.18	0.0082
Fah	Fumarylacetoacetate hydrolase	ENSRNOG00000013223	Metabolic process	7.74	0.0023
Mt1	Metallothionein 1	ENSRNOG00000025764	Negative regulation of growth	7.42	0.0042
Pbld1	Phenazine biosynthesis-like protein domain containing 1	ENSRNOG00000000386	Biosynthetic process	6.99	0.0061
Cdkn1a	Cyclin-dependent kinase inhibitor 1A	ENSRNOG00000000521	Negative regulation of cell growth	6.29	0.0131
Serpine1	Serpin peptidase inhibitor, clade E, member 1	ENSRNOG00000001414	Response to hyperoxia	5.51	0.0172
Mt2A	Metallothionein 2A	ENSRNOG00000043098	Negative regulation of growth	5.31	0.0067

Table Supplementary S4 Top downregulated genes in cardiac cachexia

Gene Symbol	Description	Ensembl ID	GO Biological Process [a]	Fold Change	p Value
Ky	Kyphoscoliosis peptidase	ENSRNOG00000008210	Neuromuscular junction development	-5.00	0.0088
Igfbp5	Insulin-like growth factor binding protein 5	ENSRNOG00000017206	Skeletal muscle tissue growth	-5.04	0.0023
Dok5	Docking protein 5	ENSRNOG00000013196	Nervous system development	-5.08	0.0223
Cpa1	Carboxypeptidase A1 (pancreatic)	ENSRNOG00000010725	Proteolysis involved in cellular protein catabolic	-5.22	0.0016
Kcnj13	Potassium inwardly-rectifying channel, subfamily J, member 13	ENSRNOG00000016057	Ion transport	-5.30	0.0420
Kera	Keratocan	ENSRNOG00000004635		-5.51	0.0471
Col1a1	Collagen, type I, alpha 1	ENSRNOG00000003897	Collagen biosynthetic process and skeletal	-5.77	0.0035
Sfrp4	Secreted frizzled-related protein 4	ENSRNOG00000018893	Cell differentiation	-5.98	0.0163
Mfap4	Microfibril-associated glycoprotein 4-like	ENSRNOG00000002382	Signal transduction	-5.98	0.0030
Rab3a	RAB3A, member RAS oncogene family	ENSRNOG00000019433	Mitochondrion organization	-6.08	0.0023
Map2k6	Mitogen-activated protein kinase kinase 6	ENSRNOG00000004437	Positive regulation of apoptosis	-6.24	0.0017
Mlf1	Myeloid leukemia factor 1	ENSRNOG00000012827	Cell cycle arrest	-6.83	0.0063
Pik3c2g	Phosphatidylinositol-4-phosphate 3-kinase, catalytic subunit	ENSRNOG00000034228	Phosphatidylinositol phosphorylation	-6.97	0.0100
Col3a1	Collagen, type III, alpha 1	ENSRNOG00000003357	Collagen biosynthetic process	-8.53	0.0035
Nrep	Neuronal regeneration related protein	ENSRNOG00000020467	Regulation of transforming growth factor beta	-9.52	0.0017
RGD1565323	Similar to OTTMUSP00000000621	ENSRNOG00000021789	Protein phosphorylation	-14.33	0.0201

Ranked by a combination of p-value < 0.05 and fold change ≤ -5. [a] GO terms determined by Uniprot and AmiGO 2 databases.

Table Supplementary 5 Results of miRNA expression analysis using TaqMan Low Density Arrays

Target Name (ID LifeTech)	Cт Mean	ΔCт Mean	RQ	P-Value	Threshold
**mmu-miR-29b-4373288**	33.492	7.691	2.554	0.020	0.183
**mmu-miR-132-4373143**	29.414	3.612	2.261	0.032	0.133
**hsa-miR-27a#-002445**	30.662	5.971	2.218	0.018	0.118
**mmu-miR-337-5p-4395645**	31.056	5.255	1.975	0.030	0.115
**mmu-miR-434-3p-4395734**	27.236	1.435	1.936	0.001	0.142
**mmu-miR-539-4378103**	26.400	0.598	1.906	0.001	0.303
**mmu-miR-136-4395641**	29.777	3.976	1.877	0.024	0.152
**mmu-miR-210-4373089**	28.264	2.462	1.722	0.022	0.060
**mmu-miR-322-4378107**	29.039	3.238	1.705	0.008	0.177
**mmu-miR-331-3p-4373046**	27.517	1.716	1.583	0.012	0.109
**mmu-miR-376c-4395580**	30.819	5.018	1.575	0.006	0.222
**mmu-miR-29a-4395223**	24.473	-1.328	1.520	0.000	0.249
**mmu-miR-204-4373094**	30.342	4.541	1.504	0.009	0.160
**hsa-miR-30d#-002305**	31.446	6.756	0.625	0.040	0.043
**rno-miR-146B-002755**	28.542	3.852	0.585	0.016	0.160
**hsa-miR-214-000517**	28.545	3.854	0.583	0.005	0.124
**rno-miR-632-241110_mat**	32.333	7.642	0.515	0.005	0.133
**rno-miR-489-001353**	30.567	5.877	0.472	0.003	0.115
mmu-miR-682-4381081	35.118	9.530	25.931		0.133
mmu-miR-31#-002495	35.550	10.924	14.821		0.059
mmu-miR-125b-3p-4395489	32.111	6.588	12.800		0.044
mmu-miR-147-4395373	35.149	9.626	12.148		0.138
mmu-miR-10b#-002572	33.285	8.527	7.604		0.043
mmu-miR-200b-4395362	34.463	9.466	7.529		0.046
mmu-miR-467H-002809	34.143	9.455	6.890		0.042
mmu-miR-342-5p-4395657	35.210	9.621	5.309		0.106
mmu-miR-217-4395686	34.866	9.343	4.628		0.112
mmu-miR-7a-4378130	34.021	8.465	4.440	0.196	0.069
mmu-miR-540-5p-4395691	33.816	8.819	4.300		0.096
mmu-miR-449a-4373207	33.726	7.924	3.486	0.220	0.141
mmu-miR-669D-002808	34.944	10.318	3.289		0.041
rno-miR-379#-002081	33.507	8.873	2.936		0.041
mmu-miR-503#-002536	34.884	10.193	2.771	0.064	0.102
mmu-miR-380-5p-4395731	32.942	7.141	2.722	0.196	0.107
mmu-miR-187-4373307	33.347	7.673	2.522	0.582	0.125
mmu-miR-434-5p-4395711	34.294	8.485	2.483		0.109
hsa-miR-29a#-002447	32.166	7.476	2.460		0.048
mmu-miR-200c-4395411	32.594	6.923	2.341	0.092	0.061

Table Supplementary 5 Results of miRNA expression analysis using TaqMan Low Density Arrays

Target Name (ID LifeTech)	Cт Mean	ΔCт Mean	RQ	P-Value	Threshold
hsa-miR-299-5p-000600	31.462	6.771	2.328	0.136	0.114
mmu-miR-125a-3p-4395310	34.638	8.757	2.292	0.156	0.111
mmu-miR-181c-4373115	34.829	9.157	2.108	0.674	0.041
mmu-miR-542-5p-4395693	33.274	7.473	2.086	0.081	0.067
mmu-miR-1928-121164_mat	34.617	9.927	2.016	0.289	0.115
mmu-miR-331-5p-4395344	33.375	7.711	1.984		0.146
mmu-miR-98-4373009	32.945	7.143	1.848	0.240	0.066
mmu-miR-382-4373019	31.617	5.816	1.840	0.597	0.059
mmu-miR-685-4386748	32.566	6.902	1.803	0.122	0.065
mmu-miR-544-4395680	34.522	8.691	1.776		0.115
mmu-miR-34a-4395168	29.837	4.165	1.756	0.257	0.044
hsa-miR-27b#-002174	31.962	7.272	1.716	0.175	0.073
mmu-miR-345-5p-4395658	33.106	7.305	1.694		0.097
rno-miR-450a-4381124	31.653	5.851	1.679	0.046	0.177
mmu-miR-184-4373113	32.954	7.153	1.617		0.128
mmu-miR-376b#-002451	30.629	5.939	1.586	0.134	0.172
mmu-miR-217-001133	35.522	10.834	1.566		0.069
hsa-miR-340-000550	33.369	8.712	1.564	0.551	0.136
rno-miR-17-3p-4395779	34.111	8.428	1.547	0.925	0.149
rno-miR-351-4395764	32.020	6.219	1.509	0.197	0.084
mmu-miR-410-4378093	31.998	6.197	1.492	0.193	0.102
mmu-miR-23b-4373073	30.722	4.921	1.491	0.074	0.134
mmu-miR-221-4373077	32.599	6.743	1.469		0.044
hsa-miR-136#-002100	30.499	5.809	1.465	0.208	0.130
mmu-miR-155-4395701	30.452	4.650	1.460	0.150	0.288
hsa-miR-376a#-001287	34.614	9.756	1.458		0.124
hsa-miR-338-5P-002658	30.652	5.962	1.457	0.265	0.068
mmu-miR-212-002551	30.138	5.448	1.454	0.367	0.098
mmu-miR-337-000193	31.695	7.004	1.442	0.100	0.146
mmu-miR-345-001137	33.053	8.395	1.420	0.581	0.095
hsa-miR-26b#-002444	33.646	8.955	1.417	0.164	0.075
mmu-miR-411-4381013	28.173	2.371	1.415	0.176	0.166
mmu-miR-487b-001306	30.634	5.944	1.409	0.290	0.144
mmu-miR-675-3p-4386762	31.710	5.908	1.391	0.529	0.068
mmu-miR-21-4373090	29.643	3.842	1.377	0.274	0.096
mmu-miR-672-4395438	32.323	6.659	1.374	0.884	0.124
mmu-let-7g#-002492	34.619	10.000	1.362	0.614	0.119
mmu-miR-322#-002506	29.126	4.436	1.361	0.042	0.171

Table Supplementary 5 Results of miRNA expression analysis using TaqMan Low Density Arrays

Target Name (ID LifeTech)	Cт Mean	ΔCт Mean	RQ	P-Value	Threshold
mmu-miR-362-5p-002614	33.366	8.895	1.360	0.953	0.114
mmu-miR-199b-001131	34.154	9.496	1.349	0.627	0.056
mmu-miR-10b-4395329	31.828	6.027	1.347	0.258	0.190
mmu-miR-19b-4373098	26.358	0.557	1.333	0.090	0.148
mmu-miR-1939-121180_mat	33.137	8.447	1.324	0.185	0.084
mmu-miR-194-4373106	32.716	7.052	1.323	0.271	0.232
rno-miR-29c#-001818	33.415	8.725	1.313	0.861	0.078
mmu-miR-379-4373349	30.737	4.935	1.296	0.272	0.135
mmu-miR-92a-4373013	29.991	4.189	1.283	0.161	0.253
mmu-miR-487b-4378102	31.689	5.887	1.272	0.172	0.163
mmu-miR-19a-4373099	30.326	4.525	1.270	0.192	0.146
mmu-miR-1954-121137_mat	32.448	7.758	1.259	0.258	0.048
mmu-miR-191-4395410	22.609	-3.193	1.252	0.165	0.155
rno-miR-381-4381102	32.278	6.607	1.249	0.315	0.066
mmu-let-7c-1#-002479	33.463	9.210	1.242		0.083
mmu-miR-451-4373360	33.303	7.502	1.239	0.524	0.047
mmu-miR-30c-4373060	23.386	-2.416	1.234	0.048	0.187
mmu-miR-133a-4395357	17.328	-8.473	1.234	0.147	0.110
mmu-miR-18a-4395533	35.276	9.567	1.234		0.146
mmu-miR-323-3p-4395338	32.688	6.887	1.232	0.781	0.083
mmu-let-7g-4395393	28.179	2.377	1.230	0.058	0.189
mmu-miR-384-5p-4395732	31.856	6.055	1.229	0.345	0.256
rno-miR-1-4395765	20.039	-5.763	1.223	0.006	0.159
mmu-miR-328-4373049	30.624	4.823	1.208	0.280	0.112
mmu-miR-127-4373147	30.550	4.748	1.200	0.345	0.173
mmu-miR-223-4395406	26.782	0.980	1.185	0.114	0.181
mmu-miR-30a-4373061	26.347	0.546	1.184	0.009	0.188
hsa-miR-183#-002270	33.472	8.673	1.181		0.073
mmu-miR-186-4395396	27.499	1.697	1.180	0.167	0.272
mmu-miR-130a-4373145	32.508	6.706	1.173	0.357	0.115
hsa-miR-744#-002325	35.139	10.449	1.158		0.069
mmu-miR-17-4395419	27.641	1.840	1.153	0.210	0.240
mmu-miR-126-3p-4395339	21.444	-4.357	1.148	0.203	0.140
mmu-miR-30b-4373290	24.723	-1.078	1.142	0.027	0.146
mmu-miR-26a-4395166	24.391	-1.410	1.138	0.119	0.110
mmu-miR-20a-4373286	29.095	3.294	1.134	0.360	0.192
mmu-miR-133b-4395358	21.383	-4.419	1.133	0.186	0.076
mmu-let-7d-4395394	29.358	3.556	1.132	0.358	0.158

Table Supplementary 5 Results of miRNA expression analysis using TaqMan Low Density Arrays

Target Name (ID LifeTech)	Cт Mean	ΔCт Mean	RQ	P-Value	Threshold
mmu-miR-148a-4373130	32.391	6.590	1.131	0.405	0.084
mmu-miR-433-4373205	32.036	6.372	1.130	0.288	0.082
hsa-miR-106b#-002380	32.412	7.722	1.129	0.862	0.097
rno-miR-7a#-002062	28.260	3.570	1.128	0.164	0.144
rno-miR-196c-4395750	30.479	4.678	1.124	0.570	0.189
hsa-miR-151-5P-002642	30.628	5.938	1.118	0.361	0.095
mmu-miR-26b-4395167	27.075	1.273	1.117	0.418	0.151
mmu-miR-101b-002531	28.939	4.249	1.115	0.220	0.240
mmu-miR-1937b-241023_mat	19.200	-5.490	1.113	0.476	0.182
mmu-miR-218-4373081	31.195	5.394	1.113	0.502	0.145
mmu-miR-30d-4373059	27.330	1.528	1.111	0.109	0.126
mmu-miR-365-4373194	30.746	4.945	1.107	0.458	0.113
mmu-miR-30e-4395334	25.376	-0.426	1.100	0.202	0.241
mmu-miR-16-4373121	23.358	-2.443	1.098	0.312	0.195
mmu-miR-128a-4395327	31.325	5.524	1.085	0.739	0.121
mmu-miR-150-4373127	24.490	-1.312	1.083	0.444	0.191
mmu-let-7e-4395517	27.258	1.457	1.072	0.553	0.124
mmu-miR-139-5p-4395400	28.439	2.637	1.070	0.470	0.159
mmu-miR-467F-002886	26.340	1.650	1.067	0.559	0.053
mmu-miR-720-001629	23.603	-1.087	1.067	0.668	0.176
mmu-miR-101a-4395364	32.396	6.595	1.067	0.779	0.152
hsa-miR-30e-3p-000422	24.077	-0.613	1.066	0.500	0.171
mmu-miR-24-4373072	21.359	-4.442	1.065	0.434	0.113
hsa-miR-30a-3p-000416	23.548	-1.142	1.065	0.539	0.218
mmu-miR-106a-4395589	28.885	3.084	1.065	0.543	0.188
mmu-miR-342-3p-4395371	28.323	2.522	1.063	0.471	0.098
mmu-miR-126-5p-4373269	27.248	1.447	1.062	0.447	0.190
mmu-miR-1937c-241011_mat	21.840	-2.850	1.057	0.495	0.152
rno-miR-7#-001338	29.745	5.055	1.057	0.537	0.185
mmu-miR-574-3p-4395460	28.240	2.438	1.054	0.561	0.149
hsa-miR-411#-002238	33.355	8.665	1.054	0.753	0.102
mmu-miR-484-4381032	29.321	3.520	1.047	0.547	0.144
mmu-miR-1186-002825	35.173	10.524	1.043		0.060
mmu-miR-103-4373158	31.129	5.328	1.041	0.800	0.062
mmu-miR-1193-002794	32.049	7.392	1.035	0.944	0.068
mmu-miR-29c-4395171	31.131	5.330	1.024	0.699	0.226
mmu-miR-1-4395333	22.408	-3.393	1.023	0.912	0.069
mmu-miR-140-4373374	28.042	2.241	1.021	0.826	0.237

Table Supplementary 5 Results of miRNA expression analysis using TaqMan Low Density Arrays

Target Name (ID LifeTech)	Cт Mean	ΔCт Mean	RQ	P-Value	Threshold
mmu-miR-190-4373110	33.477	7.675	1.020	0.615	0.045
mmu-miR-143-4395360	28.090	2.288	1.019	0.795	0.206
mmu-miR-107-4373154	34.296	8.560	1.018		0.092
mmu-miR-383-4381093	35.911	9.987	1.018		0.106
mmu-miR-203-4373095	32.742	6.940	1.013	0.908	0.148
hsa-miR-223-000526	23.888	-0.803	1.009	0.902	0.201
rno-miR-664-001323	27.942	3.252	1.004	0.883	0.197
mmu-miR-188-5p-4395431	33.791	7.989	1.003	0.450	0.075
mmu-miR-27b-4373068	29.981	4.180	1.000	0.964	0.168
rno-miR-190b-4395749	33.842	8.040	0.991	0.594	0.209
mmu-miR-450a-5p-4395414	32.584	6.913	0.984	0.655	0.067
mmu-miR-381-4373020	33.985	8.606	0.984	0.811	0.052
mmu-miR-145-4395389	25.458	-0.343	0.982	0.898	0.113
mmu-let-7c-4373167	27.642	1.841	0.979	0.864	0.147
mmu-miR-100-4373160	28.951	3.149	0.974	0.969	0.098
mmu-miR-125b-5p-4373148	27.867	2.066	0.973	0.974	0.136
mmu-miR-99b-4373007	30.183	4.382	0.972	0.830	0.055
mmu-miR-28-4373067	31.340	5.539	0.972	0.935	0.149
rno-miR-339-3p-4395760	29.827	4.026	0.967	0.852	0.082
hsa-miR-206-000510	17.884	-6.806	0.965	0.698	0.207
mmu-miR-466k-240990_mat	32.718	8.028	0.965	0.971	0.069
mmu-miR-494-4395476	32.534	6.732	0.964	0.466	0.082
mmu-miR-1839-5p-121135_mat	33.235	8.544	0.960	0.746	0.110
rno-miR-350-001337	33.981	9.008	0.957		0.061
mmu-miR-541-002562	33.598	8.820	0.955	0.845	0.083
mmu-miR-322-001059	30.588	5.898	0.954	0.926	0.076
mmu-miR-195-4373105	26.140	0.338	0.953	0.468	0.194
mmu-miR-337-3p-4395662	33.904	8.102	0.953	0.806	0.117
mmu-miR-694-001681	27.641	2.951	0.947	0.820	0.049
mmu-miR-208b-4395401	32.996	7.195	0.942	0.861	0.186
hsa-miR-22-000398	26.359	1.669	0.942	0.983	0.057
rno-miR-504-007334	33.664	8.985	0.939		0.074
mmu-let-7f-4373164	31.013	5.211	0.934	0.692	0.045
mmu-miR-222-4395387	28.512	2.710	0.929	0.437	0.259
mmu-miR-486-4378096	24.911	-0.890	0.928	0.318	0.147
mmu-miR-10a-4373153	31.268	5.466	0.927	0.850	0.115
mmu-miR-351-4373345	32.447	6.645	0.926	0.952	0.045
mmu-miR-15b-4373122	31.970	6.169	0.917	0.934	0.137

Table Supplementary 5 Results of miRNA expression analysis using TaqMan Low Density Arrays

Target Name (ID LifeTech)	Cт Mean	ΔCт Mean	RQ	P-Value	Threshold
mmu-miR-27a-4373287	28.760	2.959	0.916	0.484	0.251
mmu-miR-34c#-002584	31.754	7.063	0.916	0.700	0.094
hsa-miR-423-3P-002626	29.796	5.106	0.914	0.734	0.127
mmu-miR-152-4395170	30.132	4.331	0.909	0.732	0.222
mmu-miR-2134-241120_mat	26.673	1.983	0.903	0.941	0.220
mmu-let-7i-4395332	28.702	2.901	0.902	0.275	0.178
hsa-miR-143-000466	31.768	7.077	0.902	0.915	0.127
mmu-miR-125a-5p-4395309	28.171	2.369	0.896	0.973	0.157
mmu-miR-148b-4373129	35.517	9.769	0.885		0.087
mmu-let-7a-4373169	29.992	4.190	0.884	0.822	0.076
mmu-miR-744-4395435	32.545	6.743	0.883	0.674	0.187
mmu-miR-872#-002542	29.561	4.871	0.881	0.307	0.102
hsa-miR-455-001280	33.301	8.611	0.880		0.084
mmu-miR-138-4395395	30.071	4.270	0.876	0.265	0.249
mmu-miR-99a-4373008	28.923	3.122	0.876	0.524	0.072
rno-miR-532-5p-4395752	30.795	4.994	0.876	0.657	0.174
mmu-miR-500-4395736	33.350	7.901	0.873		0.075
mmu-miR-181a-4373117	32.040	6.239	0.869	0.642	0.062
mmu-miR-532-3p-4395466	31.467	5.666	0.862	0.396	0.161
mmu-miR-196b-4395326	28.456	2.655	0.861	0.392	0.168
mmu-miR-15a-4373123	31.436	5.635	0.856	0.542	0.109
mmu-miR-499-4381047	30.896	5.094	0.854	0.593	0.224
mmu-miR-93-4373302	31.333	5.531	0.854	0.605	0.205
mmu-miR-146b-4373178	27.424	1.622	0.853	0.229	0.168
hsa-miR-200c-000505	33.184	8.494	0.851	0.444	0.115
rno-miR-352-001339	30.498	5.808	0.851	0.521	0.061
mmu-miR-592-002017	33.428	8.749	0.851	0.556	0.083
hsa-miR-196a-241070_mat	24.755	0.064	0.849	0.295	0.151
mmu-miR-24-2#-002494	27.432	2.742	0.843	0.309	0.129
mmu-miR-191#-002576	34.894	10.424	0.842	0.643	0.118
rno-miR-409-3P-002679	31.124	6.434	0.841	0.414	0.145
rno-miR-99a#-002073	32.963	8.273	0.833	0.981	0.059
mmu-miR-199a-3p-4395415	27.869	2.068	0.831	0.061	0.193
rno-miR-125b#-002074	30.901	6.210	0.826	0.055	0.137
hsa-miR-421-002700	31.761	7.071	0.825	0.544	0.201
mmu-miR-339-5p-4395368	31.886	6.085	0.824	0.583	0.064
mmu-miR-495-4381078	32.034	6.233	0.822	0.993	0.052
mmu-miR-320-4395388	27.293	1.492	0.820	0.301	0.193

Table Supplementary 5 Results of miRNA expression analysis using TaqMan Low Density Arrays

Target Name (ID LifeTech)	Cт Mean	ΔCт Mean	RQ	P-Value	Threshold
mmu-miR-1274a-121150_mat	23.175	-1.516	0.820	0.484	0.137
hsa-miR-22#-002301	30.789	6.098	0.819	0.326	0.067
hsa-miR-29b-2#-002166	35.103	10.131	0.814		0.191
mmu-miR-193b-4395597	27.476	1.675	0.809	0.079	0.183
mmu-miR-706-001641	31.401	6.711	0.809	0.347	0.050
hsa-miR-140-3p-002234	29.366	4.676	0.799	0.061	0.076
hsa-miR-378-000567	28.736	4.046	0.796	0.521	0.247
mmu-miR-374-5p-001319	26.901	2.211	0.793	0.084	0.109
hsa-miR-28-3p-002446	27.929	3.239	0.785	0.004	0.195
mmu-miR-10b-001181	32.210	7.519	0.780	0.480	0.101
mmu-miR-877#-002548	30.433	5.743	0.778	0.103	0.079
mmu-miR-497-4381046	31.003	5.202	0.778	0.136	0.178
mmu-miR-376a-4373347	34.858	9.057	0.777		0.086
mmu-miR-340-3p-4395370	34.619	8.775	0.776	0.650	0.147
mmu-miR-543-4395487	34.138	9.140	0.772		0.096
mmu-miR-350-4395660	34.552	8.895	0.767		0.070
hsa-miR-93#-002139	30.584	5.893	0.765	0.222	0.186
mmu-miR-2135-241140_mat	28.826	4.135	0.765	0.249	0.103
hsa-miR-149-002255	25.894	1.204	0.763	0.010	0.140
mmu-miR-872-4395375	33.760	7.958	0.760	0.150	0.272
mmu-miR-28#-002545	28.802	4.112	0.754	0.119	0.071
mmu-miR-25-4373071	33.237	7.435	0.753		0.207
mmu-miR-409-3p-4395443	32.122	6.321	0.751	0.369	0.238
mmu-miR-146a-4373132	25.815	0.014	0.748	0.026	0.194
mmu-miR-431-4395173	31.518	5.717	0.748	0.296	0.087
mmu-miR-214-4395417	29.331	3.530	0.747	0.083	0.122
mmu-miR-423-5p-4395451	33.770	8.121	0.744		0.059
mmu-miR-503-4395586	32.747	6.946	0.738	0.504	0.090
mmu-miR-466g-241015_mat	33.924	9.234	0.738	0.782	0.050
mmu-miR-652-4395463	32.696	6.894	0.737	0.479	0.126
mmu-miR-543-001298	34.757	9.979	0.732		0.141
mmu-miR-1961-197391_mat	31.963	7.273	0.730	0.264	0.050
mmu-miR-106b-4373155	32.079	6.278	0.724	0.217	0.202
mmu-miR-193#-002577	32.892	8.202	0.717	0.204	0.157
hsa-miR-213-000516	34.395	9.737	0.708	0.614	0.045
mmu-miR-2138-241080_mat	27.902	3.212	0.706	0.929	0.047
mmu-miR-134-4373299	31.548	5.747	0.700	0.217	0.084
mmu-let-7b-4373168	29.392	3.590	0.692	0.067	0.138

Table Supplementary 5 Results of miRNA expression analysis using TaqMan Low Density Arrays

Target Name (ID LifeTech)	Cт Mean	ΔCт Mean	RQ	P-Value	Threshold
mmu-miR-324-5p-4373052	33.674	8.010	0.690	0.796	0.065
mmu-miR-369-5p-4373195	34.560	8.920	0.689	0.313	0.044
mmu-miR-29b#-002497	34.030	9.301	0.689		0.152
mmu-miR-1896-121128_mat	31.420	6.729	0.680	0.110	0.057
mmu-miR-2146-241082_mat	23.582	-1.108	0.680	0.027	0.087
mmu-miR-34b-3p-4395748	33.293	7.825	0.679	0.827	0.072
mmu-let-7a#-002478	32.490	7.800	0.678	0.081	0.101
hsa-miR-214#-002293	31.932	7.241	0.674	0.024	0.151
mmu-miR-202-3p-4373311	32.281	6.479	0.662	0.090	0.052
mmu-miR-142-3p-4373136	32.328	6.526	0.660	0.433	0.158
mmu-miR-182-4395729	31.939	6.138	0.647	0.610	0.117
mmu-miR-1839-3p-121203_mat	32.599	7.908	0.645	0.224	0.056
mmu-miR-326-001061	33.519	8.862	0.633	0.278	0.046
mmu-miR-467b-001684	33.037	8.347	0.630	0.520	0.040
mmu-miR-1897-5p-121199_mat	31.190	6.500	0.627	0.236	0.066
hsa-miR-33a#-002136	33.753	9.062	0.624	0.273	0.048
mmu-miR-1894-3p-241002_mat	34.044	9.354	0.623	0.156	0.114
mmu-miR-335-5p-4373045	32.789	6.988	0.613	0.330	0.113
hsa-miR-324-3p-000579	30.077	5.387	0.611	0.204	0.082
mmu-miR-361-4373035	34.096	8.252	0.611	0.603	0.080
mmu-miR-708-4395452	33.560	7.896	0.599		0.238
mmu-miR-335-3p-4395296	34.718	9.027	0.597	0.546	0.097
mmu-miR-130b-4373144	30.881	5.080	0.594	0.097	0.057
mmu-miR-192-4373108	33.184	7.383	0.583	0.758	0.196
mmu-miR-671-3p-4395433	35.120	9.411	0.581		0.137
mmu-miR-485-3p-001943	33.299	8.665	0.576	0.222	0.056
mmu-miR-674#-001956	31.662	6.971	0.570	0.313	0.077
mmu-miR-301a-4373064	33.191	7.520	0.534	0.307	0.107
mmu-miR-301b-4395730	33.778	7.977	0.526	0.212	0.117
mmu-miR-205-4373093	34.758	9.049	0.506	0.103	0.080
hsa-miR-189-000488	34.515	9.916	0.505	0.786	0.040
rno-miR-224-4373187	35.145	9.884	0.498		0.073
mmu-miR-340-5p-4395369	33.847	8.046	0.496	0.054	0.121
hsa-miR-425-001104	33.898	9.208	0.477	0.311	0.061
mmu-miR-31-4373331	36.162	9.987	0.474	0.643	0.161
mmu-miR-193-4395361	34.487	9.038	0.466		0.111
hsa-miR-99b#-002196	32.909	8.219	0.463	0.266	0.057
rno-miR-345-3p-4395762	33.537	7.735	0.443	0.160	0.043

Table Supplementary 5 Results of miRNA expression analysis using TaqMan Low Density Arrays

Target Name (ID LifeTech)	Cт Mean	ΔCт Mean	RQ	P-Value	Threshold
mmu-miR-185-4395382	33.476	7.675	0.430	0.061	0.066
rno-miR-505-4381097	34.223	8.930	0.416		0.060
mmu-miR-690-001677	28.987	4.297	0.375	0.120	0.115
mmu-miR-384-3p-4395733	37.260	11.704	0.374		0.070
mmu-miR-9-4373285	34.199	8.732	0.337		0.079
hsa-let-7i#-002172	35.529	10.903	0.328		0.114
hsa-miR-412-001023	35.118	10.146	0.311		0.063
mmu-miR-409-5p-4395442	36.468	10.719	0.286		0.051
mmu-miR-338-3p-4395363	36.708	10.972	0.265		0.140
mmu-miR-704-001639	37.417	12.894	0.262		0.046
hsa-miR-10a#-002288	34.918	10.665	0.239		0.055
hsa-miR-338-000548	37.182	12.556	0.173		0.064
mmu-miR-296-5p-4373066	37.185	11.477	0.166		0.041
mmu-miR-183-4395380	36.849	11.207	0.146		0.051
mmu-miR-196a#-002477	36.743	11.984	0.116	0.502	0.040
mmu-miR-15a#-002488	38.270	13.831	0.086	0.085	0.046
mmu-miR-1949-121182_mat	39.711	15.458	0.013		0.070
ath-miR159a-000338					0.200
ath-miR159a-4373390					0.200
hsa-let-7b#-002404					0.040
hsa-let-7e#-002407					0.040
hsa-let-7f-1#-002417					0.040
hsa-miR-1197-002810					0.200
hsa-miR-124#-002197					0.200
hsa-miR-127-5p-002229	35.250	10.562			0.130
hsa-miR-136-000592					0.040
hsa-miR-144-002676					0.040
hsa-miR-148a#-002134					0.200
hsa-miR-154#-000478					0.156
hsa-miR-15b#-002173					0.040
hsa-miR-190b-002263					0.040
hsa-miR-200a#-001011					0.200
hsa-miR-200b#-002274					0.200
hsa-miR-200b-001800					0.040
hsa-miR-200c#-002286					0.200
hsa-miR-218-2#-002294					0.040
hsa-miR-23a#-002439					0.200
hsa-miR-30c-1#-002108					0.040

Table Supplementary 5 Results of miRNA expression analysis using TaqMan Low Density Arrays

Target Name (ID LifeTech)	Cт Mean	ΔCт Mean	RQ	P-Value	Threshold
hsa-miR-30c-2#-002110					0.040
hsa-miR-363#-001283					0.200
hsa-miR-431#-002312					0.200
hsa-miR-485-5p-001036					0.200
hsa-miR-493-3p-001282					0.040
hsa-miR-590-3P-002677					0.200
hsa-miR-653-002292					0.200
hsa-miR-671-5p-197646_mat					0.040
hsa-miR-708#-002342					0.200
hsa-miR-875-5p-002203					0.040
hsa-miR-9#-002231					0.040
hsa-miR-935-002178					0.200
Mamm U6-001973	17.528				0.060
MammU6-4395470	18.120				0.069
mmu-let-7d#-001178					0.040
mmu-miR-101a#-002507					0.040
mmu-miR-105-4395595					0.200
mmu-miR-1188-002866					0.040
mmu-miR-1191-002892					0.040
mmu-miR-1192-002806					0.200
mmu-miR-1194-002793					0.200
mmu-miR-1195-002839					0.200
mmu-miR-1198-002780					0.200
mmu-miR-1199-240984_mat					0.200
mmu-miR-1224-240985_mat					0.040
mmu-miR-122-4395356	35.046	9.522			0.064
mmu-miR-124-4373295					0.040
mmu-miR-125b#-002508					0.040
mmu-miR-129-3p-4373297	34.933	9.409			0.152
mmu-miR-129-5p-4373171					0.200
mmu-miR-1-2-AS-002882					0.040
mmu-miR-1306-121155_mat					0.040
mmu-miR-130b#-002460					0.200
mmu-miR-133a#-001637					0.040
mmu-miR-135a-4373140					0.040
mmu-miR-135b-4395372					0.172
mmu-miR-136#-002512					0.040
mmu-miR-137-4373301					0.200

Table Supplementary 5 Results of miRNA expression analysis using TaqMan Low Density Arrays

Target Name (ID LifeTech)	Cт Mean	ΔCт Mean	RQ	P-Value	Threshold
mmu-miR-138#-002554					0.040
mmu-miR-139-3p-4395676					0.040
mmu-miR-141#-002513					0.040
mmu-miR-141-4373137					0.040
mmu-miR-142-5p-4395359					0.040
mmu-miR-145#-002514					0.200
mmu-miR-146b#-002453					0.073
mmu-miR-150#-002570					0.200
mmu-miR-151-3p-4373304					0.040
mmu-miR-153-4373305					0.200
mmu-miR-154-4373270					0.200
mmu-miR-16#-002489					0.040
mmu-miR-17#-002543					0.040
mmu-miR-181A-2#-002687					0.200
mmu-miR-186#-002574					0.040
mmu-miR-188-3p-4395217					0.200
mmu-miR-1893-121170_mat					0.200
mmu-miR-1894-5p-121144_mat					0.040
mmu-miR-1897-3p-121126_mat	37.439	12.826			0.045
mmu-miR-1898-121195_mat					0.040
mmu-miR-1899-121198_mat					0.200
mmu-miR-18a#-002490					0.064
mmu-miR-18b-4395596	34.661	9.663			0.181
mmu-miR-1900-121143_mat					0.200
mmu-miR-1901-121183_mat					0.040
mmu-miR-1902-121197_mat					0.200
mmu-miR-1903-121153_mat					0.040
mmu-miR-1904-121162_mat					0.040
mmu-miR-1905-121196_mat					0.040
mmu-miR-1906-121169_mat					0.040
mmu-miR-1927-121193_mat					0.200
mmu-miR-1930-121201_mat					0.040
mmu-miR-1931-121168_mat					0.200
mmu-miR-1932-121172_mat					0.200
mmu-miR-1933-3p-121145_mat					0.200
mmu-miR-1933-5p-121133_mat					0.200
mmu-miR-1934-121185_mat					0.200
mmu-miR-1935-121192_mat					0.200

Table Supplementary 5 Results of miRNA expression analysis using TaqMan Low Density Arrays

Target Name (ID LifeTech)	Cт Mean	ΔCт Mean	RQ	P-Value	Threshold
mmu-miR-1936-121158_mat					0.040
mmu-miR-1938-121194_mat					0.200
mmu-miR-1940-121187_mat					0.040
mmu-miR-1941-3p-121130_mat					0.200
mmu-miR-1941-5p-121140_mat					0.200
mmu-miR-1942-121136_mat					0.040
mmu-miR-1943-121174_mat					0.040
mmu-miR-1944-121189_mat					0.200
mmu-miR-1945-121166_mat					0.200
mmu-miR-1946a-121178_mat					0.040
mmu-miR-1947-121156_mat					0.200
mmu-miR-1948-121171_mat					0.040
mmu-miR-1950-121146_mat					0.200
mmu-miR-1951-121165_mat					0.040
mmu-miR-1952-121167_mat					0.200
mmu-miR-1953-121159_mat					0.040
mmu-miR-1956-121129_mat					0.200
mmu-miR-1957-121163_mat					0.040
mmu-miR-1958-121181_mat					0.200
mmu-miR-1959-121132_mat					0.040
mmu-miR-1960-121148_mat					0.040
mmu-miR-1962-121173_mat					0.040
mmu-miR-1963-121191_mat					0.200
mmu-miR-1964-121138_mat					0.053
mmu-miR-1965-121186_mat					0.200
mmu-miR-1966-121134_mat					0.200
mmu-miR-1967-121151_mat					0.200
mmu-miR-1968-121179_mat					0.200
mmu-miR-1969-121131_mat					0.040
mmu-miR-1970-121202_mat					0.200
mmu-miR-1971-121161_mat					0.040
mmu-miR-197-4373102					0.040
mmu-miR-1981-121200_mat					0.200
mmu-miR-1982.1-121157_mat					0.040
mmu-miR-1982.2-121154_mat					0.200
mmu-miR-199a-5p-4373272					0.040
mmu-miR-19a#-002544					0.040
mmu-miR-200a-4378069					0.040

Table Supplementary 5 Results of miRNA expression analysis using TaqMan Low Density Arrays

Target Name (ID LifeTech)	Cт Mean	ΔCт Mean	RQ	P-Value	Threshold
mmu-miR-201-4395708					0.200
mmu-miR-202-5p-4395709					0.200
mmu-miR-203#-002580					0.200
mmu-miR-207-4373314					0.040
mmu-miR-208-4373091					0.200
mmu-miR-20a#-002491					0.200
mmu-miR-20b#-002524					0.200
mmu-miR-20b-4373263					0.040
mmu-miR-21#-002493					0.130
mmu-miR-211-4373315					0.040
mmu-miR-2136-241133_mat	34.845	9.872			0.060
mmu-miR-2139-241130_mat					0.200
mmu-miR-215-4373316	35.057	8.607			0.208
mmu-miR-216a-4395331					0.200
mmu-miR-216b-4395437					0.200
mmu-miR-218-1#-002552					0.200
mmu-miR-2182-241119_mat					0.040
mmu-miR-2183-241095_mat					0.040
mmu-miR-219-4373080					0.200
mmu-miR-220-4395598					0.200
mmu-miR-224-4395683					0.040
mmu-miR-23a-4373074					0.040
mmu-miR-290-000187					0.200
mmu-miR-290-3p-4395721					0.200
mmu-miR-290-5p-002590					0.200
mmu-miR-291-3p-001135					0.200
mmu-miR-291-5p-001202					0.200
mmu-miR-291a-3p-4395722					0.200
mmu-miR-291b-3p-002538					0.200
mmu-miR-291b-5p-4395667					0.200
mmu-miR-292-3p-001054					0.040
mmu-miR-292-3p-4395723					0.040
mmu-miR-292-5p-001055					0.200
mmu-miR-293#-002594					0.040
mmu-miR-293-4386754					0.200
mmu-miR-294#-002595					0.200
mmu-miR-294-4373326					0.200
mmu-miR-295#-002596					0.200

Table Supplementary 5 Results of miRNA expression analysis using TaqMan Low Density Arrays

Target Name (ID LifeTech)	Cт Mean	ΔCт Mean	RQ	P-Value	Threshold
mmu-miR-295-4373327					0.200
mmu-miR-296-3p-4395212					0.200
mmu-miR-297a#-002454					0.040
mmu-miR-297b-5p-4381049					0.200
mmu-miR-297c-4395610					0.200
mmu-miR-298-4395728					0.078
mmu-miR-299-002612					0.040
mmu-miR-300#-002613	35.470	10.782			0.040
mmu-miR-300-000191					0.200
mmu-miR-302a#-002615					0.200
mmu-miR-302a-4378070					0.040
mmu-miR-302b#-001307					0.200
mmu-miR-302b-4378071					0.200
mmu-miR-302c#-002557					0.200
mmu-miR-302c-4395688					0.040
mmu-miR-302d-4373063					0.200
mmu-miR-30b#-002498					0.040
mmu-miR-32-4395220					0.161
mmu-miR-324-3p-4395639					0.040
mmu-miR-325-001060					0.067
mmu-miR-325-4395640					0.120
mmu-miR-327-002481					0.040
mmu-miR-329-4373336					0.152
mmu-miR-330-001062					0.200
mmu-miR-330-4395341					0.200
mmu-miR-339-3p-4395663					0.040
mmu-miR-343-002483					0.200
mmu-miR-344-4373340					0.200
mmu-miR-345-3p-4395659					0.200
mmu-miR-346-4373342					0.200
mmu-miR-34b-001065					0.040
mmu-miR-34b-5p-002617					0.200
mmu-miR-34c-4373036					0.200
mmu-miR-362-3p-4395746					0.040
mmu-miR-363-4378090					0.200
mmu-miR-367-4373034					0.200
mmu-miR-369-3p-4373032					0.040
mmu-miR-370-001068					0.200

Table Supplementary 5 Results of miRNA expression analysis using TaqMan Low Density Arrays

Target Name (ID LifeTech)	Cт Mean	ΔCт Mean	RQ	P-Value	Threshold
mmu-miR-370-4395386					0.040
mmu-miR-374#-002043					0.040
mmu-miR-375-4373027					0.200
mmu-miR-376a#-002482					0.040
mmu-miR-376b-4395582					0.040
mmu-miR-376c#-002523					0.200
mmu-miR-377-4373025					0.200
mmu-miR-380-3p-4373350					0.200
mmu-miR-412-002575	35.472	10.779			0.074
mmu-miR-425-4380926					0.040
mmu-miR-429-4373355	34.952	9.328			0.065
mmu-miR-432-241135_mat					0.040
mmu-miR-433-5p-001078					0.200
mmu-miR-448-4373206					0.200
mmu-miR-449b-001667					0.200
mmu-miR-449b-4395669					0.200
mmu-miR-450a-3p-002525					0.200
mmu-miR-450B-3P-002632					0.040
mmu-miR-450b-5p-4386779					0.040
mmu-miR-452-4373281					0.040
mmu-miR-453-4395614					0.200
mmu-miR-455-4395585					0.200
mmu-miR-463#-002582					0.200
mmu-miR-463-002662					0.040
mmu-miR-464-4373362					0.200
mmu-miR-465a-3p-4395574					0.200
mmu-miR-465a-5p-4373363					0.040
mmu-miR-465b-5p-4395615					0.200
mmu-miR-465C-5P-002654					0.040
mmu-miR-466a-3p-002586					0.200
mmu-miR-466b-3-3p-002500					0.200
mmu-miR-466d-5p-002534					0.200
mmu-miR-466E-5P-002718					0.200
mmu-miR-466h-4395646					0.040
mmu-miR-466J-002817					0.040
mmu-miR-467a-001826					0.040
mmu-miR-467a-4395717					0.200
mmu-miR-467b-4381084					0.040

Table Supplementary 5 Results of miRNA expression analysis using TaqMan Low Density Arrays

Target Name (ID LifeTech)	Cт Mean	ΔCт Mean	RQ	P-Value	Threshold
mmu-miR-467c-4395647					0.200
mmu-miR-467d-4395648					0.200
mmu-miR-467e#-002569					0.200
mmu-miR-467e-4395698					0.040
mmu-miR-468-4373366					0.200
mmu-miR-469-4373367					0.200
mmu-miR-470#-002589					0.040
mmu-miR-470-4395718					0.200
mmu-miR-471-002605					0.200
mmu-miR-483#-002560					0.040
mmu-miR-483-001291					0.200
mmu-miR-488#-002014					0.200
mmu-miR-488-4381074					0.040
mmu-miR-489-4378114					0.040
mmu-miR-490-4373215					0.040
mmu-miR-491-4381053					0.040
mmu-miR-493-4395649					0.200
mmu-miR-494-001293	34.975	10.173			0.176
mmu-miR-496-4386771					0.200
mmu-miR-501-001356	33.702	9.014			0.060
mmu-miR-501-3p-4381069					0.040
mmu-miR-504-4395195					0.200
mmu-miR-505-4381071					0.200
mmu-miR-509-3p-4395651					0.040
mmu-miR-509-5p-4395650					0.200
mmu-miR-511-4395679					0.040
mmu-miR-532-5p-4380928					0.040
mmu-miR-540-3p-4378119					0.040
mmu-miR-542-3p-4378101					0.040
mmu-miR-546-4381044					0.200
mmu-miR-547-4395694					0.040
mmu-miR-551b-4380945					0.200
mmu-miR-582-3p-4395697					0.200
mmu-miR-582-5p-4395696	35.274	9.751			0.062
mmu-miR-590-5p-4395176					0.040
mmu-miR-598-4395606					0.200
mmu-miR-599-241117_mat					0.200
mmu-miR-615-3p-4386777					0.040

Table Supplementary 5 Results of miRNA expression analysis using TaqMan Low Density Arrays

Target Name (ID LifeTech)	Cт Mean	ΔCт Mean	RQ	P-Value	Threshold
mmu-miR-615-5p-4395464					0.040
mmu-miR-654-3p-4395350					0.200
mmu-miR-654-5p-4395652					0.200
mmu-miR-665-4395737					0.040
mmu-miR-666-3p-002448					0.200
mmu-miR-666-5p-4386770					0.040
mmu-miR-667-4386769					0.040
mmu-miR-668-4386767					0.200
mmu-miR-669a-4381091					0.200
mmu-miR-669C-002646					0.040
mmu-miR-669E-002774					0.200
mmu-miR-669G-002813					0.200
mmu-miR-669H-5P-002906					0.200
mmu-miR-669l-121149_mat					0.040
mmu-miR-669m-121190_mat					0.200
mmu-miR-669n-197143_mat					0.040
mmu-miR-669o-121176_mat					0.200
mmu-miR-670-4395561					0.200
mmu-miR-673-001954					0.040
mmu-miR-673-3p-002449					0.040
mmu-miR-674-4395193					0.040
mmu-miR-675-5p-4386761					0.200
mmu-miR-676#-001958					0.040
mmu-miR-676-4386776					0.200
mmu-miR-677-4381075					0.200
mmu-miR-679-4381077					0.200
mmu-miR-680-4381079					0.200
mmu-miR-683-4381082					0.200
mmu-miR-684-4381083	35.246	10.249			0.141
mmu-miR-686-4381085					0.200
mmu-miR-687-4386750					0.040
mmu-miR-688-001675					0.200
mmu-miR-691-001678					0.200
mmu-miR-692-001679					0.200
mmu-miR-693-001680					0.200
mmu-miR-693-3p-002036					0.200
mmu-miR-695-001627					0.200
mmu-miR-696-001628					0.040

Table Supplementary 5 Results of miRNA expression analysis using TaqMan Low Density Arrays

Target Name (ID LifeTech)	Cт Mean	ΔCт Mean	RQ	P-Value	Threshold
mmu-miR-697-001631					0.040
mmu-miR-698-001632					0.040
mmu-miR-700-001634					0.200
mmu-miR-701-001635					0.040
mmu-miR-702-001636					0.200
mmu-miR-707-001642					0.200
mmu-miR-710-001645					0.200
mmu-miR-711-001646					0.200
mmu-miR-712#-001961					0.200
mmu-miR-712-002636					0.200
mmu-miR-713-001648					0.200
mmu-miR-715-001649					0.200
mmu-miR-717-001652					0.200
mmu-miR-718-001656					0.200
mmu-miR-719-001673					0.200
mmu-miR-721-001657					0.040
mmu-miR-741-4395587					0.040
mmu-miR-742#-002458					0.200
mmu-miR-742-4395573					0.200
mmu-miR-743a-4395599					0.040
mmu-miR-743b-3p-4395601					0.200
mmu-miR-743b-5p-4395600					0.200
mmu-miR-758-002025					0.200
mmu-miR-759-002034					0.200
mmu-miR-761-002030					0.040
mmu-miR-762-002028					0.200
mmu-miR-763-002033					0.040
mmu-miR-764-3p-002032					0.200
mmu-miR-764-5p-002031					0.040
mmu-miR-767-241081_mat					0.200
mmu-miR-770-3p-4395564					0.040
mmu-miR-770-5p-002608					0.200
mmu-miR-7b-4395685					0.040
mmu-miR-802-4395566					0.200
mmu-miR-804-002044					0.040
mmu-miR-805-002045					0.040
mmu-miR-871-4395465					0.200
mmu-miR-873-4395467					0.040

Table Supplementary 5 Results of miRNA expression analysis using TaqMan Low Density Arrays

Target Name (ID LifeTech)	Cт Mean	ΔCт Mean	RQ	P-Value	Threshold
mmu-miR-874-4395379					0.200
mmu-miR-875-3p-4395677					0.200
mmu-miR-876-3p-4395594					0.200
mmu-miR-876-5p-4395593					0.200
mmu-miR-878-3p-002541					0.040
mmu-miR-878-5p-4395670					0.200
mmu-miR-879#-002473					0.200
mmu-miR-879-4395602					0.200
mmu-miR-880-002665					0.040
mmu-miR-881#-002475					0.040
mmu-miR-881-4395739					0.200
mmu-miR-882-002610					0.200
mmu-miR-883a-3p-4395591					0.200
mmu-miR-883a-5p-4395741					0.200
mmu-miR-883b-3p-4395695					0.200
mmu-miR-883B-5P-002669					0.200
mmu-miR-92a#-002496					0.200
mmu-miR-96-4373372					0.200
rno-miR-1#-001351					0.040
rno-miR-105-241087_mat					0.200
rno-miR-135a#-002075					0.040
rno-miR-148b-5p-002058	35.365	10.677			0.145
rno-miR-204#-002076					0.040
rno-miR-207-4381096					0.040
rno-miR-20a#-001336					0.144
rno-miR-20b-001326					0.040
rno-miR-20b-3p-4395753					0.200
rno-miR-219-1-3p-4395778					0.200
rno-miR-219-2-3p-4395501					0.200
rno-miR-220-241101_mat					0.040
rno-miR-24-1#-002079					0.040
rno-miR-25#-002080					0.040
rno-miR-293-241102_mat					0.200
rno-miR-294-241093_mat					0.200
rno-miR-29b-1#-002082					0.200
rno-miR-327-4381108					0.040
rno-miR-333-4381109					0.040
rno-miR-336-4381111					0.040

Table Supplementary 5 Results of miRNA expression analysis using TaqMan Low Density Arrays

Target Name (ID LifeTech)	Cт Mean	ΔCт Mean	RQ	P-Value	Threshold
rno-miR-343-4381123					0.200
rno-miR-344-3p-4381112					0.200
rno-miR-344-5p-4395761					0.200
rno-miR-346-4381113					0.200
rno-miR-347-4381114					0.040
rno-miR-349-4381115					0.040
rno-miR-377-4381100					0.200
rno-miR-382#-001354					0.040
rno-miR-409-5p-4381098					0.200
rno-miR-421-4381122					0.200
rno-miR-465-241132_mat					0.040
rno-miR-466b-4395767					0.040
rno-miR-466c-4395768					0.040
rno-miR-513-241072_mat					0.200
rno-miR-543-4395766					0.200
rno-miR-547-241078_mat					0.040
rno-miR-551B-002760					0.200
rno-miR-598-5p-4395754					0.200
rno-miR-653-241125_mat	36.874	12.073			0.106
rno-miR-666-241074_mat					0.200
rno-miR-673-4395755					0.040
rno-miR-711-241136_mat					0.200
rno-miR-742-4395756					0.200
rno-miR-743a-002056					0.040
rno-miR-743b-4395769					0.040
rno-miR-758-4395180					0.118
rno-miR-760-5p-4395758					0.200
rno-miR-871-4395770					0.040
rno-miR-876-241138_mat					0.200
rno-miR-878-4395771					0.200
rno-miR-880-002666					0.200
rno-miR-881-4395773					0.200
snoRNA135-001230	29.499				0.084
snoRNA135-4380912	29.958				0.093
snoRNA202-001232					0.040
snoRNA202-4380914					0.040
U87-001712	24.482				0.088
U87-4386735	25.949				0.118

Table Supplementary 5 Results of miRNA expression analysis using TaqMan Low Density Arrays

Target Name (ID LifeTech)	Cт Mean	ΔCт Mean	RQ	P-Value	Threshold
Y1-001727	27.253				0.052
Y1-4386739	29.178				0.057

Table Suplementary S6 Target genes deregulated by differentially expressed miRNAs

GOTERM	GOID	GO Database	P-value	%	Genes (n)	miRNAs Associated (n)	Target Genes Deregulated	miRNAS
Extracellular matrix organization	6016202	REACTOME	9.2 E-5	8.08	21	11	[Adamts3, Col1a1, Col1a2, Col22a1, Col3a1, Col5a1, Col6a2, Itga8, Lum, Mmp15, Mmp16, Pdgfa, Serpine1, Pgf, Tln2, Abi3bp, Ccdc80, Sema5a, Smoc2, Plxna2, Sema6a]	[miR-132-3p, miR-204-5p, miR-214-3p, miR-29a-3p, miR-29b-3p, miR-30d-3p, miR-322-3p, miR-376c-3p, miR-450a-5p, miR-539-5p, miR-632]
Proteasome/Targeted protein degradation	3050	KEGG	3.0 E-5	17.24	5	4	[Psma3, Psmc6, Psmd11, Psmd2, Psmd3]	[miR-146b-5p, miR-214-3p, miR-30d-3p, miR-632]
Citric acid cycle and respiratory electron transport	5416705	REACTOME	5.6 E-4	3.10	4	5	[Atp5g1, Ndufa2, Ndufs1,Sdhd]	[miR-29a-3p, miR-29b-3p, miR-322-3p, miR-434-3p, miR-539-5p]
JNK cascade	7254	GO Biological Process	3.4 E-4	3.03	8	9	[Cdc42se1, Dusp10, Mecom, Sfrp4, Dusp22, Mapk10, Myd88, Elk1]	[miR-136-5p, miR-204-5p, miR-214-3p, miR-322-3p, miR-337-5p, miR-450a-5p, miR-489-3p, miR-539-5p, miR-632]
Cellular response to TGF beta	71560	GO Biological Process	7.8 E-3	5.38	12	9	[Col1a1, Col1a2, Col3a1, Dusp22, Gcnt2, Itga8, Klf10, Mecom, Pdgfa, Serpine1, Ube2d1, Cited2]	[miR-214-3p, miR-29a-3p, miR-29b-3p, miR-30d-3p, miR-322-3p, miR-376c-3p, miR-489-3p, miR-539-5p, miR-632]

Table Supplementary 7 Number of targets mRNAs per differentially expressed microRNAs in cardiac cachexia.

	mRNA targets (n)		
**miRNA**	**Predicted**	**Validated**	**Total**
miR-204-5p	24	0	24
miR-29b-3p	18	6	24
miR-136-5p	8	14	22
miR-322-3p	21	0	21
miR-539-5p	21	0	21
miR-29a-3p	15	5	20
miR-30d-3p	16	0	16
miR-132-3p	15	0	15
miR-214-3p	13	0	13
miR-632	10	0	10
miR-331-3p	7	0	7
miR-146b-5p	6	0	6
miR-434-3p	6	0	6
miR-376c-3p	5	0	5
miR-489-3p	5	0	5
miR-210-3p	2	0	2
miR-27a-5p	2	0	2
miR-337-5p	1	0	1

n: number of targets mRNAs

Table Supplementary 8 mRNAs co-deregulated by 3 or more miRNAs in cardiac cachexia.

mRNA	miRNA (n)	List of miRNAs
Fbxw7	5	miR-136-5p*, miR-204-5p, miR-29a-3p,miR-29b-3p, miR-322-3p
Dnmt3a	3	miR-29b-3p*, miR-29a-3p*, miR-204-5p
Ppic	3	miR-29b-3p,miR-29a-3p, miR-204-5p

* validated interaction, n: n: number of miRNAs

Table Supplementary 9 RT-qPCR primer sequences

mRNA	RefSeq	Sequence (5′ – 3′)
Trim63	NM_080903.1	F: GTGAAGTTGCCCCCTTACAA<br>R: TGGAGATGCAATTGCTCAGT
Fbxo32	NM_133521.1	F: GACCTGCATGTGCTCAGTGAAG<br>R: GGATCTGCCGCTCTGAGAAGT
MyH7b	NM_001107794.2	F: AAGCAGAGAGAGGAGCAAGCAGAA<br>R: ACTTTCACTCGAGGGTGAAGCAGT
TnnI1	NM_017184.1	F: AGCGCCCTTCAGGACTTATG<br>R: TGGAGGCATTTGGCTTCGAT
TnnI2	NM_017185.1	F: GCACCTGAAGAGTGTGATGC<br>R: GTTCTGCTTCTCGGATTCGC
TnnT3	NM_031532.1	F: GCGTCAGAACAAGGACCTCA<br>R: GCACGGCGCTTTTCAATTCT
Ctss	NM_017320.1	F: GGGCAGCTGAAGCTGAAAAC<br>R: TTCGGTTGAGCAATCCACCA
Cpn2	NM_017116.2	F: GGCCGTTACGTACCAGAAGT<br>R: TGAGCTTTCAACCTCCTCGG
Runx1	NM_017325.1	F: CCCCGAAGACATCGGCAGAA<br>R: CACTGAGCCGCTCGGAAAAG
TNF-α	NM_012675.3	F: TGATCGGTCCCAACAAGGA<br>R: GGGCCATGGAACTGATGAGA
Col1a1	NM_053304.1	F: ACCTGTGTGTTCCCCACTCA<br>R: CTTCTCCTTGGGGTTTGGGC
Col3a1	NM_032085.1	F: TGGGCCTCAAGGTGTAAAGG<br>R: GCCCTGGATTACCATTGTTGC
B2m	NM_012512.2	F: GGAGAATGGGAAGCCGAACA<br>R: GGATTTCAATGTGAGGCGGG
Ppia	NM_017101.1	F: TGGCAAATGCTGGACCAAAC<br>R: TGCCTTCTTTCACCTTCCCAA
Ppib	NM_022536.1	F: TCTCGGAGCGCAATATGAAGG<br>R: AGCAAAAGGAAGACGACGGA
Col1a1<br>Mus musculus	NM_007742.4	F: CCTGGACGCCATCAAGGTCTACTGC<br>R: ACTCGAACGGGAATCCATCGGTCAT
Col3a1<br>Mus musculus	NM_009930.2	F: TCCTGGTGGTCCTGGTACTG<br>R: AGGAGAACCACTGTTGCCTG
Myh2<br>Mus musculus	NM_001039545.2	F: AGGCGGCTGAGGAGCACGTA<br>R: GCGGCACAAGCAGCGTTGG

mRNA	RefSeq	Sequence (5' – 3')
MyH7<br>Mus musculus	NM_080728.2	F: GGAGCGCAAGTTTGTCATAAGT<br>R: CTCAAGCTGCTCAGCAATCTATTT
Ppia<br>Mus musculus	NM_008907.1	F: TGCCTTCTTTCACCTTCCCAA<br>R: TGGCAAATGCTGGACCAAAC
Ppib<br>Mus musculus	NM_011149.2	F: TCTCGGAGCGCAATATGAAGG<br>R: AGCAAAAGGAAGACGACGGA
